# ARID1A deficiency-driven reprogramming of polyamine metabolism promotes endometrial cancer malignancy and immune escape

**DOI:** 10.1038/s41419-026-08722-0

**Published:** 2026-04-08

**Authors:** Han Tao, Xiaojun Wang, Zhiyi Hu, Yiran Li, Mengwen Kong, Yeli Sun, Kun Gao, Xiaoping Wan

**Affiliations:** 1 Department of Gynecology, Shanghai First Maternity and Infant Hospital, School of Medicine, Tongji University, Shanghai, 200092 China; 2 Shanghai Key Laboratory of Maternal Fetal Medicine, Shanghai Institute of Maternal-Fetal Medicine and Gynecologic Oncology, Shanghai First Maternity and Infant Hospital, School of Medicine, Tongji University, Shanghai, 200092 China; 3 Department of Clinical Laboratory, Shanghai First Maternity and Infant Hospital, School of Medicine, Tongji University, Shanghai, 200092 China

**Keywords:** Endometrial cancer, Tumour-suppressor proteins

## Abstract

Metabolic reprogramming is crucial in developing endometrial cancer (EC); however, the mechanisms through which tumor suppressors control metabolites that drive cell proliferation and tumor growth remain unclear. ARID1A, an SWI/SNF chromatin remodeling complex subunit, is frequently mutated in endometrium-related malignancies. Here, EC tumors with ARID1A deleted exhibit increased polyamine production, which enhances malignant proliferative capacity while inhibiting the efficacy of functional CD8^+^ T cells. Mechanistically, ARID1A depletion in tumor cells interrupts the competitive binding of ARID1A to YAP, causing excessive YAP activation and transcriptionally increasing the expression of polyamine metabolic enzymes, thereby enhancing polyamine synthesis. Increased spermidine production from polyamines can directly hypusinate eukaryotic translation initiation factor 5A (eIF5A) at lysine residues, resulting in efficient histone demethylase LSD1 protein translation. Moreover, polyamine accumulation suppresses the recruitment of CD8^+^ T cells and hampers antitumor immune responses in vivo. Notably, polyamine depletion induced by eflornithine (DFMO) significantly reduces EC cell proliferative capacity and enhances CD8^+^ T-cell efficacy. Together, these findings highlight the role of ARID1A in regulating polyamine metabolism and suggest that elevated polyamine levels in tumors enhance malignant cellular behaviors and contribute to immune evasion by inhibiting CD8^+^ T cell-mediated cytotoxic responses. Therefore, targeting polyamine biosynthesis could be an important therapeutic strategy for ARID1A-inactivated EC.

## Introduction

Endometrial cancer (EC) is among the few malignancies with both steadily increasing incidence and mortality worldwide [1–3]. Projections indicate that by 2040, EC will become the third most commonly diagnosed cancer and the fourth leading cause of cancer-related death among women [4]. Despite advances in cancer diagnosis and treatment, survival outcomes for uterine cancers have remained largely stagnant over the past four decades [5, 6]. This lack of progress is attributable, at least in part, to the pronounced molecular and biological heterogeneity of EC [7, 8], which complicates therapeutic stratification and limits the efficacy of current treatments. Consequently, identifying novel therapeutic vulnerabilities and elucidating the mechanisms driving EC progression remain urgent clinical priorities.

Recent conceptual advances have established metabolic reprogramming as a hallmark of EC [9]. By reshaping metabolic pathways, tumor cells secure sufficient nutrients and biosynthetic precursors to sustain uncontrolled proliferation and invasion [10]. The consistent preclinical efficacy of targeting shared metabolic phenotypes underscores the functional relevance of metabolic subtyping and highlights metabolism as a promising therapeutic entry point [11, 12]. However, efforts to comprehensively define the metabolic drivers of tumor progression are frequently confounded by the extensive molecular heterogeneity of EC and other solid tumors [8, 10]. As a result, the mechanisms underlying metabolic reprogramming during EC progression remain incompletely understood. Among the most frequently altered genes in human cancer is ARID1A, a core subunit of the SWI/SNF chromatin remodeling complex [13]. ARID1A regulates gene expression by modulating chromatin accessibility through histone modifications, thereby enabling both transcriptional activation and repression [14]. Loss-of-function mutations in ARID1A—typically frameshift or nonsense mutations resulting in protein depletion—are detected in up to 50% of endometrial-derived neoplasms, including endometrioid and clear cell carcinomas of the uterus and ovary [15–17]. Although ARID1A is widely regarded as a tumor suppressor, its functions beyond chromatin regulation, particularly in the context of tumor metabolism, remain poorly defined. Elucidating how ARID1A inactivation contributes to tumorigenesis is therefore critical for developing effective therapies for ARID1A-mutant cancers.

Polyamines—including putrescine, spermidine, and spermine—are small, positively charged metabolites that interact with nucleic acids, proteins, phospholipids, and ion channels to regulate diverse cellular processes. Unlike classical energy metabolites, polyamines are indispensable for cell proliferation and exert regulatory effects at transcriptional, translational, and post-translational levels [18–20]. Notably, spermidine serves as the precursor for hypusine, a unique post-translational modification of eukaryotic initiation factor 5A (eIF5A) that is essential for preventing ribosomal stalling during the translation of mRNAs containing polyproline motifs and specific amino acid sequences [21]. Dysregulated polyamine metabolism is a common feature of cancer and reflects the heightened demand for polyamines during malignant transformation and tumor progression. Accumulating evidence indicates that diverse oncogenic pathways converge to sustain polyamine biosynthesis and uptake, thereby revealing polyamine metabolism as a rational therapeutic target [22–24]. Nevertheless, the molecular mechanisms driving polyamine accumulation in tumors remain largely unresolved.

In this study, we identify a previously unrecognized role of ARID1A in regulating polyamine metabolism in EC. We demonstrate that ARID1A deletion leads to polyamine accumulation through the upregulation of key biosynthetic enzymes. Elevated polyamine levels promote eIF5A hypusination, thereby enhancing the translation of LSD1 and driving malignant progression. Moreover, polyamine accumulation disrupts CD8⁺ T-cell recruitment and facilitates immune evasion within the tumor microenvironment. Collectively, our findings uncover a mechanistic link between ARID1A loss, metabolic reprogramming, translational control, and antitumor immunity, providing new insights into EC pathogenesis and highlighting polyamine metabolism as a potential therapeutic vulnerability in ARID1A-deficient tumors.

## Results

### ARID1A is frequently mutated and downregulated in endometrial cancer

Recurrent mutations in ARID1A have been reported in EC [7, 8]; however, their prevalence and clinical relevance have not been fully defined. To systematically characterize ARID1A alterations in EC, we analyzed The Cancer Genome Atlas (TCGA) pan-cancer dataset using the cBioPortal platform. Across TCGA cohorts, ARID1A alterations were observed in multiple tumor types, with EC exhibiting the highest mutation frequency, exceeding 40% (Supplementary Fig. S1A). The TCGA EC cohort included 527 patients with matched whole-exome sequencing (WXS), RNA sequencing (RNA-seq), and clinical annotation. Within this cohort, a total of 334 ARID1A mutations were identified in 231 tumors, corresponding to 43.1% of cases. These alterations consisted predominantly of truncating variants, including 140 frameshift and 113 nonsense mutations, along with 73 missense, 5 splice-site, and 3 in-frame mutations (Supplementary Fig. S1B). The mutations were distributed throughout the coding region of ARID1A, and the predominance of truncating alterations strongly suggested functional inactivation of the gene in EC. Consistent with the molecular classification defined by the TCGA consortium, ARID1A mutations were significantly enriched in microsatellite instability–high (MSI-H) and copy number–low EC subtypes (Supplementary Fig. S1C). At the transcriptomic level, ARID1A mRNA expression was markedly reduced in ARID1A-mutant tumors compared with mutation–negative cases (Supplementary Fig. S1E). Moreover, comparison of tumor and normal samples within the TCGA EC cohort revealed significantly lower ARID1A transcript levels in EC tissues relative to normal endometrium (*p* < 0.001; Supplementary Fig. S1F). At the protein level, ARID1A expression was reduced in MSI-classified EC compared with other molecular subtypes (Supplementary Fig. S1D). Survival analysis further demonstrated that patients with high ARID1A mRNA expression had significantly improved overall survival compared with those with low expression (*p* = 0.0059; Supplementary Fig. S1G).

To independently validate these findings, we analyzed 99 primary EC specimens from our institution. Sanger sequencing of all protein-coding exons identified ARID1A mutations in 52 cases, whereas 47 tumors retained wild-type ARID1A (Supplementary Fig. S1H). Consistent with TCGA data, frameshift mutations represented the most frequent alteration type in this independent cohort. Immunohistochemical analysis further confirmed that ARID1A protein expression was significantly reduced in ARID1A-mutant EC tissues compared with ARID1A–wild-type tumors (Supplementary Fig. S1I, J).

Collectively, these results demonstrate that ARID1A is frequently mutated and transcriptionally and translationally downregulated in EC, and that loss of ARID1A expression is associated with unfavorable clinical outcomes.

### Loss of ARID1A promotes malignant phenotypes in EC cells

**Fig. 1:**
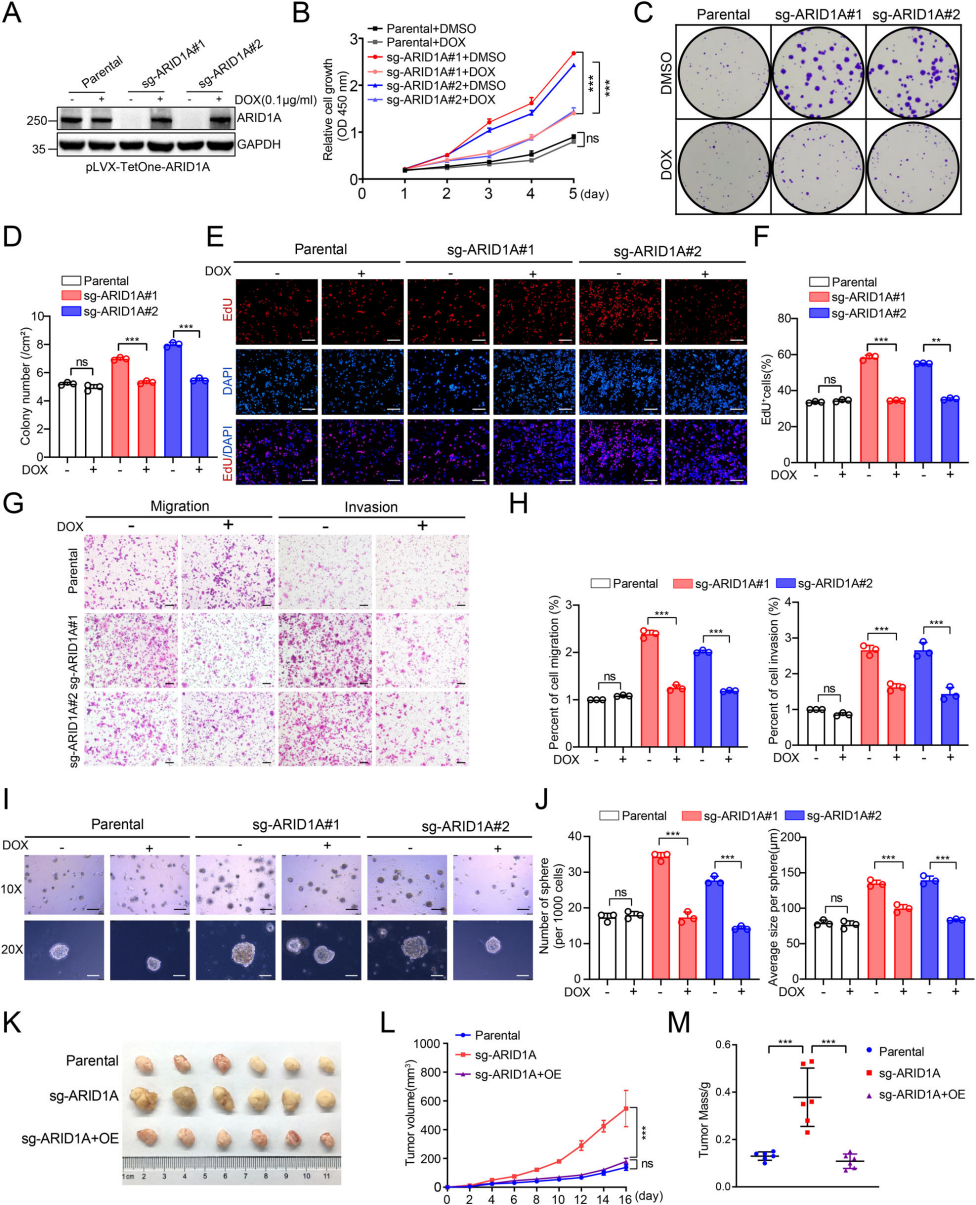
ARID1A suppresses malignant phenotypes of EC cells. **A** ARID1A KO Ishikawa cells were generated using the CRISPR/Cas9 system. Two independent ARID1A KO clones were subsequently transduced with a pLVX-TetOne-ARID1A lentiviral construct to reintroduce ARID1A in a doxycycline-inducible manner, generating ARID1A-tet-on KO cells. Thus, ARID1A-tet-on KO cells represent ARID1A-deficient clones carrying an inducible ARID1A transgene. All cell lines were treated with either DMSO or doxycycline (DOX, 100 ng/mL) for 24 h prior to cell lysis and western blot analysis. Parental Ishikawa cells were used as controls. **B** CCK-8 assays were performed in parental Ishikawa cells and ARID1A-tet-on KO cells treated with either DMSO or DOX (100 ng/mL) for the specified duration. Data are presented as mean ± SD (*n* = 3 independent biological experiments). **C**, **D** Colony formation assays were performed in parental Ishikawa cells and ARID1A-tet-on KO cells treated with either DMSO or DOX (100 ng/mL) for a duration of 2 weeks. The quantitative data are presented in (**D**). Data are presented as mean ± SD (*n* = 3 independent biological experiments). **E**, **F** EdU incorporation assays were performed in parental Ishikawa cells and ARID1A-tet-on KO cells treated with either DMSO or DOX (100 ng/mL) for 24 h. The quantitative data are presented in (**F**). Data are presented as mean ± SD (*n* = 3 independent biological experiments). Scale bar, 100 μm. **G**, **H** Transwell migration and invasion assays were performed in parental Ishikawa cells and ARID1A-tet-on KO cells treated with either DMSO or DOX (100 ng/mL) for 24 h. The quantitative data are presented in (**H**). Data are presented as mean ± SD (*n* = 3 independent biological experiments). Scale bar, 100 μm. **I**, **J** 3D sphere formation assays were performed in parental Ishikawa cells and ARID1A-tet-on KO cells treated with either DMSO or DOX (100 ng/mL) for 2 weeks. The quantitative data are presented in **J**. Data are presented as mean ± SD (*n* = 3 independent biological experiments). Scale bars: 200 μm (10×) and 100 μm (20×). **K**–**M** Parental, ARID1A-KO, or ARID1A-KO + OE Ishikawa cells were subcutaneously injected into the right flank of BALB/c nude mice. Tumor growth was measured every other day for a total of 16 days. At day 16, tumors from each group were harvested, photographed (**K**), and weighed (**M**). The volume (**L**) of the tumors was documented at each time point. Data are presented as mean ± SD (*n* = 6 mice per group). Tumor volumes were analyzed using two-way repeated-measures ANOVA with Geisser–Greenhouse correction, followed by Sidak’s multiple comparisons test. *P* values are calculated using One-way ANOVA test in (**D**, **F**, **H**, **J**, **M**) and Two-way ANOVA test in (**B**). *p < 0.05, **p < 0.01, ***p < 0.001, n.s. not significant.

We first examined ARID1A protein expression across a panel of EC cell lines. ARID1A was readily detectable in Ishikawa, KLE, and AN3CA cells, whereas its expression was minimal or absent in HEC-1A and HEC-1B cells (Supplementary Fig. S2A). Sanger sequencing confirmed that ARID1A was wild type in Ishikawa, KLE, and AN3CA cells. To investigate the functional consequences of ARID1A loss, we generated ARID1A knockout (KO) Ishikawa cells using CRISPR/Cas9-mediated gene editing with two independent single guide RNAs (sgRNAs). Complete depletion of ARID1A at both the protein and mRNA levels was verified by Western blotting and RT-qPCR analyses (Supplementary Fig. S2B–E). We next assessed the impact of ARID1A loss on EC cell growth in vitro. ARID1A KO significantly enhanced Ishikawa cell proliferation, as demonstrated by CCK-8, colony formation, and EdU incorporation assays (Fig. 1B–F; Supplementary Fig. S3A–F). Consistently, Transwell assays revealed that ARID1A depletion markedly increased cell migration and invasion (Fig. 1G, H). In addition, three-dimensional sphere formation assays showed a significant increase in both the number and size of spheres formed by ARID1A KO cells compared with parental controls (Fig. 1I, J), indicating enhanced anchorage-independent growth. To evaluate the tumor-suppressive function of ARID1A in vivo, we established xenograft models using Ishikawa cells with ARID1A knockout or re-expression. ARID1A protein levels in control, KO, and KO-overexpression groups were confirmed by Western blotting (Supplementary Fig. S2F). Consistent with the in vitro findings, ARID1A KO significantly accelerated tumor growth in nude mice, whereas re-expression of ARID1A in KO cells effectively suppressed tumor progression (Fig. 1K–M).

To exclude potential off-target effects of CRISPR/Cas9 editing, ARID1A was reintroduced into ARID1A KO Ishikawa cells using a doxycycline-inducible lentiviral system (pLVX-TetOne), restoring ARID1A expression to levels comparable to those of parental cells (Fig. 1A). Re-expression of ARID1A robustly reversed the enhanced proliferative, migratory, invasive, and sphere-forming phenotypes induced by ARID1A loss (Fig. 1B–J), confirming that these malignant features were specifically attributable to ARID1A depletion. Similar tumor-promoting effects following ARID1A knockout were observed in KLE cells (Supplementary Figs. S2G–I, S3G–L). Conversely, ectopic expression of ARID1A in ARID1A-deficient HEC-1B cells significantly suppressed malignant phenotypes (Supplementary Fig. S3M–S).

Collectively, these results demonstrate that ARID1A functions as a tumor suppressor in EC by restraining cell proliferation, invasion, and tumor growth both in vitro and in vivo.

### Loss of ARID1A reprograms polyamine metabolism in EC cells

**Fig. 2:**
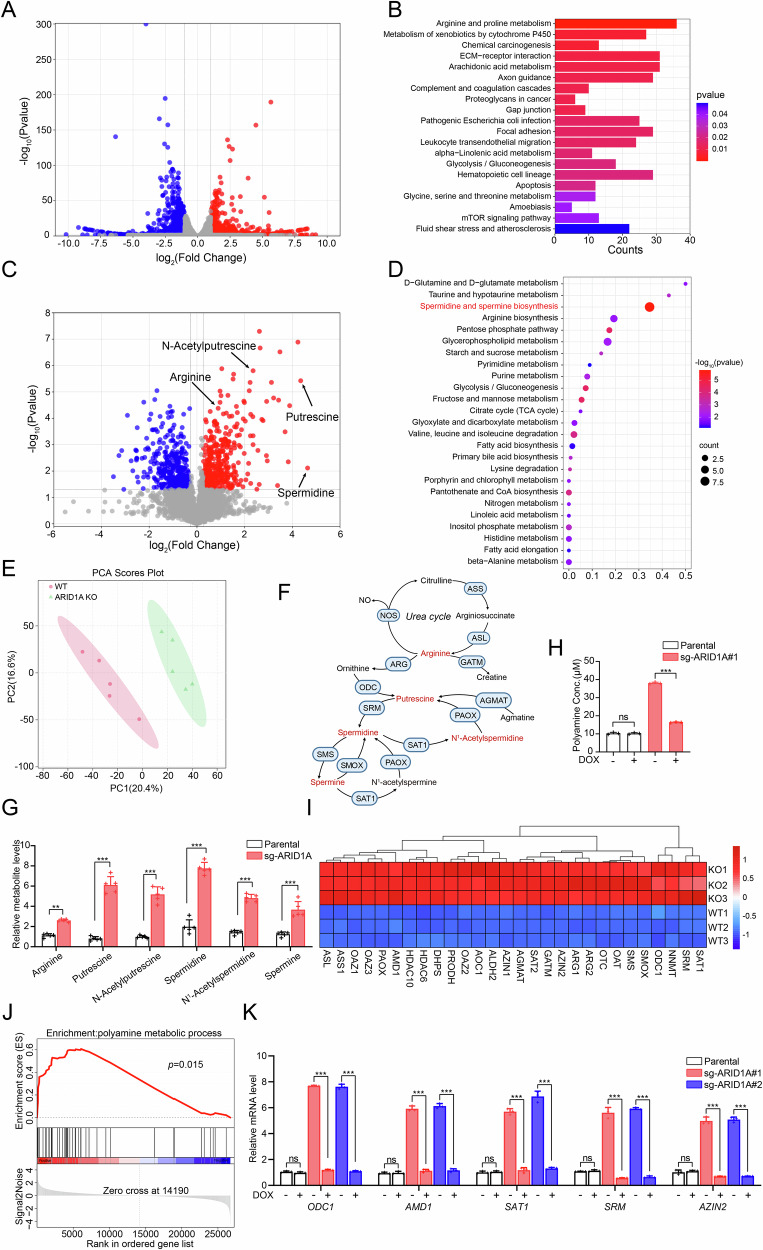
ARID1A KO leads to aberrant upregulation of polyamine biosynthesis. **A** ARID1A KO Ishikawa cells were analyzed to generate a volcano plot depicting the differential expression of genes compared to parental cells. Upregulated genes are shown in red, while downregulated genes are represented in blue. Genes with no significant changes are depicted in gray. **B** KEGG analysis was performed to examine the differentially expressed genes in ARID1A KO Ishikawa cells compared to parental cells. The analysis identified various pathways, including Arginine and proline metabolism, Glycolysis/Gluconeogenesis, and others. The number of genes enriched in each pathway is shown on the X-axis, while the color represents the enrichment significance based on the corrected *p*-value. Red indicates a low *p*-value, while blue indicates a high *p*-value. **C** The volcano plot displays alterations in metabolites observed in ARID1A KO Ishikawa cells when compared to the control group. Metabolites exhibiting a |fold change| > 1.2 and *p*-value < 0.05 are depicted in red (indicating an increase) or blue (indicating a decrease). **D** Metabolic pathway enrichment analysis was performed to investigate the upregulated metabolites in ARID1A KO Ishikawa cells. **E** PCA of untargeted metabolomics (*n* = 5 biological replicates per group). **F** The schematic diagram represents the polyamine metabolic pathway. Blue circles indicate metabolic enzymes, while red text represents metabolites. **G** The levels of metabolites related to the metabolism of polyamines were compared between parental and ARID1A KO Ishikawa cells. Data are presented as mean ± SD (*n* = 5 independent biological experiments). **H** Total polyamine levels were measured in parental and ARID1A-tet-on KO Ishikawa cells after treatment with either DMSO or DOX (100 ng/mL) for 24 h. Data are presented as mean ± SD (*n* = 3 independent biological experiments). **I** Heatmap showing the expression of 28 differentially expressed polyamine metabolic enzyme genes in parental and ARID1A KO Ishikawa cells. **J** GSEA of the polyamine metabolic gene signature in parental and ARID1A KO Ishikawa cells. The hallmark polyamine metabolic gene set was sourced from the Molecular Signatures Database (MsigDB). **K** The mRNA expression of polyamine metabolic genes was measured using RT-qPCR in parental and ARID1A-tet-on KO Ishikawa cells treated with either DMSO or DOX (100 ng/mL) for 24 h. Data are presented as mean ± SD (*n* = 3 independent biological experiments). *P* values are calculated using One-way ANOVA test in (**H**, **G**, **K**). *p < 0.05, **p < 0.01, ***p < 0.001, n.s. not significant.

To elucidate the molecular mechanisms underlying ARID1A-mediated tumor suppression in EC, we performed RNA-seq to profile global transcriptional changes in ARID1A KO Ishikawa cells. Comparative analysis identified 1,649 differentially expressed protein-coding genes, including 812 genes upregulated by more than twofold and 837 genes downregulated by more than twofold relative to parental cells (Fig. 2A). Pathway enrichment analysis using the Kyoto Encyclopedia of Genes and Genomes (KEGG) database revealed that ARID1A loss significantly altered multiple metabolic pathways, including arginine and proline metabolism, glycolysis/gluconeogenesis, and glycine, serine, and threonine metabolism (Fig. 2B), suggesting a prominent role for ARID1A in regulating cellular metabolism. To further define metabolic alterations driven by ARID1A deficiency, we performed untargeted metabolomic profiling. This analysis identified 23 metabolites that were significantly increased and 31 metabolites that were significantly decreased in ARID1A KO cells (|fold change| > 1.2, *P* < 0.05; Fig. 2C). KEGG pathway mapping of these metabolites demonstrated that polyamine metabolism represented the most significantly altered pathway in terms of the number of dysregulated metabolites (Fig. 2D). Principal Component Analysis (PCA) further confirmed a clear metabolic separation between ARID1A KO and control cells (Fig. 2E). Notably, putrescine and spermidine—two key intermediates of the polyamine biosynthetic pathway—were among the most prominently upregulated metabolites in ARID1A KO cells (Fig. 2C, F).

We next performed a focused analysis of the polyamine biosynthetic pathway, which converts arginine and ornithine into the polycationic amines putrescine, spermidine, and spermine. Quantitative metabolite profiling revealed that the majority of pathway intermediates were significantly elevated in ARID1A KO cells (Fig. 2G; Supplementary Fig. S4B), resulting in a marked increase in total intracellular polyamine levels (Fig. 2H; Supplementary Figs. S4A, S6C). These data indicate that ARID1A loss enhances polyamine biosynthesis in EC cells.

To investigate the transcriptional basis of polyamine accumulation, we examined the expression of key enzymes involved in polyamine metabolism. ARID1A KO cells exhibited significantly increased mRNA levels of ornithine decarboxylase 1 (*ODC1*), adenosylmethionine decarboxylase 1 (*AMD1*), spermidine synthase (*SRM*), and spermidine/spermine N¹-acetyltransferase 1 (*SAT1*) (Fig. 2I). Consistently, gene set enrichment analysis (GSEA) demonstrated a significant enrichment of the polyamine metabolic gene signature following ARID1A loss (Fig. 2J). Re-expression of ARID1A reversed the upregulation of these polyamine biosynthetic genes (Fig. 2K; Supplementary Figs. S4C, S6D) and reduced total polyamine levels in both Ishikawa and KLE cells (Fig. 2H; Supplementary Fig. S6C). To assess the clinical relevance of these findings, we analyzed the TCGA UCEC dataset. ARID1A expression was inversely correlated with the expression of most polyamine biosynthetic enzymes, with the exception of ODC1 (Supplementary Fig. S5A–H), further supporting a negative regulatory role of ARID1A in polyamine metabolism in human EC.

Collectively, these results demonstrate that ARID1A inactivation drives transcriptional and metabolic reprogramming of the polyamine biosynthetic pathway, leading to polyamine accumulation in EC cells.

### ARID1A interacts with YAP to restrain YAP/TEAD-dependent transcription of polyamine metabolic enzymes

**Fig. 3:**
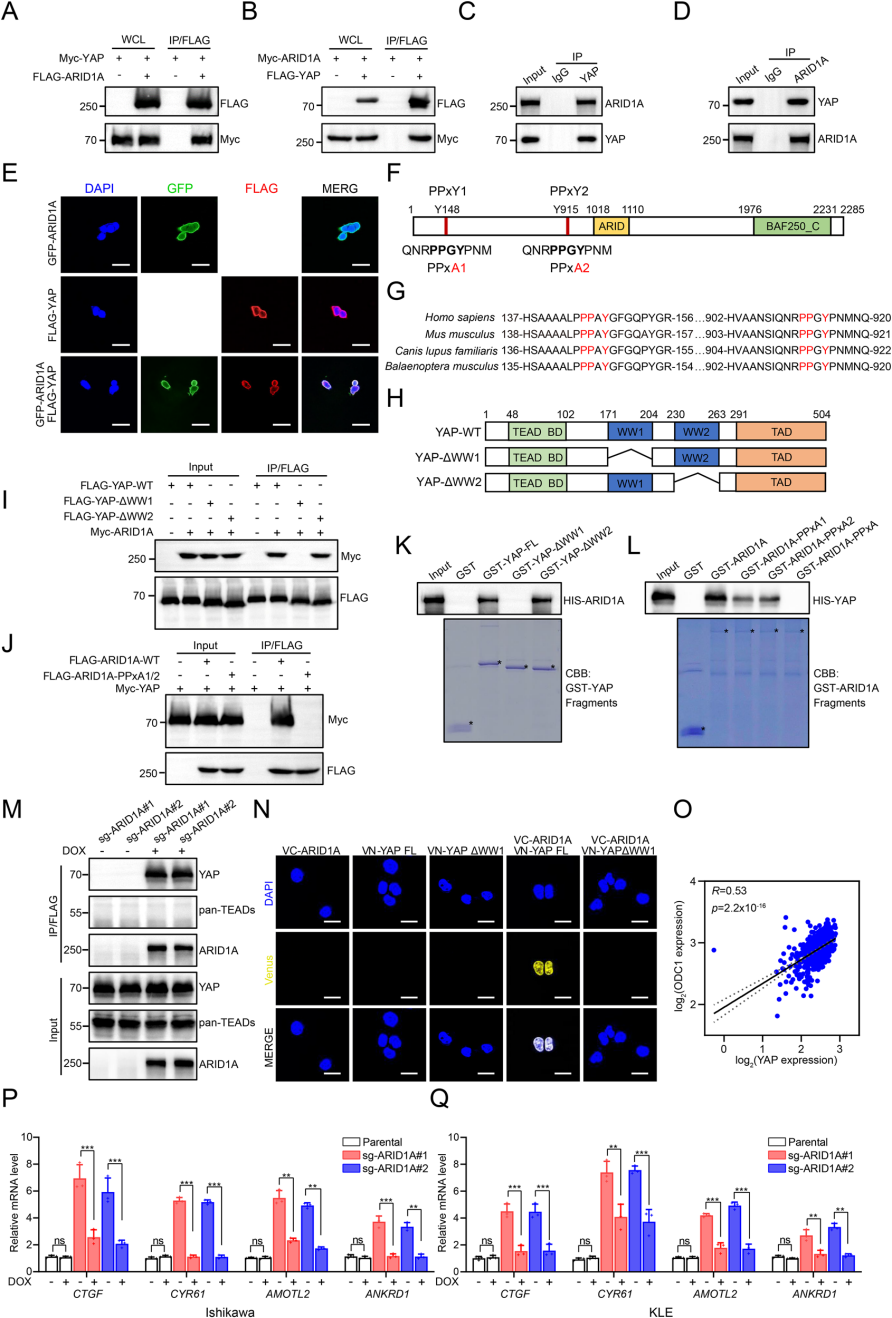
ARID1A interacts with YAP to restrain YAP/TEAD-dependent transcription of polyamine metabolic enzymes. **A** Western blot analysis was performed on WCLs and co-immunoprecipitation (Co-IP) samples derived from 293T cells transfected with specific plasmids, using an anti-FLAG antibody. **B** Western blot analysis was performed on WCLs and Co-IP samples derived from 293 T cells transfected with specific plasmids, using an anti-FLAG antibody. **C** Western blot analysis was performed on WCLs and Co-IP samples derived from cell extracts of Ishikawa cells, using IgG or anti-YAP antibody. **D** Western blot analysis was performed on WCLs and Co-IP samples derived from cell extracts of Ishikawa cells, using IgG or anti-ARID1A antibody. **E** Ishikawa cells were transfected with GFP-ARID1A and/or FLAG-YAP plasmids. After 24 h, the cells were fixed and analyzed by immunofluorescence. Scale bar: 20 μm. **F** Diagram illustrating the structural variations of ARID1A mutants. **G** Amino acid sequence alignment of the potential PPxY motifs in ARID1A orthologues. **H** Schematic representation of YAP deletion mutants showing their binding capability with ARID1A. **I** Western blot analysis showing the indicated proteins was performed on WCLs and Co-IP samples derived from 293T cells transfected with specific plasmids, using an anti-FLAG antibody. **J** Western blot analysis showing the indicated proteins was performed on WCLs and Co-IP samples derived from 293T cells transfected with specific plasmids, using an anti-FLAG antibody. **K** In vitro GST pull-down assay was conducted using His-ARID1A in combination with either GST or GST-YAP fragments. Coomassie brilliant blue (CBB) was used for visualization. Specific protein bands were indicated by an asterisk (*). **L** In vitro GST pull-down assay was conducted using His-YAP in combination with either GST or GST-ARID1A mutants. Coomassie brilliant blue (CBB) was used for visualization. Specific protein bands were indicated by an asterisk (*). **M** Co-IP assays were performed in ARID1A-tet-on KO cells treated with 100 ng/mL of DOX for 24 h. The cells were then immunoprecipitated using anti-FLAG antibody. **N** BiFC experiments were conducted using Ishikawa cells transfected with plasmids containing ARID1A fused to the fluorescent protein fragment VC155, along with YAP-WT or YAP mutants fused to the fluorescent protein fragment VN173. Scale bar: 10 μm. **O** Spearman correlation analysis was conducted to examine the relationship between YAP and ODC1 in both tumor and matched normal tissues obtained from patients with EC in the TCGA dataset (Spearman correlation coefficient, Rs = 0.53, *p* = 2.2 × 10^−16^). **P** The mRNA expression levels of YAP target genes were measured using RT-qPCR in parental and ARID1A-tet-on KO Ishikawa cells treated with either DMSO or DOX (100 ng/mL) for 24 h. Data are presented as mean ± SD (*n* = 3 independent biological experiments). **Q** The mRNA expression levels of YAP target genes were measured using RT-qPCR in parental and ARID1A-tet-on KO KLE cells treated with either DMSO or DOX (100 ng/mL) for 24 h. Data are presented as mean ± SD (*n* = 3 independent biological experiments). *P* values are calculated using One-way ANOVA test in (**P**, **Q**). *p < 0.05, **p < 0.01, ***p < 0.001, n.s. not significant.

Previous studies, including our own, have shown that the Hippo–YAP pathway is aberrantly activated in EC [25, 26], and that components of the SWI/SNF complex can associate with YAP [27]. To investigate whether ARID1A regulates polyamine metabolism through YAP signaling, we first examined potential interactions between ARID1A and YAP or TEAD transcription factors in EC cells. Co-immunoprecipitation (Co-IP) assays revealed a robust interaction between ectopically expressed ARID1A and YAP (Fig. 3A, B). Consistently, endogenous ARID1A and YAP were found to associate in Ishikawa and KLE cells (Fig. 3C, D; Supplementary Fig. S6A, B). Immunofluorescence analysis further demonstrated nuclear co-localization of ARID1A and YAP (Fig. 3E). ARID1A contains two conserved Pro–Pro–x–Tyr (PPxY) motifs within its substrate-binding domain, which are known to mediate interactions with WW domain–containing proteins (Fig. 3F). Sequence alignment revealed that these PPxY motifs are evolutionarily conserved across species (Fig. 3G). YAP, in turn, harbors two WW domains that recognize proline-rich motifs in binding partners, in addition to its C-terminal transactivation domain [28] (Fig. 3H). To delineate the structural basis of the ARID1A–YAP interaction, we generated a series of YAP deletion mutants. Co-IP assays demonstrated that deletion of the WW1 domain completely abolished YAP binding to ARID1A, whereas deletion of other domains, including WW2, had no appreciable effect (Fig. 3I). Conversely, mutation of both PPxY motifs in ARID1A eliminated its interaction with YAP (Fig. 3J). Consistent with these findings, GST pulldown assays showed that purified His-tagged ARID1A directly bound GST–YAP but not GST alone, indicating a direct interaction mediated by the YAP WW1 domain and ARID1A PPxY motifs in vitro (Fig. 3K). To further map the domains of YAP involved in the ARID1A interaction, we fragmented YAP according to its functional domains. Both the full-length (FL) YAP and its WW1 domain were found to interact with ARID1A. Similarly, we mutated ARID1A and confirmed that its two PPxY motifs mediate the interaction with YAP (Fig. 3L). These findings demonstrate the direct interaction between ARID1A and YAP. To further validate this interaction, we utilized a tet-on–inducible system. FLAG-tagged ARID1A interacted with endogenous YAP but not with TEAD family members (Fig. 3M). Bimolecular fluorescence complementation (BiFC) assays further visualized the ARID1A–YAP interaction in living cells, confirming their direct association in the nuclear compartment (Fig. 3N). Analysis of RNA-seq data from the TCGA UCEC cohort revealed that expression of ODC1, a rate-limiting enzyme in polyamine biosynthesis, was significantly upregulated in EC tumors and positively correlated with YAP mRNA levels (Fig. 3O). Consistently, ARID1A KO led to increased expression of canonical YAP target genes, including *CTGF*, *CYR61*, *AMOTL2*, and *ANKRD1*, in both Ishikawa and KLE cells (Fig. 3P, Q).

**Fig. 4:**
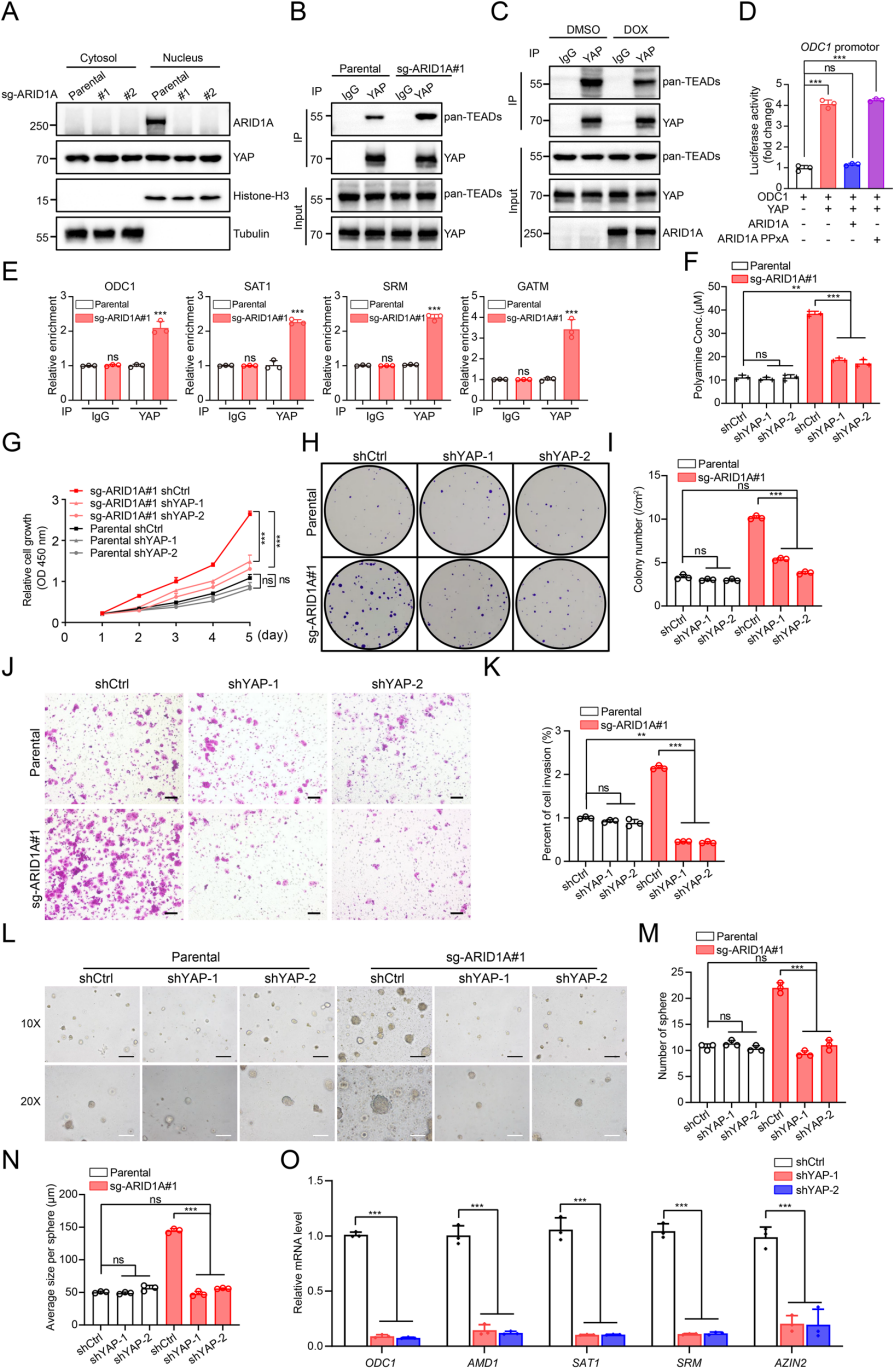
YAP is required for ARID1A loss–induced polyamine accumulation and malignant progression. **A** Western blots were performed on nuclear and cytoplasmic fractions obtained from parental and ARID1A KO Ishikawa cells. Histone H3 and β-tubulin were used as controls for the nuclear and cytosolic fractions. **B** Co-IP assays were performed in parental and ARID1A KO Ishikawa cells, followed by immunoprecipitation with either IgG or anti-YAP antibody. **C** Co-IP assays were performed in ARID1A-tet-on KO Ishikawa cells treated with either DMSO or DOX (100 ng/mL) for 24 h. The cells were then immunoprecipitated with either IgG or anti-YAP antibody. **D** Dual luciferase reporter assays were performed in 293 T cells transfected with the indicated plasmids. Luciferase activity was measured 24 h after transfection. Data are presented as mean ± SD (*n* = 3 independent biological experiments). **E** ChIP–qPCR assays were conducted on both parental and ARID1A KO Ishikawa cells, specifically targeting the promoters of *ODC1*, *SAT1*, *SRM*, and *GATM*. The results are presented as the percentage of enrichment relative to the input DNA. Data are presented as mean ± SD (*n* = 3 independent biological experiments). **F** Total polyamine levels were measured in Ishikawa cells infected with lentivirus expressing shctrl, shYAP-1, or shYAP-2, including both parental and ARID1A KO cells. Data are presented as mean ± SD (*n* = 3 independent biological experiments). **G** CCK-8 assays were performed in Ishikawa cells infected with lentivirus expressing shctrl, shYAP-1, or shYAP-2, including both parental and ARID1A KO cells. Data are presented as mean ± SD (*n* = 3 independent biological experiments). **H**, **I** Colony formation assays were performed in Ishikawa cells infected with lentivirus expressing shctrl, shYAP-1, or shYAP-2, including both parental and ARID1A KO cells. The quantitative data are presented in (**I**). Data are presented as mean ± SD (*n* = 3 independent biological experiments). **J**, **K** Transwell invasion assays were performed in Ishikawa cells infected with lentivirus expressing shctrl, shYAP-1, or shYAP-2, including both parental and ARID1A KO cells. The quantitative data are presented in (**K**). Data are presented as mean ± SD (*n* = 3 independent biological experiments). Scale bar, 100 μm. **L**–**N** 3D sphere formation assays were performed in Ishikawa cells infected with lentivirus expressing shctrl, shYAP-1, or shYAP-2, including both parental and ARID1A KO cells. The quantitative data are presented in (**M**) and (**N**). Data are presented as mean ± SD (*n* = 3 independent biological experiments). Scale bars: 200 μm (10×) and 100 μm (20×). **O** The mRNA expression of polyamine metabolic genes was measured using RT-qPCR in ARID1A KO Ishikawa cells infected with lentivirus expressing shctrl, shYAP-1, or shYAP-2. Data are presented as mean ± SD (*n* = 3 independent biological experiments). *P* values are calculated using One-way ANOVA test in (**D**, **E**, **F**, **I**, **K**, **M**, **N**, **O**) and Two-way ANOVA test in (**G**). *p < 0.05, **p < 0.01, ***p < 0.001, n.s. not significant.

Because dephosphorylated YAP translocates to the nucleus to activate transcription [29], we next assessed whether ARID1A affects YAP subcellular localization or transcriptional complex formation. Fractionation assays revealed comparable levels of nuclear YAP in parental and ARID1A KO Ishikawa cells (Fig. 4A), indicating that ARID1A loss does not alter YAP nuclear accumulation. However, Co-IP assays demonstrated a markedly enhanced interaction between YAP and TEAD in ARID1A KO cells, which was attenuated upon inducible re-expression of ARID1A (Fig. 4B, C). Given that ODC1 has been reported as a direct transcriptional target of YAP/TEAD [30], we next examined whether ARID1A suppresses ODC1 transcription through competitive binding to YAP. Luciferase reporter assays demonstrated that wild-type ARID1A significantly repressed ODC1 promoter activity, whereas an ARID1A mutant lacking functional PPxY motifs failed to do so (Fig. 4D), indicating that the ARID1A–YAP interaction is required for transcriptional repression. To globally assess YAP-dependent transcriptional regulation following ARID1A loss, we performed YAP chromatin immunoprecipitation followed by sequencing (ChIP-seq) in parental and ARID1A KO Ishikawa cells. ARID1A deficiency resulted in a genome-wide increase in YAP chromatin occupancy (Supplementary Fig. S7A). KEGG pathway analysis of YAP-bound genes revealed significant enrichment of arginine and proline metabolism pathways, which include multiple polyamine biosynthetic enzymes, specifically in ARID1A KO cells (Supplementary Fig. S7B). Consistent with these findings, ChIP–qPCR assays confirmed significantly increased YAP binding to the promoter regions of ODC1, SAT1, SRM, and GATM in ARID1A-deficient cells (Fig. 4E).

Collectively, these results demonstrate that ARID1A directly interacts with YAP via PPxY–WW domain binding, thereby limiting YAP–TEAD complex formation and restraining YAP-driven transcription of polyamine metabolic enzymes.

### YAP is required for ARID1A loss–induced polyamine accumulation and malignant progression

To determine whether polyamine accumulation induced by ARID1A loss depends on aberrant YAP activation, we stably depleted YAP in ARID1A KO Ishikawa cells using two independent shRNAs (Supplementary Fig. S8A, B). YAP knockdown significantly reduced total intracellular polyamine levels in ARID1A KO cells compared with control shRNA–treated cells (Fig. 4F). Consistently, suppression of YAP markedly decreased the mRNA expression of key enzymes involved in polyamine biosynthesis (Fig. 4O; Supplementary Fig. S8C). Functionally, YAP depletion significantly attenuated the malignant phenotypes induced by ARID1A loss. Specifically, knockdown of YAP significantly suppressed cell proliferation, as demonstrated by both CCK-8 and colony formation assays (Fig. 4G–I), and markedly inhibited cell invasion in Transwell assays (Fig. 4J, K). In addition, YAP silencing markedly suppressed three-dimensional sphere formation, as reflected by reductions in both sphere number and size (Fig. 4L–N). These inhibitory effects were specific to ARID1A-deficient cells and were not observed in parental Ishikawa cells, indicating that YAP activity is dispensable for basal malignant behavior but becomes critical following ARID1A loss.

To complement the genetic approach, we pharmacologically inhibited YAP activity using verteporfin, a clinically approved compound that disrupts the YAP–TEAD interaction and blocks YAP-dependent transcription [31, 32]. Treatment of ARID1A KO cells with verteporfin phenocopied the effects of YAP knockdown, leading to a significant reduction in polyamine levels (Supplementary Fig. S9A), downregulation of polyamine metabolic enzymes (Supplementary Fig. S9B), and suppression of proliferative and invasive phenotypes (Supplementary Fig. S9C–E).

Collectively, these results demonstrate that YAP activity is required for ARID1A loss–driven polyamine accumulation and malignant progression in EC cells.

### Spermidine drives eIF5A hypusination to accelerate LSD1 protein translation in ARID1A-deficient EC cells

**Fig. 5:**
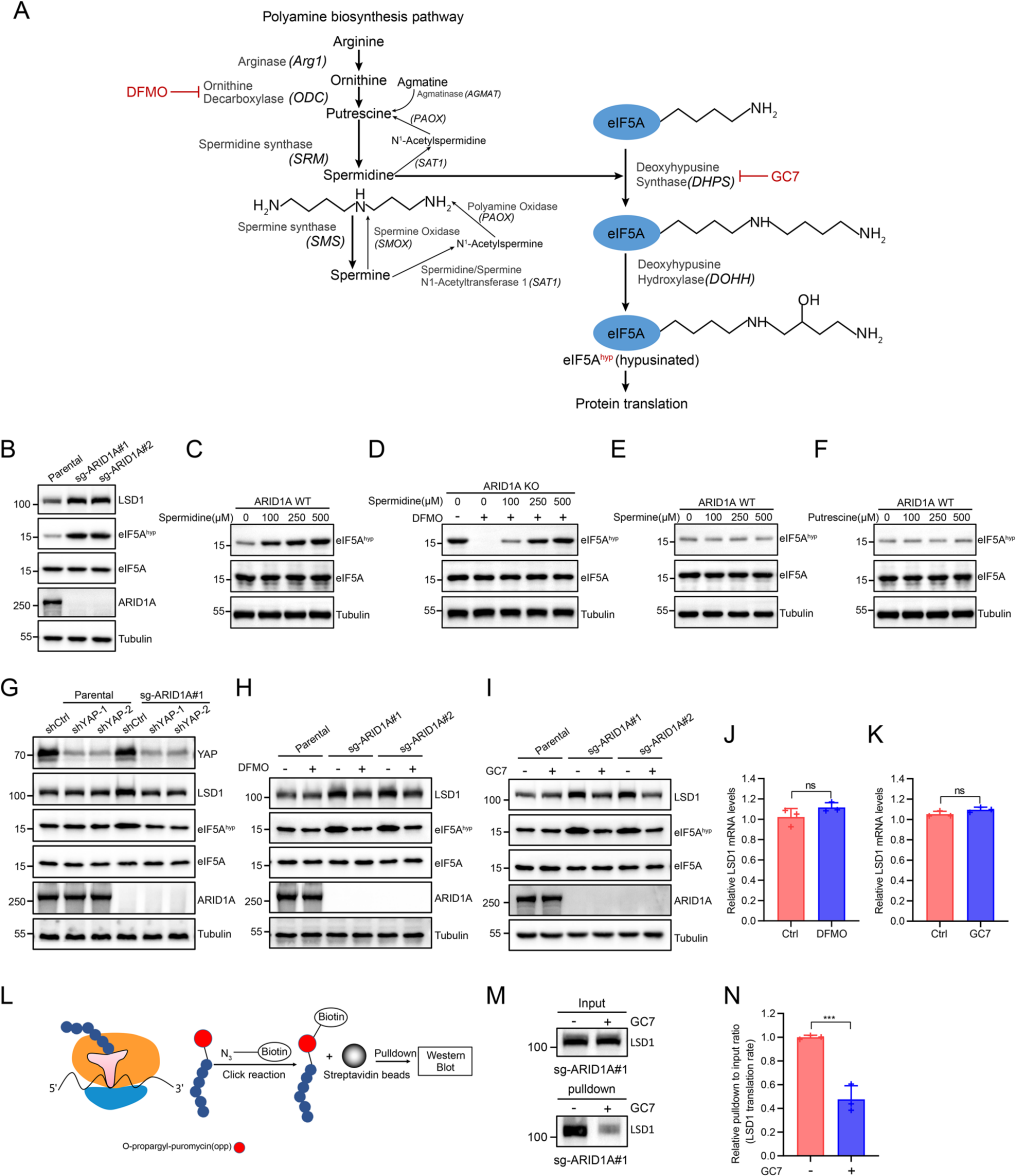
Spermidine drives eIF5A hypusination to accelerate LSD1 protein translation in ARID1A-deficient EC cells. **A** Schematic representation of the polyamine metabolism pathway. **B** Western blot analysis was performed to detect the indicated proteins in both parental and ARID1A KO Ishikawa cells. **C** Western blot analysis showing the indicated protein expression levels in Ishikawa cells treated with various concentrations of Spermidine for 24 h. **D** Western blot analysis showing the indicated protein expression levels after 24-h treatment with different concentration gradients of Spermidine following polyamine synthesis inhibition by DFMO (3 mM) in ARID1A KO Ishikawa cells. **E** Western blot analysis showing the indicated protein expression levels in Ishikawa cells treated with various concentrations of Spermine for 24 h. **F** Western blot analysis showing the indicated protein expression levels in Ishikawa cells treated with various concentrations of Putrescine for 24 h. **G** Western blot analysis was performed to detect the indicated proteins in Ishikawa cells infected with lentivirus expressing shctrl, shYAP-1, or shYAP-2, including both parental and ARID1A KO cells. **H** Western blot analysis was performed to detect the indicated proteins in parental and ARID1A KO Ishikawa cells treated with either DMSO or DFMO (2 mM) for 48 h. **I** Western blot analysis was performed to detect the indicated proteins in parental and ARID1A KO Ishikawa cells treated with either DMSO or GC7 (10 μM) for 24 h. **J** RT-qPCR was used to examine the levels of LSD1 mRNA in ARID1A KO Ishikawa cells treated with either DMSO or DFMO (2 mM) for 48 h. Data are presented as mean ± SD (*n* = 3 independent biological experiments). **K** RT-qPCR was used to examine the levels of LSD1 mRNA in ARID1A KO Ishikawa cells treated with either DMSO or GC7 (10 μM) for 24 h. Data are presented as mean ± SD (*n* = 3 independent biological experiments). **L** Nascent protein synthesis was assessed using the OPP pulldown assay. OPP, which is incorporated into newly synthesized proteins, is subsequently linked to an alkyne group on OPP via a click reaction with the azide group on biotin. The biotin-labeled proteins are captured by magnetic streptavidin beads and identified through Western blot analysis. **M**, **N** The levels of newly translated LSD1 proteins were measured in ARID1A KO Ishikawa cells treated with either 10 μM GC7 or DMSO (control) for 24 h. Data are presented as mean ± SD (*n* = 3 independent biological experiments). *P* values are calculated using One-way ANOVA test in (**J**, **K**, **N**). *p < 0.05, **p < 0.01, ***p < 0.001, n.s. not significant.

Given the essential role of polyamine accumulation in ARID1A loss–driven malignant phenotypes, we next investigated the downstream molecular mechanisms linking polyamines to tumor progression. Spermidine serves as the obligate substrate for the hypusination of eukaryotic translation initiation factor 5A (eIF5A), a unique post-translational modification catalyzed sequentially by deoxyhypusine synthase (DHPS) and deoxyhypusine hydroxylase (DOHH) [33–35] (Fig. 5A). Consistent with elevated intracellular spermidine levels, ARID1A KO cells exhibited a marked increase in hypusinated eIF5A (eIF5A^hyp^) compared with parental cells (Fig. 5B). To directly assess the effect of spermidine on eIF5A hypusination, wild-type Ishikawa cells were treated with increasing concentrations of spermidine. Western blot analysis revealed a dose-dependent increase in eIF5A^hyp^ levels (Fig. 5C). In contrast, treatment with other polyamines, including spermine or putrescine, did not significantly enhance eIF5A hypusination (Fig. 5E, F), indicating that spermidine is the primary polyamine driving this modification. Moreover, inhibition of endogenous polyamine synthesis using difluoromethylornithine (DFMO) abolished eIF5A hypusination in ARID1A KO cells, whereas supplementation with spermidine fully restored eIF5A^hyp^ levels (Fig. 5D), demonstrating that elevated spermidine directly mediates eIF5A hypusination in ARID1A-deficient cells. Previous studies have shown that eIF5A hypusination facilitates the translation of proteins containing ribosome-stalling motifs and promotes efficient translation termination [33, 36–39]. Notably, the histone demethylase LSD1 has been reported to depend on eIF5A^hyp^ for efficient translation and functions as a transcriptional regulator of YAP/TAZ-repressed genes [30]. We therefore examined LSD1 expression following ARID1A loss. While LSD1 mRNA levels remained largely unchanged, LSD1 protein abundance was markedly increased in ARID1A KO cells, concomitant with elevated eIF5A^hyp^ (Fig. 5B). Importantly, YAP depletion in ARID1A KO cells reduced both eIF5A hypusination and LSD1 protein levels to those observed in parental cells (Fig. 5G), linking YAP activation to spermidine–eIF5A–LSD1 signaling.

To determine whether spermidine-driven eIF5A hypusination directly promotes LSD1 translation, ARID1A KO cells were treated with DFMO or GC7, an inhibitor of DHPS that specifically blocks eIF5A hypusination. Both treatments significantly reduced eIF5A hyp and LSD1 protein levels (Fig. 5H, I) without affecting LSD1 mRNA expression (Fig. 5J, K), indicating post-transcriptional regulation. To directly measure nascent LSD1 protein synthesis, we performed O-propargyl-puromycin (OPP)–based labeling and pulldown assays. GC7 treatment markedly decreased newly synthesized LSD1 protein in ARID1A KO cells (Fig. 5L–N), confirming that eIF5A hypusination enhances LSD1 translation. To define the functional consequences of increased LSD1 translation in ARID1A-deficient cells, we performed RNA-seq analysis of ARID1A KO cells treated with the LSD1 inhibitor seclidemstat [40, 41]. LSD1 inhibition induced extensive transcriptional reprogramming, with thousands of genes differentially expressed (Supplementary Fig. S10A, B). Genes downregulated upon LSD1 inhibition were strongly enriched for pathways associated with cell proliferation, migration, invasion, and multiple cancer-related signaling programs (Supplementary Fig. S10C, D). RT–qPCR validation confirmed the LSD1 dependence of representative malignancy-associated genes (Supplementary Fig. S10E, F). These results demonstrate that elevated LSD1 activity in ARID1A-deficient cells sustains transcriptional programs that promote malignant phenotypes. Consistently, seclidemstat treatment significantly suppressed the growth of ARID1A KO Ishikawa and KLE cells (Supplementary Fig. S11A, B), indicating that LSD1 activity contributes to ARID1A loss–induced malignant phenotypes.

Finally, to genetically link ARID1A–YAP interaction to spermidine–eIF5A–LSD1 signaling, we reintroduced wild-type ARID1A into ARID1A KO cells, which restored eIF5A^hyp^ and LSD1 protein levels to those of parental cells (Supplementary Fig. S13A). In contrast, CRISPR/Cas9-mediated knock-in of PPxY-disrupting ARID1A mutants (Y148A and Y915A) resulted in increased polyamine levels, elevated eIF5A hypusination, and enhanced LSD1 protein expression (Supplementary Figs. S12A, B, S13B, C). These knock-in cells also exhibited increased expression of polyamine biosynthetic genes and augmented proliferative and invasive capacities (Supplementary Figs. S12C, S13D–I).

To determine whether direct ARID1A–YAP interaction is required for ARID1A-mediated tumor suppression, we re-expressed an ARID1A PPxA mutant lacking the YAP-binding PPxY motif in ARID1A-knockout cells (Supplementary Fig. S14A). In contrast to wild-type ARID1A, the PPxA mutant failed to disrupt YAP–TEAD complex formation, reduce intracellular polyamine levels, or suppress hyperproliferation and invasion (Supplementary Fig. S14B–H). These results indicate that PPxY-dependent binding to YAP is essential for ARID1A to restrain YAP activity, polyamine accumulation, and malignant phenotypes.

Collectively, these results demonstrate that ARID1A loss promotes spermidine accumulation, leading to enhanced eIF5A hypusination and accelerated LSD1 protein translation, thereby sustaining transcriptional programs that drive malignant progression in EC cells.

### Targeting polyamine biosynthesis suppresses ARID1A loss–driven EC cell proliferation

**Fig. 6:**
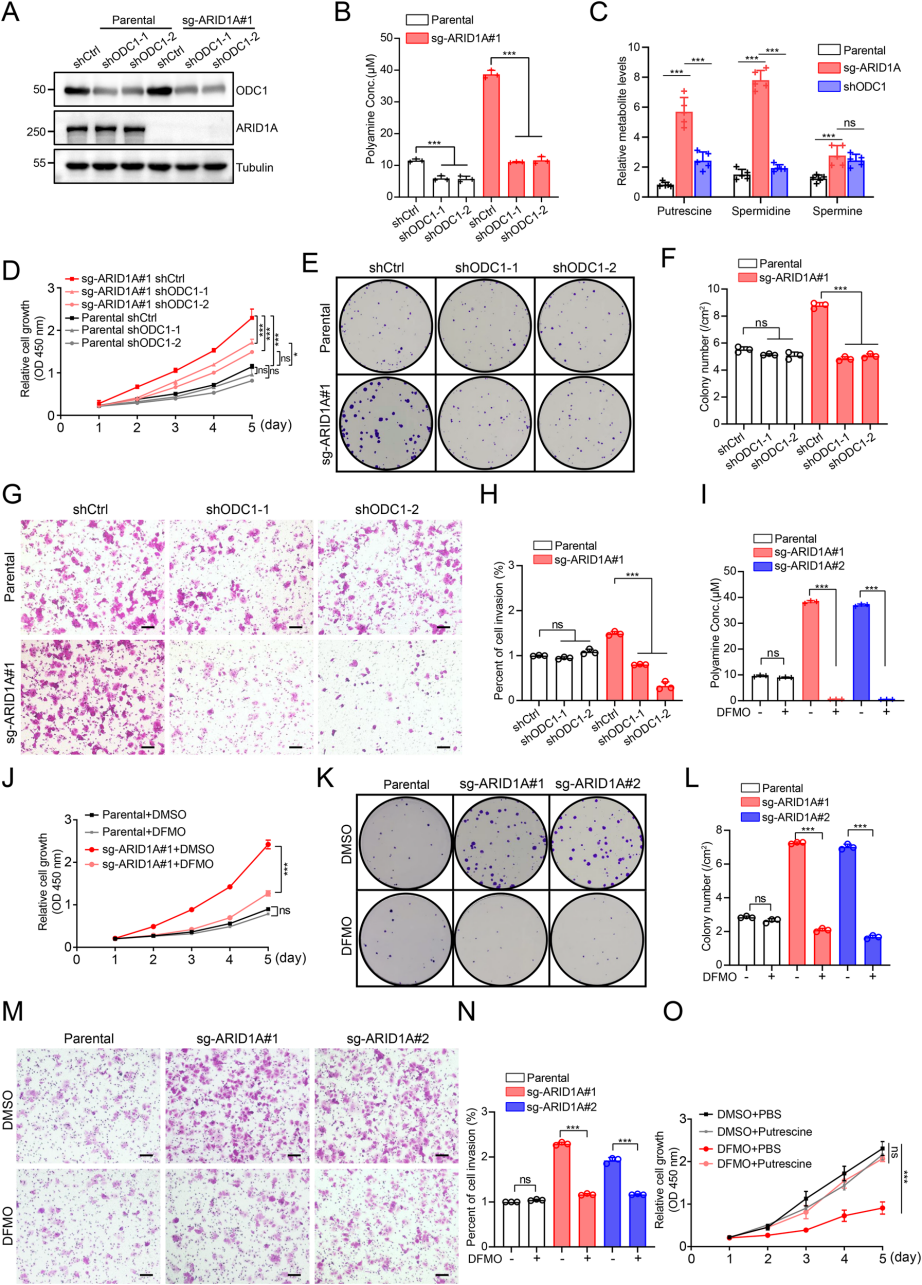
Targeting polyamine biosynthesis suppresses ARID1A loss–driven EC cell proliferation. **A** Western blot analysis was performed to examine the specified proteins in Ishikawa cells infected with lentivirus expressing shctrl, shODC1-1, or shODC1-2, including both parental and ARID1A KO cells. **B** Total polyamine levels were measured in Ishikawa cells infected with lentivirus expressing shctrl, shODC1-1, or shODC1-2, including both parental and ARID1A KO cells. Data are presented as mean ± SD (*n* = 3 independent biological experiments). **C** Relative levels of specific polyamines. Data are presented as mean ± SD (*n* = 5 independent biological experiments). **D** CCK-8 assays were performed in Ishikawa cells infected with lentivirus expressing shctrl, shODC1-1, or shODC1-2, including both parental and ARID1A KO cells. Data are presented as mean ± SD (*n* = 3 independent biological experiments). **E**, **F** Colony formation assays were performed in Ishikawa cells infected with lentivirus expressing shctrl, shODC1-1, or shODC1-2, including both parental and ARID1A KO cells. The quantitative data are presented in (**F**). Data are presented as mean ± SD (*n* = 3 independent biological experiments). **G**, **H** Transwell invasion assays were performed in Ishikawa cells infected with lentivirus expressing shctrl, shODC1-1, or shODC1-2, including both parental and ARID1A KO cells. The quantitative data are presented in (**H**). Data are presented as mean ± SD (*n* = 3 independent biological experiments). Scale bar, 100 μm. **I** Total polyamine levels were measured in parental and ARID1A KO Ishikawa cells treated with either DMSO or DFMO (2 mM) for 48 h. Data are presented as mean ± SD (*n* = 3 independent biological experiments). **J** CCK-8 assays were performed in Ishikawa cells treated with either DMSO or DFMO (2 mM) for the specified duration, including both parental and ARID1A KO cells. Data are presented as mean ± SD (*n* = 3 independent biological experiments). **K**, **L** Colony formation assays were performed in Ishikawa cells treated with either DMSO or DFMO (2 mM) for the specified duration, including both parental and ARID1A KO cells. The quantitative data are presented in (**L**). Data are presented as mean ± SD (*n* = 3 independent biological experiments). **M**, **N** Transwell invasion assays were performed in Ishikawa cells treated with either DMSO or DFMO (2 mM) for 24 h, including both parental and ARID1A KO cells. The quantitative data are presented in (**N**). Data are presented as mean ± SD (*n* = 3 independent biological experiments). Scale bar, 100 μm. **O** CCK-8 assays were performed in ARID1A KO Ishikawa cells treated with DMSO, DFMO (2 mM), putrescine (1 mM), or DFMO (2 mM) plus putrescine (1 mM) for the specified duration. Data are presented as mean ± SD (*n* = 3 independent biological experiments). *P* values are calculated using One-way ANOVA test in (**B**, **C**, **F**, **H**, **I**, **L**, **N**) and Two-way ANOVA test in (**D**, **J**, **O**). *p < 0.05, **p < 0.01, ***p < 0.001, n.s. not significant.

To determine whether elevated polyamine levels are functionally required for ARID1A loss–induced malignant progression, we genetically and pharmacologically disrupted polyamine biosynthesis in ARID1A KO EC cells. We first stably depleted ODC1, the rate-limiting enzyme in polyamine biosynthesis, in ARID1A KO Ishikawa cells using two independent shRNAs (Fig. 6A). ODC1 knockdown markedly reduced total intracellular polyamine levels that were elevated by ARID1A loss (Fig. 6B) and significantly reversed the accumulation of individual polyamines, including putrescine, spermidine, and spermine (Fig. 6C). Functionally, ODC1 depletion substantially attenuated the malignant phenotypes induced by ARID1A loss. Specifically, knockdown of ODC1 significantly suppressed the enhanced proliferation and invasive capacity of ARID1A KO Ishikawa cells in vitro (Fig. 6D–H), indicating that ODC1-mediated polyamine biosynthesis is required for ARID1A deficiency–driven tumorigenic behaviors.

Consistent with the genetic inhibition results, pharmacologic blockade of polyamine synthesis using DFMO, a selective ODC1 inhibitor, significantly suppressed the increased proliferative capacity of ARID1A KO Ishikawa cells (Fig. 6I–N). Importantly, the anti-proliferative effect of DFMO was reversed by supplementation with putrescine, the direct enzymatic product of ODC1 (Fig. 6O), confirming the on-target effect of ODC1 inhibition. To further establish polyamine dependency, we performed metabolic rescue experiments using spermidine, the key polyamine responsible for eIF5A hypusination. While DFMO treatment markedly inhibited proliferation in ARID1A-deficient cells, exogenous spermidine supplementation effectively restored cell growth (Supplementary Fig. S15), identifying spermidine as a critical downstream effector of polyamine-driven proliferation.

Collectively, these results demonstrate that ODC1-dependent polyamine biosynthesis is essential for ARID1A loss–induced malignant proliferation in EC cells, highlighting polyamine metabolism as a potential therapeutic vulnerability in ARID1A-deficient EC.

### Polyamine accumulation impairs CD8^+^ T-cell–mediated antitumor immunity

**Fig. 7:**
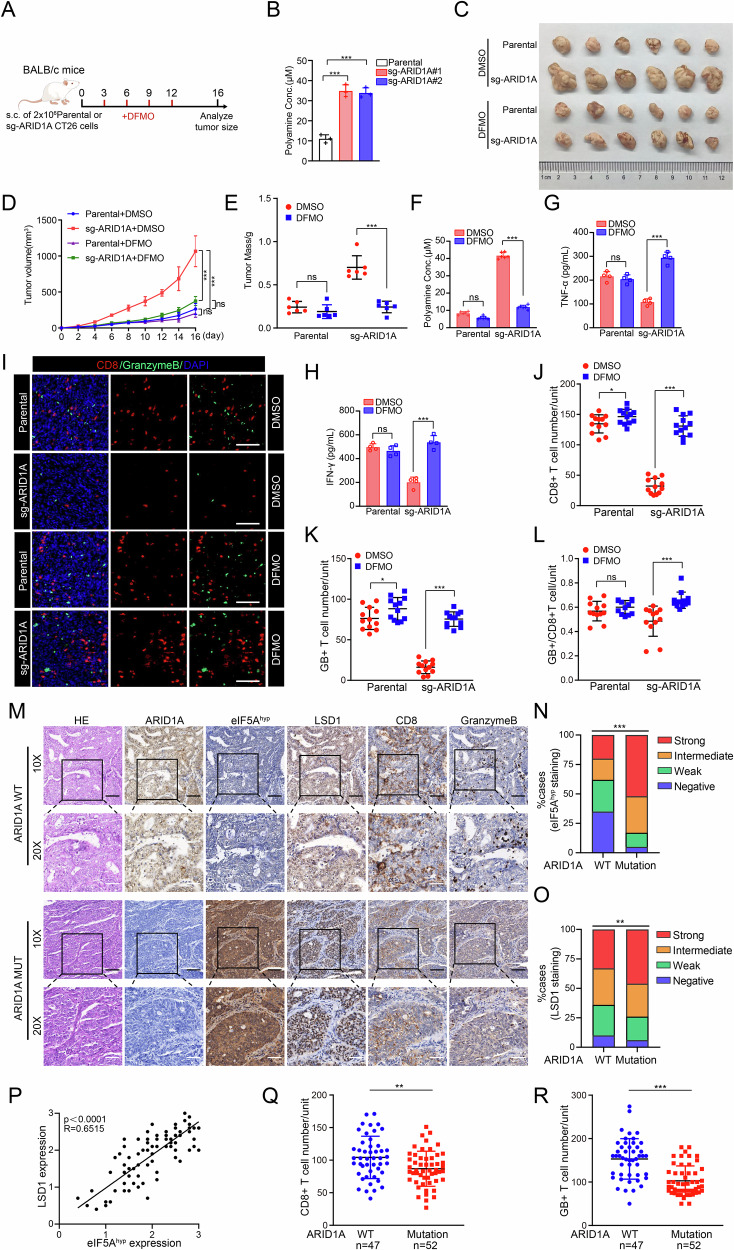
Polyamine accumulation impairs CD8^+^ T-cell–mediated antitumor immunity. **A** Diagram illustrating the treatment strategy in the syngeneic tumor model. **B** Total polyamine levels were measured in parental and ARID1A KO CT26 cells. Data are presented as mean ± SD (*n* = 3 independent biological experiments). **C**–**E** Parental and ARID1A KO CT26 cells were subcutaneously injected into the right flank of BALB/c mice, with respective treatments of DFMO or DMSO at various time intervals. Tumor growth was measured every other day over a 16-day period. On day 16, tumors from each group were collected and photographed (**C**). Tumor volume (**D**) was documented at each time point, while tumor weight (**E**) was measured on day 16. Data are presented as mean ± SD (*n* = 6 mice per group). Tumor volumes were analyzed using two-way repeated-measures ANOVA with Geisser–Greenhouse correction, followed by Sidak’s multiple comparisons test. **F** Total polyamine levels were measured in tumor tissues derived from parental and ARID1A KO CT26 cells. Data are presented as mean ± SD (*n* = 6 independent biological experiments). **G**, **H** Parental and ARID1A KO CT26 cells, treated with DMSO or DFMO, were co-cultured with activated CD8^+^ T cells isolated from BALB/c mice for 24 h. The supernatants from the co-cultures were collected, and the cytokine levels of TNF-α (**G**) and IFN-γ (**H**) were measured using ELISA kits. Data are presented as mean ± SD (*n* = 4 independent biological experiments). **I**–**L** Immunostaining was performed to detect the presence of CD8 (a marker of cytotoxic T lymphocytes) and granzyme B (a marker of T cell activity) in the CT26 tumor tissue. Data are presented as mean ± SD (*n* = 12 independent biological experiments). Scale bar, 100 μm. 3 tissue slides per tumor, 4 mice per group. Unit = 412,549 μm^2^. **M**–**R** Representative images showing HE staining and immunohistochemical staining for ARID1A, eIF5A^hyp^, LSD1, CD8, and granzyme B in EC samples with ARID1A-WT and ARID1A mutations. The correlation between eIF5A^hyp^ and LSD1 expression in EC specimens is shown in (**P**) (*n* = 99, Pearson Chi-square, *P* < 0.0001). Quantitative staining data are presented in (**N**, **O**, **Q**, **R**). Scale bars, 10× image: 50 μm, 20× image: 25 μm. *P* values are calculated using One-way ANOVA test in (**B**, **E**, **F**, **G**, **H**, **J**, **K**, **L**, **Q**, **R**) and Two-way ANOVA test in (**N**, **O**). *p < 0.05, **p < 0.01, ***p < 0.001, n.s. not significant.

We next investigated whether ARID1A loss–induced polyamine accumulation influences antitumor immunity in vivo. Specifically, we asked whether the growth advantage conferred by ARID1A deficiency depends on the presence of a functional immune system. To address this, we generated ARID1A KO CT26 murine colon carcinoma cells using CRISPR/Cas9-mediated gene editing with two independent sgRNAs (Supplementary Fig. S16A–C). When implanted into immunocompetent mice, ARID1A KO CT26 tumors exhibited significantly accelerated growth compared with parental controls (Fig. 7A, C), and notably, ARID1A knockout in CT26 cells led to elevated polyamine levels (Fig. 7B). Pharmacologic inhibition of polyamine biosynthesis using DFMO significantly suppressed tumor growth and reduced polyamine levels in ARID1A-deficient tumors, largely abolishing the difference in tumor burden between parental and ARID1A KO groups (Fig. 7D–F). To further establish disease relevance in an endometrial cancer context, we evaluated this axis in a human PBMC–humanized mouse model engrafted with EC cells. Consistent with the CT26 model, ARID1A deficiency enhanced tumor growth (Supplementary Fig. S17A–C), increased intratumoral polyamine levels (Supplementary Fig. S17D), and impaired CD8⁺ T-cell infiltration and function (Supplementary Fig. S17E–H), all of which were effectively restored by DFMO treatment. These results indicate that ARID1A loss promotes polyamine-dependent tumor growth in vivo and that inhibition of polyamine biosynthesis restores antitumor immune control.

Given the central role of tumor-infiltrating lymphocytes in antitumor immunity and immunotherapeutic efficacy [22, 42], we next examined the impact of tumor-derived polyamines on CD8⁺ T-cell function. Activated CD8⁺ T cells were cultured with conditioned media derived from parental or ARID1A KO Ishikawa cells, with or without DFMO treatment. Conditioned media from ARID1A-deficient cells markedly suppressed CD8⁺ T-cell activation, as evidenced by reduced CD69 expression and diminished production of the effector cytokines IFN-γ and TNF-α (Supplementary Fig. S18B, D). In contrast, CD8⁺ T-cell proliferation and expression of exhaustion markers were not significantly altered (Supplementary Fig. S18A, C), indicating a selective impairment of functional activation. Importantly, inhibition of polyamine synthesis in ARID1A KO cells by DFMO fully restored CD8⁺ T-cell activation and cytokine secretion. These results demonstrate that ARID1A loss–associated polyamine accumulation directly impairs CD8⁺ T-cell functional activation and cytotoxic effector responses. Consistent with these findings, ELISA analysis of co-culture supernatants demonstrated that ARID1A deficiency significantly reduced IFN-γ and TNF-α secretion by CD8⁺ T cells, whereas DFMO treatment reversed this suppression (Fig. 7G, H). We further focused on cytotoxic CD8⁺ T lymphocytes (CTLs), which mediate tumor cell killing through granzyme B (GB) release. DFMO treatment significantly increased both the abundance of intratumoral CD8⁺ CTLs and their cytotoxic activity, as reflected by approximately 2.3-fold (±0.52) and 3.25-fold (±0.35) increases in CTL numbers and GB release, respectively (Fig. 7I–L). These data indicate that tumor-derived polyamines impair CD8⁺ T-cell recruitment and cytotoxic effector function within the tumor microenvironment.

To validate the clinical relevance of these findings, we performed immunohistochemical analysis of eIF5A^hyp^ and LSD1 expression in 99 primary EC specimens. Approximately 55% of ARID1A-mutant tumors exhibited strong or intermediate staining for both eIF5A^hyp^ and LSD1, whereas fewer than 30% of ARID1A–wild-type tumors showed comparable expression (Fig. 7M–O). A strong positive correlation between eIF5A^hyp^ and LSD1 expression was observed (Pearson’s r = 0.6515; Fig. 7P). Notably, ARID1A-mutant tumors displayed significantly fewer infiltrating CD8⁺ CTLs and reduced granzyme B release compared with ARID1A–wild-type tumors (Fig. 7Q, R), supporting an association between ARID1A loss, polyamine-driven translational reprogramming, and impaired antitumor immunity in human EC.

Collectively, these results demonstrate that ARID1A loss–induced polyamine accumulation suppresses CD8⁺ T-cell–mediated antitumor immune responses, thereby facilitating immune evasion and tumor progression.

## Discussion

Tumor cells meet their increased bioenergetic and biosynthetic demands through coordinated, gene-driven reprogramming of multiple metabolic pathways [12, 43]. Identification of key regulators governing these metabolic alterations not only deepens our understanding of tumor biology but also facilitates the discovery of actionable therapeutic vulnerabilities [44]. EC exemplifies this challenge, as its development and progression are shaped by a complex interplay of genetic, molecular, and metabolic alterations. Among these, ARID1A is one of the most frequently mutated tumor suppressors, with loss-of-function alterations occurring in nearly half of ECs. Although ARID1A mutations have been implicated in diverse oncogenic processes—including PI3K/AKT signaling, DNA damage repair, and immune regulation [45]—the extent to which ARID1A contributes to metabolic reprogramming in EC has remained largely unexplored.

**Fig. 8:**
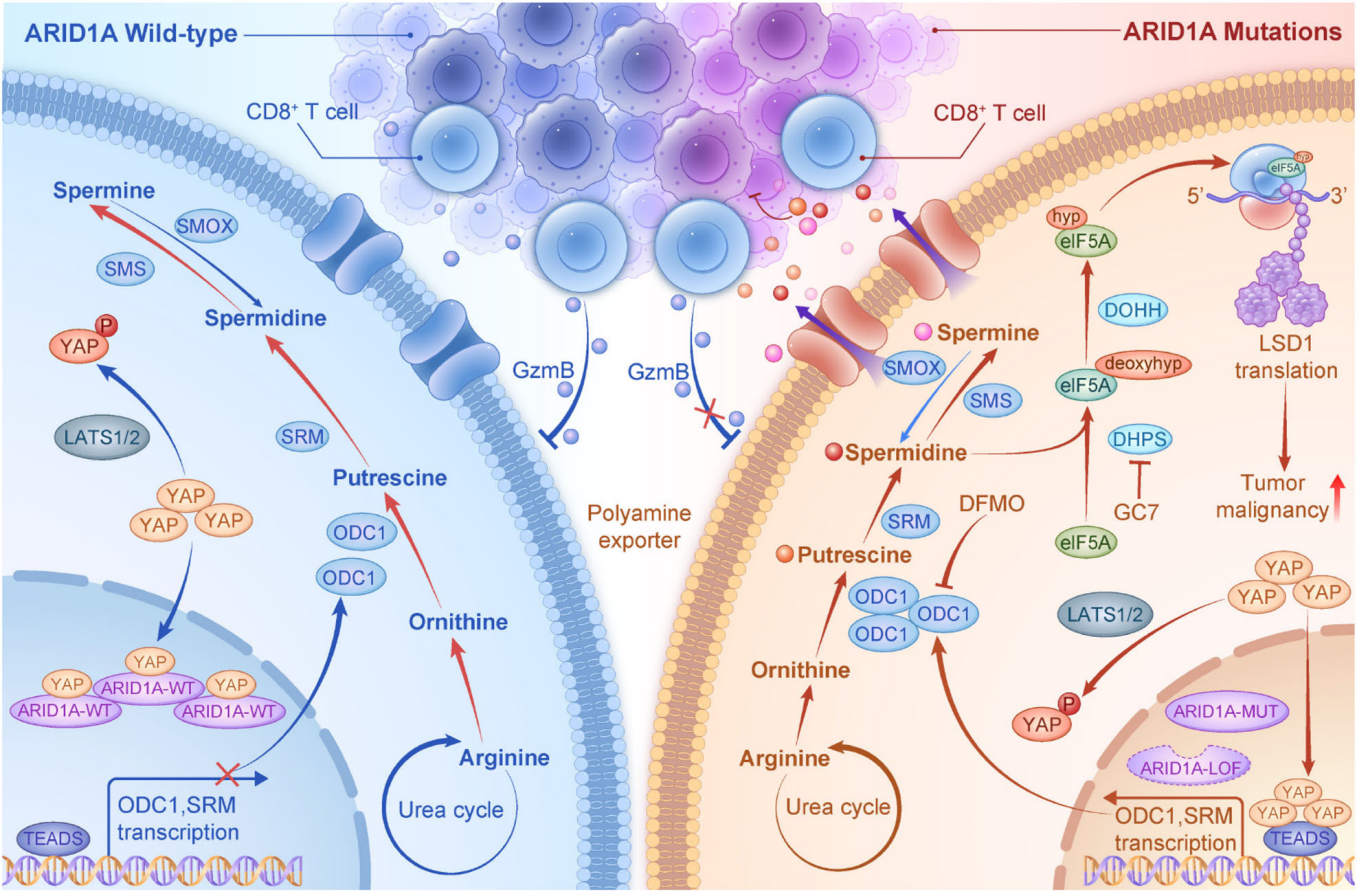
Schematic of the proposed mechanism by which ARID1A mutations enhance the malignant progression of EC via polyamine metabolism. In ARID1A wild-type endometrial cancer cells, ARID1A binds competitively with YAP in the nucleus, preventing the formation of the YAP-TEAD complex and inhibiting the transcription of downstream polyamine biosynthesis target genes, such as ODC1. However, in ARID1A-mutant endometrial cancer, the loss or inactivation of ARID1A leads to YAP hyperactivation, which promotes the transcriptional activation of polyamine biosynthesis enzymes and catalyzes the production of polyamines like putrescine and spermidine. Spermidine, in turn, promotes the hypusination of eIF5A, thereby enhancing the translation of the downstream target protein LSD1, contributing to the malignant progression of endometrial cancer. Additionally, the accumulation of polyamines derived from tumor cells inhibits the activation of CD8^+^ T cells in the tumor microenvironment, enabling tumor cells to evade immune surveillance. Therefore, targeting polyamine synthesis may inhibit both cancer cell growth and immune evasion in endometrial cancer.

Accumulating evidence suggests that ARID1A regulates tumor metabolism in a context-dependent manner. Previous studies have linked ARID1A loss to altered glutamine metabolism via upregulation of GLS1 [46], impaired cystine uptake and glutathione synthesis through dysregulation of SLC7A11 [47], and modulation of the mevalonate pathway to suppress pyroptosis in ovarian clear cell carcinoma [48]. Together, these findings support a broader role for ARID1A in shaping tumor metabolic states. Our study extends this concept by identifying polyamine biosynthesis as a previously unrecognized metabolic program controlled by ARID1A in EC. Using integrated transcriptomic and metabolomic analyses, we demonstrate that ARID1A loss activates YAP-dependent transcription of polyamine metabolic enzymes, resulting in robust accumulation of intracellular polyamines (Fig. 8). These findings reveal a novel mechanism by which ARID1A deficiency rewires EC metabolism.

Mechanistically, we uncover a direct regulatory interaction between ARID1A and the Hippo pathway effector YAP. YAP functions as a transcriptional coactivator through binding to TEAD family transcription factors and contains two WW domains that recognize proline-rich motifs in interacting proteins [49, 50]. We show that ARID1A directly binds YAP via conserved PPxY motifs, thereby preventing YAP–TEAD complex formation and restraining YAP-driven transcription. Loss of ARID1A disrupts this inhibitory interaction, shifting YAP toward TEAD binding and enhancing transcriptional output. This mode of regulation is distinct from canonical chromatin remodeling functions of ARID1A and highlights a nonchromatin mechanism by which ARID1A suppresses oncogenic signaling.

Polyamine metabolism has long been linked to oncogenic drivers, most notably MYC, which transcriptionally activates ODC1 to sustain elevated polyamine levels required for cell proliferation [51]. Our findings place YAP alongside MYC as a direct transcriptional regulator of ODC1 and other polyamine biosynthetic enzymes [52], providing a mechanistic explanation for the widespread polyamine accumulation observed across diverse cancer types. By linking ARID1A loss to YAP-mediated control of polyamine biosynthesis, this study bridges tumor suppressor dysfunction with metabolic dependency.

Beyond polyamine accumulation per se, our work elucidates how polyamines drive malignant phenotypes through selective translational control. Spermidine serves as an essential substrate for hypusination of eIF5A, a unique post-translational modification required for efficient translation elongation and termination at ribosome-stalling sequences [37, 39, 53]. We demonstrate that spermidine-induced eIF5A hypusination selectively enhances translation of LSD1, a chromatin-modifying enzyme that sustains oncogenic transcriptional programs downstream of YAP/TAZ [30]. Thus, polyamines act not merely as permissive growth factors but as active regulators of translational specificity, linking metabolic rewiring to epigenetic and transcriptional control.

Importantly, polyamine accumulation also exerts profound effects on the tumor-immune microenvironment. Polyamines have been reported to suppress immune activation and promote immune evasion [22, 54, 55]. Consistent with these observations, we show that ARID1A loss–driven polyamine accumulation impairs CD8⁺ T-cell recruitment, activation, and cytotoxic effector function in vivo. Inhibition of polyamine biosynthesis restored CD8⁺ T-cell activity and antitumor immunity, highlighting a dual role for polyamines in promoting tumor cell–intrinsic growth and suppressing antitumor immune responses.

These findings have direct therapeutic implications. Polyamine metabolism has emerged as an attractive target in oncology, given the heightened dependency of cancer cells on sustained polyamine synthesis [56, 57]. DFMO, an irreversible inhibitor of ODC1, has a long clinical history and a favorable safety profile [58–62], culminating in its recent FDA approval as maintenance therapy for high-risk neuroblastoma. Clinical studies in other malignancies underscore the importance of biomarker-based patient selection [63–65]. Our results suggest that ARID1A deficiency defines a polyamine-addicted subset of EC, in which DFMO effectively suppresses tumor growth while simultaneously restoring antitumor immunity. These observations provide a strong rationale for biomarker-enriched clinical trials evaluating DFMO alone or in combination with polyamine transport inhibitors or immune checkpoint blockade in ARID1A-mutant EC.

Several limitations should be acknowledged. Due to the lack of well-characterized murine EC cell lines (e.g., MecPK [66, 67], MSH2-369 [68]) suitable for immunocompetent models, we employed the syngeneic CT26 system to study immune interactions. Although not histologically representative of EC, this model enables interrogation of tumor-immune dynamics in vivo. In addition, our in vitro analyses were limited to two EC cell lines representing distinct molecular backgrounds. Future studies incorporating additional EC subtypes, patient-derived organoids, and conditional endometrial-specific ARID1A knockout mouse models will be essential to validate and extend these findings. Moreover, further studies are required to elucidate the functional role of LSD1 in ARID1A-deficient contexts and to explore the potential impact of ARID1A loss on transcriptional regulators beyond YAP.

In conclusion, this study identifies a previously unappreciated ARID1A–YAP–polyamine–eIF5A–LSD1 axis that integrates metabolic reprogramming, translational control, and immune evasion in endometrial cancer. By demonstrating that targeting polyamine biosynthesis suppresses tumor growth while restoring antitumor immunity, our findings highlight polyamine metabolism as a promising therapeutic vulnerability in ARID1A-deficient EC.

## Conclusions

Wild-type ARID1A prevents YAP activation and polyamine biosynthesis. ARID1A mutations in endometrial cancers lead to YAP hyperactivation, increasing polyamine production and promoting cancer cell growth. Spermidine enhances eIF5A hypusination, boosting LSD1 translation and malignancy. Polyamine accumulation also inhibits CD8^+^ T cell activation, aiding immune evasion. Targeting polyamine synthesis curbs cancer cell growth and immune evasion in ARID1A-mutated ECs.

## Materials and methods

### Cell lines and cell culture

HEK293T, human EC cell lines (Ishikawa, KLE, AN3CA, SPEC-2, HEC-1A, and HEC-1B), and CT26 cell line were acquired from the American Type Culture Collection and Fudan University. The HEK293T and KLE cells were grown in DMEM medium (BasalMedia, L110KJ) with the addition of 10% fetal bovine serum (FBS). Ishikawa, AN3CA, SPEC-2, HEC-1A, and HEC-1B cells were grown in DMEM/F12 medium (BasalMedia, L320KJ) with the addition of 10% FBS. CT26 cells were cultured in 1640 medium (BasalMedia, L210KJ) supplemented with 10% FBS. The cells were kept at a temperature of 37 °C in a humid incubator containing 5% CO_2_. To pack lentivirus, the HEK293T cell line was cultured in DMEM supplemented with 10% FBS at a temperature of 37 °C in the presence of 5% CO_2_. Each cell line was cultured for no more than 2 months and underwent authentication and testing for mycoplasma contamination.

### Mouse studies

The study involved the use of 4-week-old female BALB/c nude mice, 4-week-old female BALB/c mice, and 4-week-old female huPBMC-NOG mice. All mice were purchased from Beijing Vital River Laboratory Animal Technologies. The mice were kept in conditions that were free of specific pathogens (SPF) and followed a cycle of 12 h of light and 12 h of darkness. Illumination was maintained at 150–300 lux, with ambient temperature and humidity controlled at 20–26 °C and 40–70%, respectively. The cages were ventilated four times per hour to ensure proper air circulation. The mice were assigned to various groups based on their body weight and given a standard chow diet in a random manner. All animal experiments conducted in this study were approved by the Institutional Animal Care and Use Committee (IACUC) at Tongji University (No. TJBG10022102).

### CRISPR/Cas9-mediated Gene KO

To clone guide oligos targeting the ARID1A gene, we utilized the pX459 plasmid. The cells were transfected with pX459 constructs and then plated overnight. Following transfection, the cells were screened for 3 days using a concentration of 2 µg/mL puromycin (MedChemExpress). To obtain monoclonal cell lines, viable cells were placed in a 96-well plate using a restricted dilution method. The KO cell clones underwent screening through WB analysis and validation through Sanger sequencing. Supplementary Table S3 includes the gene-specific sgRNA sequences.

### CRISPR/Cas9-mediated Gene KI

The ARID1A KI cell lines were created by following a previously described protocol. To design a sgRNA that targeted the genomic sequence near the mutation site, the CRISPR design tool available at http://crispr.mit.edu was utilized. Afterwards, the sgRNA was inserted into pX459 constructs. A donor sequence with the ARID1A-Y148A or Y915A mutation was cloned into pDor04 constructs. At a ratio of 1:1, the two structures were simultaneously transfected into Ishikawa cells. Following a 24-h period, the cells were screened using puromycin (MedChemExpress) for a duration of 3 days. The cells that were positive for GFP were then enriched through FACS sorting and seeded into a 96-well plate with one cell per well. Following a period of around 14 days, the genetic material of every single clone was isolated and utilized as a blueprint for amplifying the DNA segment that encompassed the site of mutation. In the end, the existence of knock-in positive cells was confirmed through the utilization of Sanger sequencing.

### Western blotting

Cell lysates or immunoprecipitates were first separated by SDS-PAGE (Beyotime) and then transferred to PVDF membranes (Millipore). To prevent nonspecific binding of antibodies, the membranes were blocked in a blocking buffer for 30 min. Next, the dilution buffer was supplemented with primary antibodies and left to incubate with the membranes overnight at a temperature of 4 °C. After that, secondary antibodies were added to the membranes. In the end, protein bands were observed by utilizing an ECL kit (Tanon) and a chemiluminescent gel imaging system (Tanon). The antibodies utilized in this test are enumerated in the Supplementary Table S1 that presents the essential resources.

### Statistical analysis

For this study, SPSS Statistics (version 19.0; SPSS) and GraphPad Prism 8.0 (GraphPad Software) were employed for data analysis. The collection of data from human EC tissues, animal studies, and biological samples was done in a way that ensured blinding. The sample size was determined empirically based on previous studies. All collected data were included in the final statistical analysis. The mean ± SD results are presented based on at least three experiments. Statistical analyses were performed utilizing the One-way ANOVA test, the Two-way ANOVA test, or the Pearson Chi-square test. In this research, the levels of significance were determined as *P* < 0.05 denoting significance, *P* < 0.01 denoting high significance, and *P* < 0.001 denoting very high significance.

### Ethics approval and consent to participate

All methods were performed in accordance with the relevant guidelines and regulations. For studies involving human participants, the research investigations abided by the principles specified in the Declaration of Helsinki and were approved by the Ethics Committee of Shanghai First Maternity and Infant Hospital (No. KS2281). Informed consent was obtained from all participants.

For animal experiments, all protocols were approved by the Ethics Review Committee for Animal Experimentation at Tongji University (No. TJBG10022102).

## Supplementary information

ARID1A-Supplementary Methods and Figures

ARID1A-Supplementary Tables

Original WB data

## Data Availability

Detailed information on the reagents, antibodies, and the sequences of sgRNAs and shRNAs used in this study is provided in Supplementary Tables 1–3. All data generated or analyzed during this study are included in this published article and its supplementary information files. The high-throughput sequencing data generated in this study have been deposited in the NCBI Sequence Read Archive under the accession number PRJNA1276442. Other original data are available from the corresponding author upon reasonable request (wanxiaoping@tongji.edu.cn).
