# Supplementary material for: ARID1A deficiency-driven reprogramming of polyamine metabolism promotes endometrial cancer malignancy and immune escape
Source: Cell Death Dis. 2026 Apr 8;17(1):484. doi: 10.1038/s41419-026-08722-0 (PMC13186965; doi:10.1038/s41419-026-08722-0)
Supplement: Supplementary file 1 — ARID1A-Supplementary Methods and Figures [file 41419_2026_8722_MOESM1_ESM.docx]

Supplementary Materials for

**ARID1A Deficiency-Driven Reprogramming of Polyamine Metabolism Promotes Endometrial Cancer Malignancy and Immune Escape**

*Han Tao, Xiaojun Wang, Zhiyi Hu, Yiran Li, Mengwen Kong, Yeli Sun, Kun Gao*, Xiaoping Wan**

*Correspondence: kungao@tongji.edu.cn; wanxiaoping@tongji.edu.cn

**This PDF file includes:**

**Supplementary Materials and Methods**

**Supplementary Figures and Figure Legends**

**Other Supplementary Materials for this manuscript include the following:**

**Supplementary Table 1** Chemical reagents and antibodies.

**Supplementary Table 2** Primer sequence information.

**Supplementary Table 3** sgRNA/shRNA sequence information.

**Supplementary Table 4** ARID1A mutation status and IHC scores in 99 cases of endometrial cancer specimens and the associated clinical information.

**Supplementary Table 5** The differentially expressed genes in parental and ARID1A KO Ishikawa cells.

**Supplementary Table 6** The differential metabolites in parental and ARID1A KO Ishikawa cells.

**Supplementary Table 7** Plasmids used in this study.

**Supplementary Materials and Methods**

***RT-qPCR***

The TRIzol reagent (Sigma) was used to extract RNA samples from the designated cells, and the Evo M-MLV RT Kit with gDNA Clean (Agbio) was employed to perform cDNA synthesis according to the manufacturer’s instructions. RT-qPCR analysis was performed using the SYBR Green PCR kit (Agbio) with the Applied Biosystems QuantStudio 5 (Thermo Fisher Scientific). The 2^−ΔΔCt^ method was employed to assess mRNA expression levels, using β-Actin as the internal control. The primers utilized for this analysis can be found in the Supplementary Table S2.

***Cell Proliferation Assays***

Transfected cells were seeded in 96-well plates at a density of 3000 cells per well for the execution of the Cell Counting Kit-8 (CCK-8) assays. At designated time points (1, 2, 3, 4, and 5 days), 10 μl of CCK-8 solution was added to each well, followed by incubation of the plate at 37°C for a duration of 2 hours. Afterwards, the Multimode Plate Reader (Molecular Devices) was used to measure the absorbance at a wavelength of 450 nm for each well.

***Colony Formation Assays***

In the colony formation assays, the transfected cells were placed in each well of a 6-well plate at a density of 200 cells per well. They were then incubated in a medium volume of 2 mL for a duration of 10 days. To determine the presence of actively proliferating colonies, the cells in the wells were fixed with 4% paraformaldehyde and subsequently stained using crystal violet.

***EdU Assays***

For the EdU assays, the transfected cells were placed in a 96-well plate, containing 2,000 cells per well, and cultured for 2 days. Afterward, a 10 μM EdU concentration, acquired from Beyotime, was introduced into every well. The plate was then incubated at a temperature of 37°C for a duration of 2 hours. The cells were subsequently fixed, permeabilized, and treated with 400 μl of Hoechst 33342, which served to stain the nuclei. To determine the proportion of cells that were positive for EdU, the number of cells present in five randomly selected areas of each well was counted.

***Cell Migration and Invasion Assays***

Following a 12-hour period of cell culturing in serum-free medium, trypsin was used to digest the cells. Afterward, the cells were placed in the upper compartment of a Transwell chamber (Corning) within 24-well plates with a cell density of 4000 cells per well. In the upper compartment, there was a volume of 200 μl of DMEM medium without serum, whereas the lower compartments were filled with 800 μl of DMEM containing 10% FBS. Following a 10-minute incubation in 4% paraformaldehyde at ambient temperature, the filters were subsequently treated with 0.5% crystal violet for a duration of 30 minutes. After a duration of 24 hours, the optical microscope was utilized to observe and capture images of the filters. For invasion assays, the upper chamber was coated with Matrigel (30 μg per well; Corning) in accordance with the instructions provided by the manufacturer.

***RNA-sequencing and data analysis***

Total RNA was extracted from both parental and ARID1A KO Ishikawa cells using TRIzol reagent (Invitrogen). RNA integrity was assessed via 0.8% agarose gel electrophoresis and spectrophotometry. Only RNA with a 260/280 absorbance ratio between 1.8 and 2.2 was used for library construction and sequencing. Library preparation was performed according to Illumina’s protocols. mRNA was reverse transcribed into cDNA using Oligo-dT primers (APExBIO, China). The second cDNA strand was synthesized by amplification, and the cDNA products were purified using the AMPure XP system (Beckman Coulter). PCR amplification was conducted to enrich library fragments, which were then size-selected (350-550 bp). Library quality was assessed with the Agilent 2100 Bioanalyzer (Agilent). Sequencing was performed on the Illumina NovaSeq 6000 platform, generating raw reads. To obtain clean reads, adapter sequences and low-quality bases were removed using TrimGalore and Cutadapt. Clean reads were aligned to the hg19 human genome using HISAT2. Gene expression was quantified through reference genome-guided transcriptome assembly with StringTie. Differentially expressed genes (DEGs) were identified using DESeq2 (for replicated samples), with a log2|fold-change| cutoff of ≥ 0.25 and p-value < 0.05. Functional enrichment of significant DEGs and genes within identified modules was performed using gene ontology (GO) and KEGG pathway analysis via ClusterProfiler, considering terms with p-values < 0.05 as significant. Additionally, gene set enrichment analysis (GSEA) was performed using the clusterProfiler package, ranking genes by log2 fold-change.

***Metabolomics Analysis***

The project included a total of 10 cell samples, which were analyzed using a UHPLC-HRMS/MS-based non-targeted metabolomics platform. The analysis process involved several steps, including sample preparation, UHPLC-HRMS/MS analysis, preprocessing of raw mass spectral data, statistical analysis (both univariate and multivariate), and identification of differential metabolites. To prepare the cell samples, they were subjected to 5 rounds of ultra-sonication with breaks in an ice-water bath, then vortexed and left at -20 °C for 30 minutes. Following centrifugation, the liquid above the sediment was removed by evaporation using nitrogen and then mixed with 10% methanol before undergoing UHPLC-HRMS/MS analysis. Pooling all the prepared samples resulted in a QC sample being obtained. The ThermoFisher Ultimate 3000 UHPLC system was utilized for chromatographic separation, employing a Waters ACQUITY UPLC BEH C18 column. In positive mode, the mobile phases included a mixture of water, methanol, and formic acid, while in negative mode, the mobile phases consisted of a combination of water, methanol, and water with ammonium bicarbonate. Both modes utilized a linear gradient elution. The eluents underwent analysis using a ThermoFisher Q Exactive™ Hybrid Quadrupole-Orbitrap™ Mass Spectrometry (QE) instrument with a flow rate of 0.25 mL/min.

***Chromatin Immunoprecipitation (ChIP) Assays and ChIP-seq***

Chromatin immunoprecipitation assays were performed using the Sonication ChIP Kit (RK20258, Abclonal) according to the manufacturer’s instructions. Briefly, cells were cross-linked with formaldehyde, collected by centrifugation, and resuspended in lysis buffer. Chromatin was fragmented by sonication, and insoluble debris was removed by centrifugation. A portion of the supernatant was saved as the input control. The remaining supernatants were incubated overnight at 4 °C with an anti-YAP antibody (#14074, Cell Signaling Technology) and protein A/G magnetic beads (RM02915, Abclonal). After extensive washing, the immunoprecipitated chromatin complexes were eluted, reverse cross-linked, and purified. The recovered DNA was quantified by qPCR or subjected to next-generation sequencing. For ChIP-seq analysis, sequencing libraries were constructed using the Rapid Plus DNA Lib Prep Kit for Illumina V2 (RK20255, Abclonal) and sequenced by Shanghai Personalbio Technology Co., Ltd.

***Polyamine Detection***

BioVision’s total polyamine assay kit (cat.no.K475) was utilized for the detection of overall polyamines. The test was performed in accordance with the guidelines provided by the manufacturer. For the measurement of individual polyamines, a metabolomics platform was used in combination with LC-MS after dansylation. The extraction process involved using 80% acetonitrile to extract the tissues and cultured cells. Subsequently, 100 μl of the resulting supernatant was dried using a SpeedVac. In order to regenerate the samples, 45 μl of water, 10 μl of 10× PBS, 5 μl of 10 μM polyamine internal standards, and 45 μl of 1 mg/ml dansyl chloride (Sigma) dissolved in acetonitrile were added to the same tube. After incubating the mixture at a temperature of 55 °C for a duration of 10 minutes, 400 μl of water was added. The samples were extracted using a 96-well plate called Oasis HLB, and then eluted with 98% acetonitrile. After elution, the samples were dried using a SpeedVac. In the end, the dehydrated samples were dissolved in 50 μl of water containing 0.1% formic acid. Then, 20 μl of the resulting solution was utilized for LC-MS analysis. After normalizing the polyamine concentrations to the internal standards and the total soluble protein content, the results were obtained.

***Cytoplasm and Nucleus Protein Fractionation***

To resuspend the cells, the cells were suspended in a lysis buffer containing 10 mM HEPES at pH 7.8, 0.34 M sucrose, 10% glycerol, 10 mM KCl, 1.5 mM MgCl_2_, 0.1% Triton X-100, 1mM PMSF, and protease inhibitors. Then, the suspension was centrifuged at 10,000 × g for 5 minutes, and the cytoplasm fraction was retained. The nuclear pellets were then lysed on ice for a duration of 30 minutes in the nuclear lysis buffer, which consisted of 50 mM Tris-HCl pH 7.8, 420 mM NaCl, 0.34 M sucrose, 0.5% Nonidet P-40, and protease inhibitors. To denature the proteins, a solution of SDS loading buffer was added and heated to 100 °C for a duration of 10 minutes.

***Co-immunoprecipitation (Co-IP) Assays***

Lysis buffer was employed to lyse the cells for a duration of 20 minutes at 4 °C. Subsequently, the cell lysates were supplemented with Protein A-agarose and left to incubate at a temperature of 4 °C for a duration of 2 hours. Afterward, antibodies were introduced and permitted to interact overnight at a temperature of 4 °C. The beads underwent a washing process with lysis buffer. The immunoprecipitates that were attached to the beads were extracted by utilizing 2×SDS loading buffer. The immunoprecipitates that were obtained were separated on gels made of 10% SDS-PAGE and then subjected to immunoblotting using the specified antibodies.

***Immunofluorescence Staining***

For a period of 48 hours, the designated plasmids were used to co-transfect the Ishikawa cells, which were grown on a 6-well plate. After placing the cells onto chamber slides, they were subsequently fixed at room temperature for 30 minutes using a 4% solution of paraformaldehyde. After rinsing with PBS, the cells were treated with 0.1% Triton X-100 in PBS at room temperature for 15 minutes to achieve permeabilization. Afterwards, the cells were blocked with 3% BSA at room temperature for 1 hour and then left to incubate overnight at 4°C with primary antibodies in PBS. Following a rinse with PBS, the cells underwent incubation with fluorescence-labeled secondary antibodies for 1 hour at room temperature in the dark. After the procedure was completed, the last task involved staining the nuclei of cells with DAPI at room temperature for 10 minutes, then washing and mounting the cells.

***GST-fusion Pull Down Assays***

The production of GST and GST-fusion/His fusion proteins was carried out, followed by the activation of these proteins using 0.1 mM isopropyl-β-d-thiogalactopyranoside overnight at 16 °C in Escherichia coli. The proteins were purified by utilizing glutathione–Sepharose 4B beads from GE Healthcare in the United States. In order to guarantee comparability, the same quantity of proteins was subsequently mixed with GST fusion proteins in TEN buffer (10 mM tris-HCl, pH 8.0, 1 mM EDTA, and 100 mM NaCl) at a temperature of 4 °C for a period of 4 hours. Before undergoing precipitation, the components were washed three times with TEN buffer in order to eliminate any lingering impurities. Finally, the analysis of the components was conducted using Western blotting.

***Bimolecular Fluorescence Complementation (BiFC)***

The YAP and ARID1A sequences, which cover the entire genes, were amplified and combined with the YFP protein at the N-terminal (pBiFC-VN173) and at the C-terminal (pBiFC-VC155), respectively. Ishikawa cells were grown in a 6-well dish, and then co-transfected with 2 μg of both pBiFC-VN173-YAP and pBiFC-VC155-ARID1A plasmids. The cells were fixed with a 4% paraformaldehyde solution and then exposed to 0.1% Triton X-100. Afterwards, the nuclei of the cells were dyed with DAPI solution at room temperature for a duration of 10 minutes. Following 48 hours, the emitted fluorescence from the yellow fluorescent protein was examined utilizing a confocal microscope.

***Luciferase Reporter Assays***

For promoter-firefly luciferase plasmid construction, a 2 kb DNA segment upstream of the transcription start site of *ODC1* was cloned into the pGL4.2 vector. In order to perform the transfection, a mixture of the luciferase reporter vector, pRL-TK vector, and additional plasmids were co-transfected into 293T cells, which had been previously plated in a 12-well dish. After incubating for 24 hours, the cells were harvested in lysis buffer. Luciferase activity was measured using the Dual-Luciferase Reporter Assay System (Promega) according to the manufacturer’s instructions.

***Lentivirus Packaging and Generation of Stable Cell Lines***

Lentiviral particles expressing shCtrl, shYAP, or shODC1 were produced using shRNA sequences synthesized by Genechem (Shanghai, China), with detailed sequences listed in Table S3. For the overexpression constructs, the pCDH-copGFP plasmid (System Biosciences, USA) and the pLVX-TetOne-Puro plasmid (Clontech, USA) were used to clone ARID1A-WT and its mutant variants. Target cells were transduced with lentiviral particles in the presence of 8 µg/ml polybrene (Sigma-Aldrich) to enhance infection efficiency. Following a 24-hour incubation, the viral supernatant was replaced with fresh medium, and cells were subjected to puromycin selection (2 µg/ml) for three consecutive days to establish stably transduced cell populations. For the generation of a doxycycline-inducible ARID1A re-expression system, the pLVX-TetOne lentiviral construct carrying the ARID1A coding sequence was introduced into ARID1A-knockout cells. The pLVX-TetOne vector is based on a Tet-On 3G system, in which the transcription of ARID1A is tightly regulated and can be activated upon administration of doxycycline. To induce ARID1A expression, cells were treated with doxycycline (100 ng/ml) for 24 hours unless otherwise specified. This system enabled precise and reversible control of ARID1A expression levels, allowing functional studies under both inducible and withdrawal conditions.

***Tumor Xenograft Model***

The Ethics Review Committee for Animal Experimentation at Tongji University ensured that all experimental protocols involving animals were conducted in compliance with ethical guidelines (No. TJBG10022102). Mice of the BALB/c nude strain, aged between 4 and 6 weeks, were obtained from Beijing Vital River Laboratory Animal Technology Co., Ltd. These mice were injected subcutaneously in the flank with 1 × 10^5^ cells in a 100 µl PBS solution using one of three Ishikawa cell types: parental cells, ARID1A KO cells, or ARID1A KO cells stably modified to express ARID1A-WT. For experiments using CT26 cells, BALB/c mice of the same age and from the same supplier were injected subcutaneously in the flank with 1 × 10^5^ parental or ARID1A KO CT26 cells in a 100 µl PBS solution. The size of the tumor was monitored at regular intervals of two days using a digital caliper to measure its length and width. The ellipsoid volume formula, V = (L × W^2^) / 2, was used to calculate the tumor volumes, with L representing the length and W representing the width. Mice were euthanized if either signs of ulceration in the tumor were detected or if the tumors reached the maximum permissible size. The weight of the tumors was also recorded during this time. Following the sacrifice of the mice, the tumor tissues were partitioned, with a section of the tissues subjected to formalin fixation and subsequently embedded in paraffin for immunofluorescence analysis.

***Cytokines Detection***

BALB/c mice were humanely euthanized, and their spleens along with peripheral lymph nodes (cervical, axillary, brachial, and inguinal) and mesenteric lymph nodes were harvested and pooled. These tissues were enzymatically digested with 500 µg/ml collagenase D (Roche) and 25 µg/ml DNase I (Sigma-Aldrich), and the resulting single-cell suspensions were prepared in PBS. The resulting cell suspension was washed twice with PBS and resuspended in complete RPMI medium. CD8^+^ T cells were isolated using a mouse CD8^+^ T cell isolation kit (BioLegend) according to the manufacturer’s protocol and used as effector cells. These CD8^+^ T cells were activated for 48 hours at 37°C using CD3/CD28 magnetic beads (Gibco) and IL-2 (Sigma-Aldrich) in complete RPMI medium. The activated CD8^+^ T cells were then co-cultured with either parental CT26 cells or ARID1A KO CT26 cells, which had been pre-treated with either DMSO (vehicle control) or DFMO, at an effector-to-target ratio of 2:1 for 24 hours. Following co-culture, supernatants were collected and analyzed for cytokine production using mouse IFN-γ and TNF-α ELISA kits (Thermo Fisher Scientific) following the manufacturer’s instructions. Absorbance was measured at 450 nm, with 540 nm set as the correction wavelength, using a microplate reader (Molecular Devices) to quantify cytokine levels.

***FACS Staining and Analysis***

Human peripheral blood mononuclear cells (PBMCs) were isolated from whole blood via density-gradient centrifugation using Ficoll–Paque. CD8^+^ T cells were purified immediately after isolation using a magnetic bead-based negative selection kit (11348D, Invitrogen) according to the manufacturer’s instructions to ensure high purity and viability. Purified CD8⁺ T cells were then activated by culturing in plates pre-coated with anti-CD3 (5 μg/mL; Clone OKT3, BioLegend) and soluble anti-CD28 (1 μg/mL; BioLegend) in RPMI-1640 supplemented with 10% FBS and IL-2 (10 ng/mL) for 48 h. Activated CD8⁺ T cells were harvested, washed, and labeled with 2.5 μM CFSE (E-CK-A345, Elabscience) in pre-warmed PBS for 10 min at 37 °C. Labeling was quenched by adding 5 volumes of complete medium. Ishikawa cells were seeded 24 h prior to co-culture. Labeled CD8⁺ T cells were then co-cultured with Ishikawa cells at an effector-to-target (E:T) ratio of 2:1 in the presence of DFMO or vehicle control for 48 h. For cytokine detection, non-adherent T cells were harvested from the co-culture and re-stimulated in a new plate coated with anti-CD3 (5 μg/mL) and soluble anti-CD28 (1 μg/mL) for 4 h in the presence of Brefeldin A (Protein Transport Inhibitor, BioLegend). Cells were washed and first stained with a Fixable Viability Dye (eBioscience) to exclude dead cells. Surface staining was performed at 4 °C for 30 min using fluorochrome-conjugated antibodies against CD8 (E-AB-F1110C, Elabscience), CD69 (E-AB-F1138C, Elabscience), PD-1 (E-AB-F1229E, Elabscience), and TIM-3 (11-3109-42, Invitrogen). Cells were then fixed and permeabilized using the Cytofix/Cytoperm Kit (BD Biosciences), followed by intracellular staining with anti–IFN-γ (E-AB-F1196E, Elabscience) and anti–TNF-α (562082, BD Biosciences). Data were acquired on a BD FACSCanto™ II flow cytometer and analyzed using FlowJo software. The gating strategy excluded debris, doublets, and dead cells. Proliferation was quantified by CFSE dilution, and phenotype/cytokine expression was analyzed within the viable CD8⁺ T cell population.

***Immunohistochemistry (IHC) Staining***

Samples of EC from Shanghai First Maternity and Infant Hospital in Shanghai, China, were gathered between January 2010 and January 2022, comprising tissue specimens (Table S4). Every patient enrolled in the research was diagnosed with endometrial carcinoma and received histological verification via tissue biopsies. No patients had previously undergone radiotherapy, endocrine therapy, chemotherapy, or surgery. The research investigations abided by the principles specified in the Declaration of Helsinki by the World Medical Association and adhered to ethical guidelines. Consent was acquired from every participant or their legal guardian following approval from the hospital ethics committee (No. KS2281). Histopathological examination involved hematoxylin and eosin staining, as well as immunohistochemistry (IHC), following the methods described in our published study. The IHC examination employed a particular group of antibodies documented in Table S1. Three independent pathologists assessed the intensity of staining using a scale that ranged from 0 (absence of staining) to 3 (intense staining). Additionally, the proportion of cells showing staining was assessed on a scale of 0% to 100%.

**Supplementary Figure and Figure Legends**


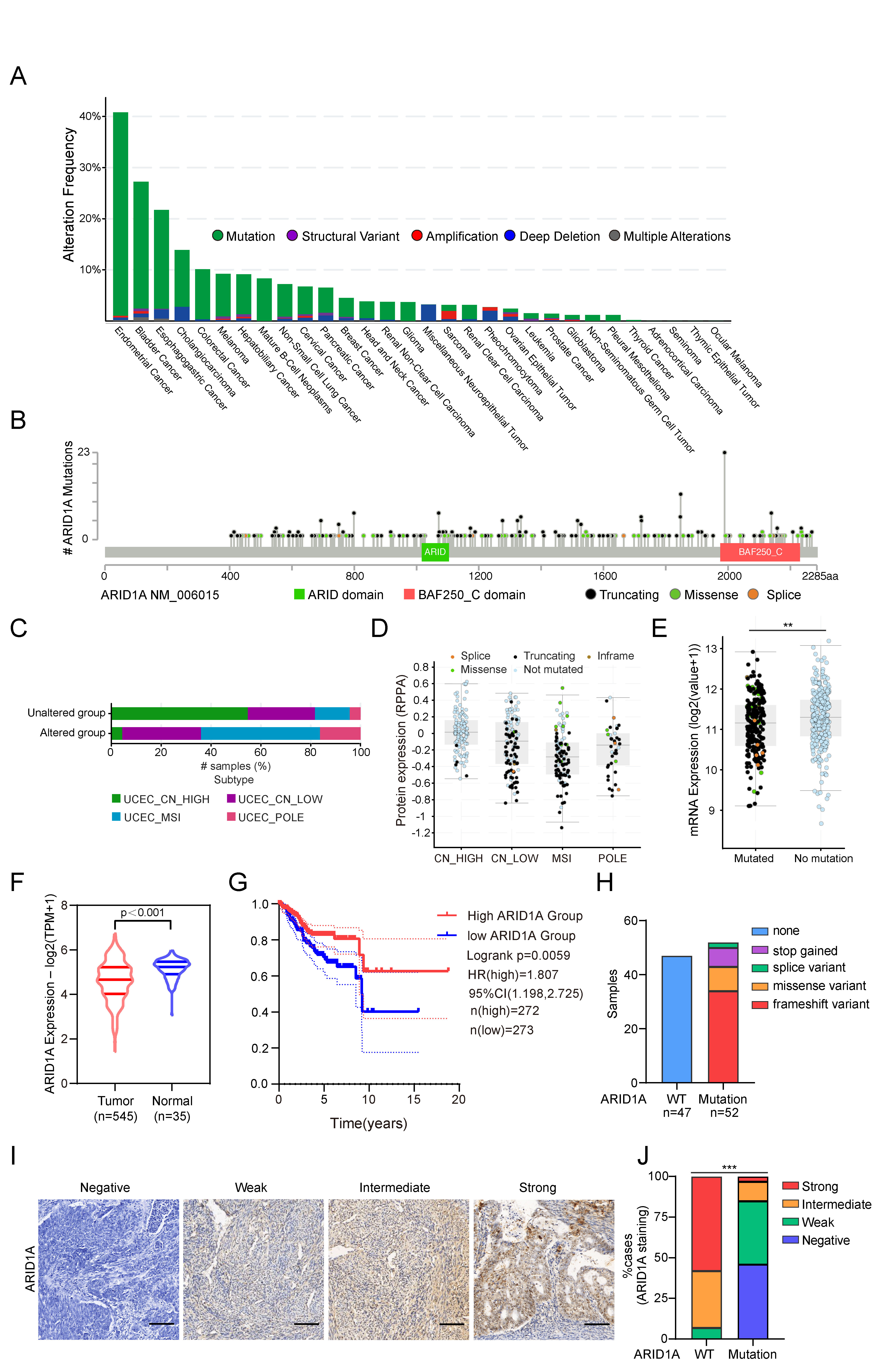


**Figure S1. ARID1A is frequently mutated and downregulated in endometrial cancer.**

**A** The mutation features of ARID1A were analyzed in various tumors from the TCGA dataset using the cBioPortal tool.

**B** The schematic of the ARID1A protein illustrates the specific locations of different somatic mutations identified in the endometrial cancer TCGA cohort. The green square represents the ARID domain of the ARID1A protein, and the red square represents the BAF250_C domain. Black dots: Truncating mutations; Green dots: Missense mutations; Yellow dots: Splice mutations.

**C** The distribution of the four types of endometrial cancer in ARID1A-WT and ARID1A-mutated EC specimens.

**D** Protein expression of ARID1A in the four types of EC.

**E** The mRNA expression levels of ARID1A were compared between ARID1A-WT and ARID1A-mutated EC specimens.

**F** The expression of ARID1A mRNA was assessed in both normal endometrial tissues and EC tissues obtained from the TCGA cohort.

**G** Kaplan–Meier survival analysis of EC patients stratified by high and low ARID1A mRNA expression in the TCGA cohort. Patients were divided into high- and low-expression groups based on the median ARID1A mRNA expression level. Solid lines indicate estimated survival probabilities, and dotted lines represent 95% confidence intervals (CIs). The log-rank test was used to assess statistical significance (p = 0.0059). The hazard ratio (HR) for the high-expression group relative to the low-expression group was 1.807 (95% CI, 1.198–2.725).

**H** Mutation screening was performed to identify ARID1A mutations in EC specimens.

**I, J** Representative IHC images of ARID1A staining in 99 EC patient specimens, including 47 ARID1A-WT and 52 ARID1A mutant (MUT) cases, along with the quantitative data of ARID1A staining. Scale bar, 50 μm. *P* values are calculated using One-way ANOVA test in (**E, F**) and Two-way ANOVA test in (**J**). **p* < 0.05, ***p* < 0.01, ****p* < 0.001, n.s., not significant.


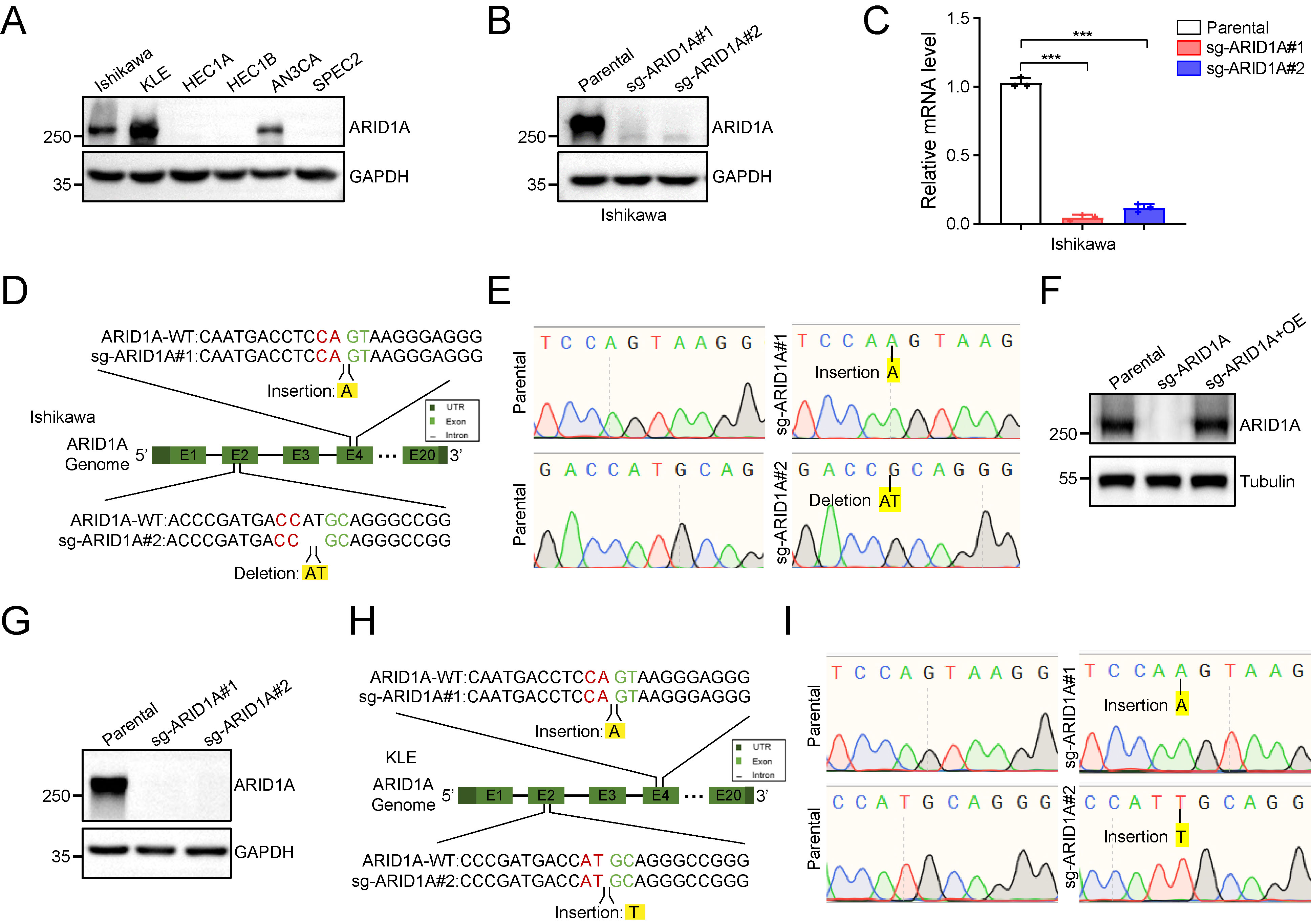


**Figure S2. Validation of ARID1A knockout and overexpression in endometrial cancer cells (related to Figure 1).**

**A** Western blot analysis of the indicated proteins in whole cell lysates (WCLs) from six EC cell lines.

**B** Western blot analysis of the indicated proteins in WCLs from two independent ARID1A KO Ishikawa cell clones generated using CRISPR/Cas9 methods. Parental Ishikawa cells were used as a control.

**C** RT-qPCR assessment of ARID1A mRNA expression in parental and ARID1A KO Ishikawa cells. GAPDH mRNA levels were used for normalization. Data are shown as means ± SD (n=3).

**D** Schematic of CRISPR/Cas9-mediated KO of ARID1A by sgRNA#1 or sgRNA#2 in Ishikawa cells.

**E** Sanger sequencing confirming that the ARID1A gene was edited by sgRNA#1 or sgRNA#2 in Ishikawa KO cells.

**F** Western blot analysis of the indicated proteins in WCLs from Ishikawa cells of the Parental, sg-ARID1A, and sg-ARID1A+OE groups.

**G** Western blot analysis of the indicated proteins in WCLs from two independent ARID1A KO KLE cell clones generated using CRISPR/Cas9 methods. Parental KLE cells were used as a control.

**H** Schematic of CRISPR/Cas9-mediated KO of ARID1A by sgRNA#1 or sgRNA#2 in KLE cells.

**I** Sanger sequencing confirming that the ARID1A gene was edited by sgRNA#1 or sgRNA#2 in KLE KO cells. *P* values are calculated using One-way ANOVA test in (**C**). **p* < 0.05, ***p* < 0.01, ****p* < 0.001, n.s., not significant.


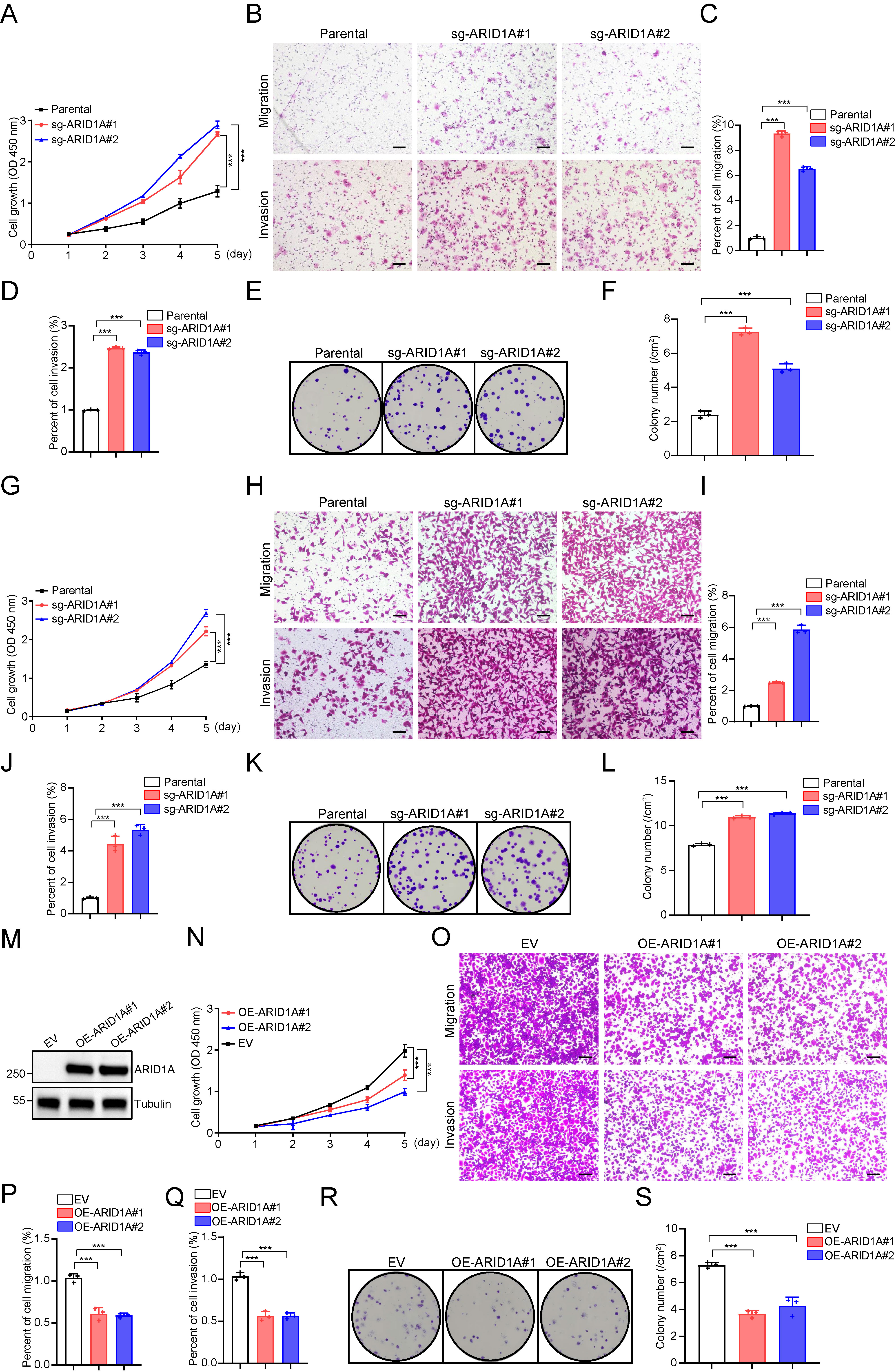


**Figure S3. ARID1A suppresses malignant phenotypes of EC cells** **(related to Figure 1).**

**A** CCK-8 assays were performed in parental Ishikawa cells and ARID1A KO cells for the specified duration. Data are presented as mean ± SD (n = 3 independent biological experiments).

**B-D** Transwell migration and invasion assays were performed in parental and ARID1A KO Ishikawa cells. Data are presented as mean ± SD (n = 3 independent biological experiments). Scale bar, 100 μm.

**E, F** Colony formation assays were performed in parental and ARID1A KO Ishikawa cells. Data are presented as mean ± SD (n = 3 independent biological experiments).

**G** CCK-8 assays were performed in parental KLE cells and ARID1A KO cells for the specified duration. Data are presented as mean ± SD (n = 3 independent biological experiments).

**H-J** Transwell migration and invasion assays were performed in parental and ARID1A KO KLE cells. Data are presented as mean ± SD (n = 3 independent biological experiments). Scale bar, 100 μm.

**K, L** Colony formation assays were performed in parental and ARID1A KO KLE cells. Data are presented as mean ± SD (n = 3 independent biological experiments).

**M** Western blot analysis of the indicated proteins in WCLs from control and ARID1A overexpression HEC-1B cells.

**N** CCK-8 assays were performed in control and ARID1A overexpression HEC-1B cells for the specified duration. Data are presented as mean ± SD (n = 3 independent biological experiments).

**O-Q** Transwell migration and invasion assays were performed in control and ARID1A overexpression HEC-1B cells. Data are presented as mean ± SD (n = 3 independent biological experiments). Scale bar, 100 μm.

**R, S** Colony formation assays were performed in control and ARID1A overexpression HEC-1B cells. The quantitative data are presented in **S**. Data are presented as mean ± SD (n = 3 independent biological experiments). *P* values are calculated using One-way ANOVA test in (**C, D, F, I, J, L, P, Q, S**) and Two-way ANOVA test in (**A, G, N**). *p<0.05, **p<0.01, ***p<0.001, n.s., not significant.


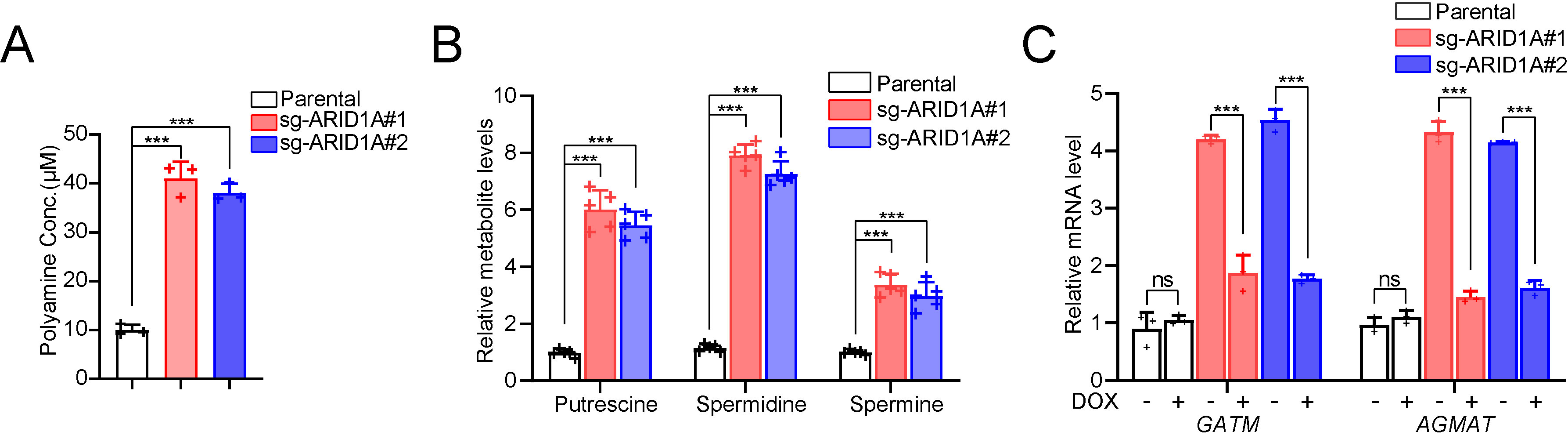


**Figure S4. ARID1A KO leads to aberrant upregulation of polyamine biosynthesis (related to Figure 2).**

**A** Total polyamine levels were measured in parental and ARID1A KO Ishikawa cells. Data are presented as mean ± SD (n = 3 independent biological experiments).

**B** Relative levels of specific polyamines. Data are presented as mean ± SD (n = 5 independent biological experiments).

**C** The mRNA expression of polyamine metabolic genes was measured using RT-qPCR in parental and ARID1A-tet-on KO Ishikawa cells treated with either DMSO or DOX (100 ng/ml) for 24 hours. Data are presented as mean ± SD (n = 3 independent biological experiments). *P* values are calculated using One-way ANOVA test in (**A, B, C**). **p* < 0.05, ***p* < 0.01, ****p* < 0.001, n.s., not significant.


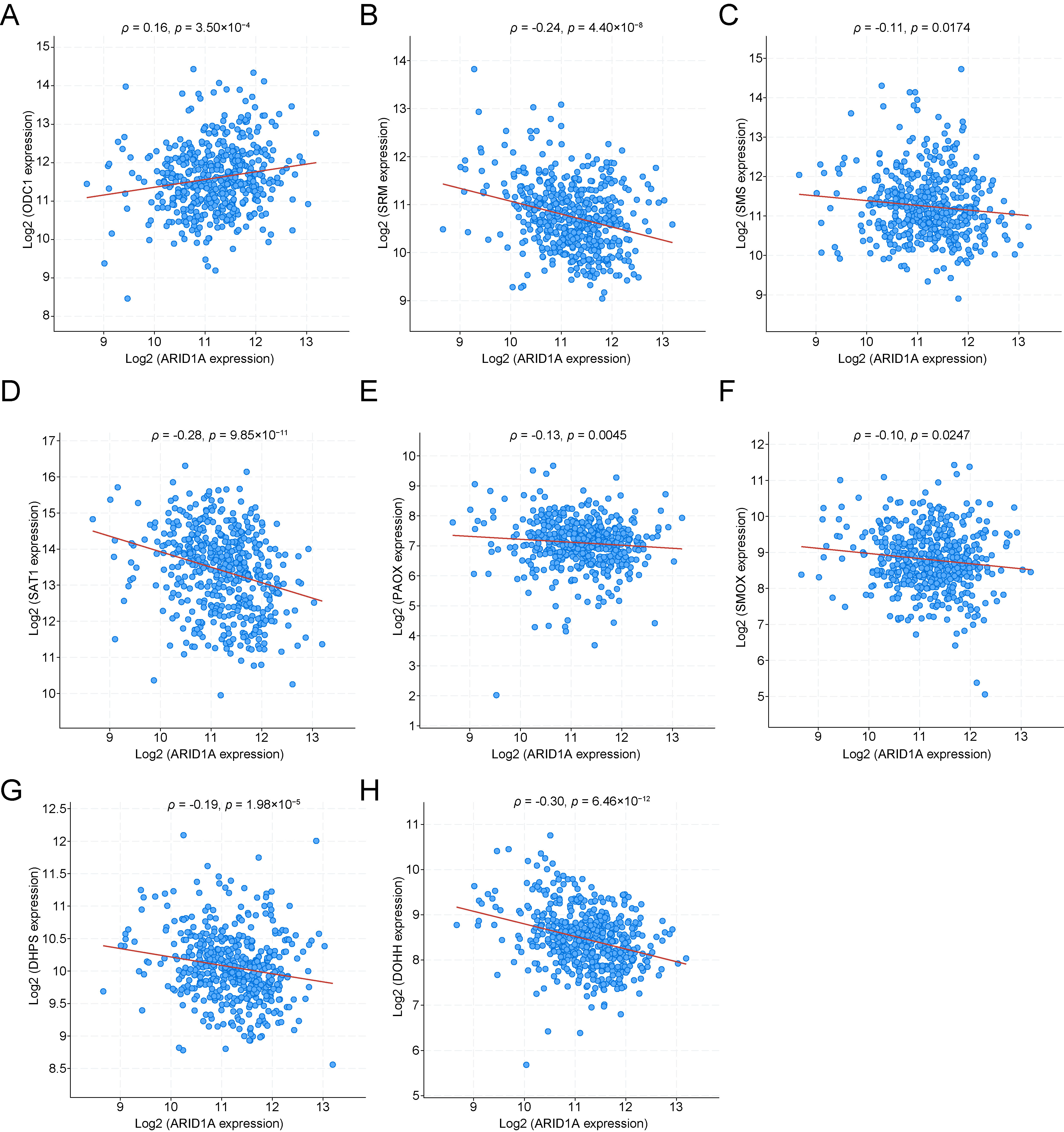


**Figure S5. Correlation analysis of ARID1A expression with polyamine metabolic enzymes in EC from the TCGA database (related to Figure 2).**

**A** Spearman correlation analysis of ARID1A and ODC1 expression in tumor tissues from EC patients in the TCGA dataset. Each point represents a sample; the X- and Y-axes indicate gene expression levels (Spearman correlation coefficient ρ = 0.16, p = 3.50 × 10^-4^).

**B** Spearman correlation analysis of ARID1A and SRM expression in tumor tissues from EC patients in the TCGA dataset (ρ = -0.24, p = 4.40 × 10^-8^).

**C** Spearman correlation analysis of ARID1A and SMS expression in tumor tissues from EC patients in the TCGA dataset (ρ = -0.11, p = 0.0174).

**D** Spearman correlation analysis of ARID1A and SAT1 expression in tumor tissues from EC patients in the TCGA dataset (ρ = -0.28, p = 9.85 × 10^-11^).

**E** Spearman correlation analysis of ARID1A and PAOX expression in tumor tissues from EC patients in the TCGA dataset (ρ = 0.13, p = 0.0045).

**F** Spearman correlation analysis of ARID1A and SMOX expression in tumor tissues from EC patients in the TCGA dataset (ρ = -0.10, p = 0.0247).

**G** Spearman correlation analysis of ARID1A and DHPS expression in tumor tissues from EC patients in the TCGA dataset (ρ = -0.19, p = 1.98 × 10^-5^).

**H** Spearman correlation analysis of ARID1A and DOHH expression in tumor tissues from EC patients in the TCGA dataset (ρ = -0.30, p = 6.46 × 10^-12^).


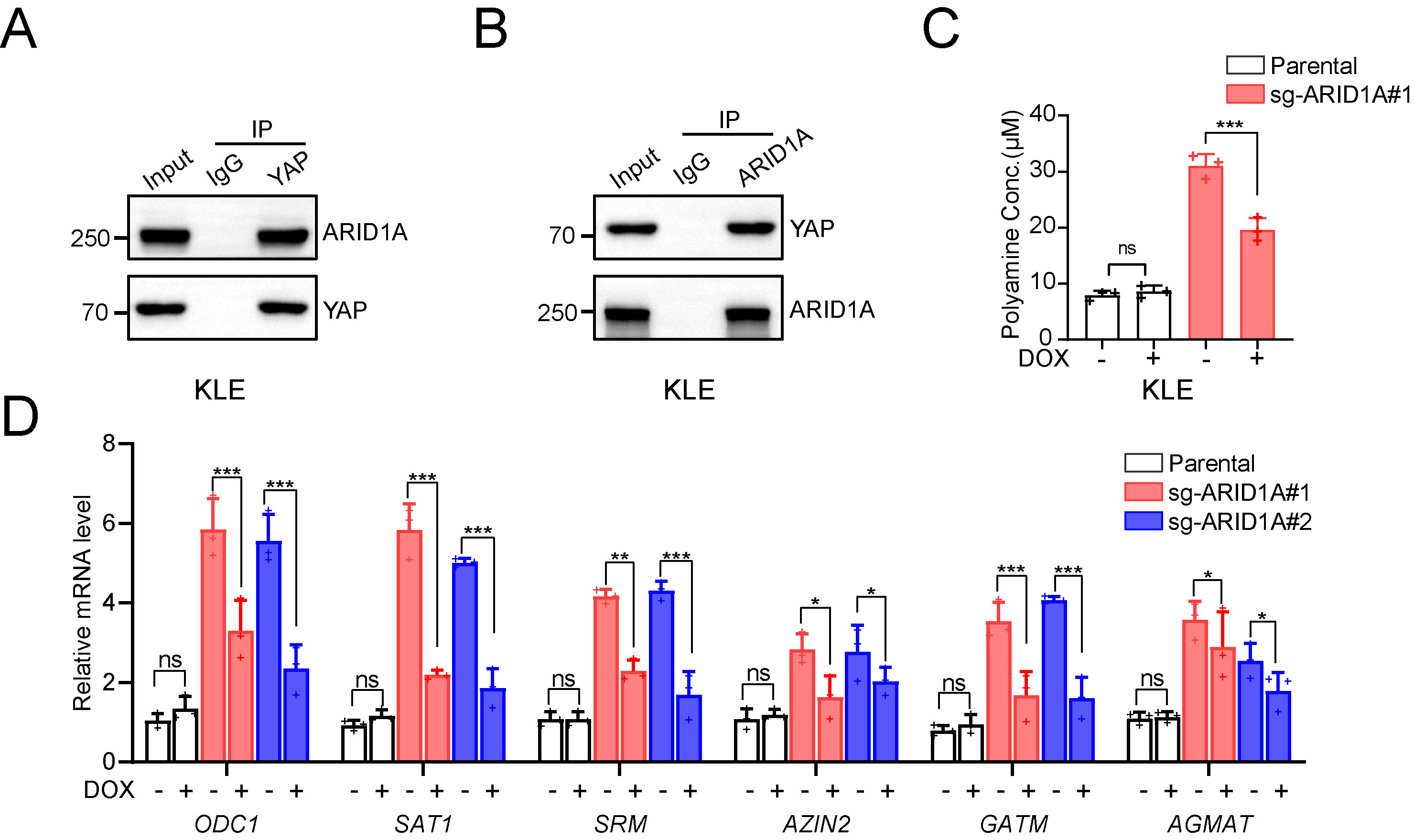


**Figure S6. Validation of the ARID1A–YAP–polyamines axis in KLE cells (related to Figure 3).**

**A** Western blot analysis was performed on WCLs and Co-IP samples derived from cell extracts of KLE cells, using IgG or anti-YAP antibody.

**B** Western blot analysis was performed on WCLs and Co-IP samples derived from cell extracts of KLE cells, using IgG or anti-ARID1A antibody.

**C** Total polyamine levels were measured in parental and ARID1A-tet-on KO KLE cells after treatment with either DMSO or DOX (100 ng/ml) for 24 hours. Data are presented as mean ± SD (n = 3 independent biological experiments).

**D** The mRNA expression of polyamine metabolic genes was measured using RT-qPCR in parental and ARID1A-tet-on KO KLE cells treated with either DMSO or DOX (100 ng/ml) for 24 hours. Data are presented as mean ± SD (n = 3 independent biological experiments). *P* values are calculated using One-way ANOVA test in (**C, D**). **p* < 0.05, ***p* < 0.01, ****p* < 0.001, n.s., not significant.


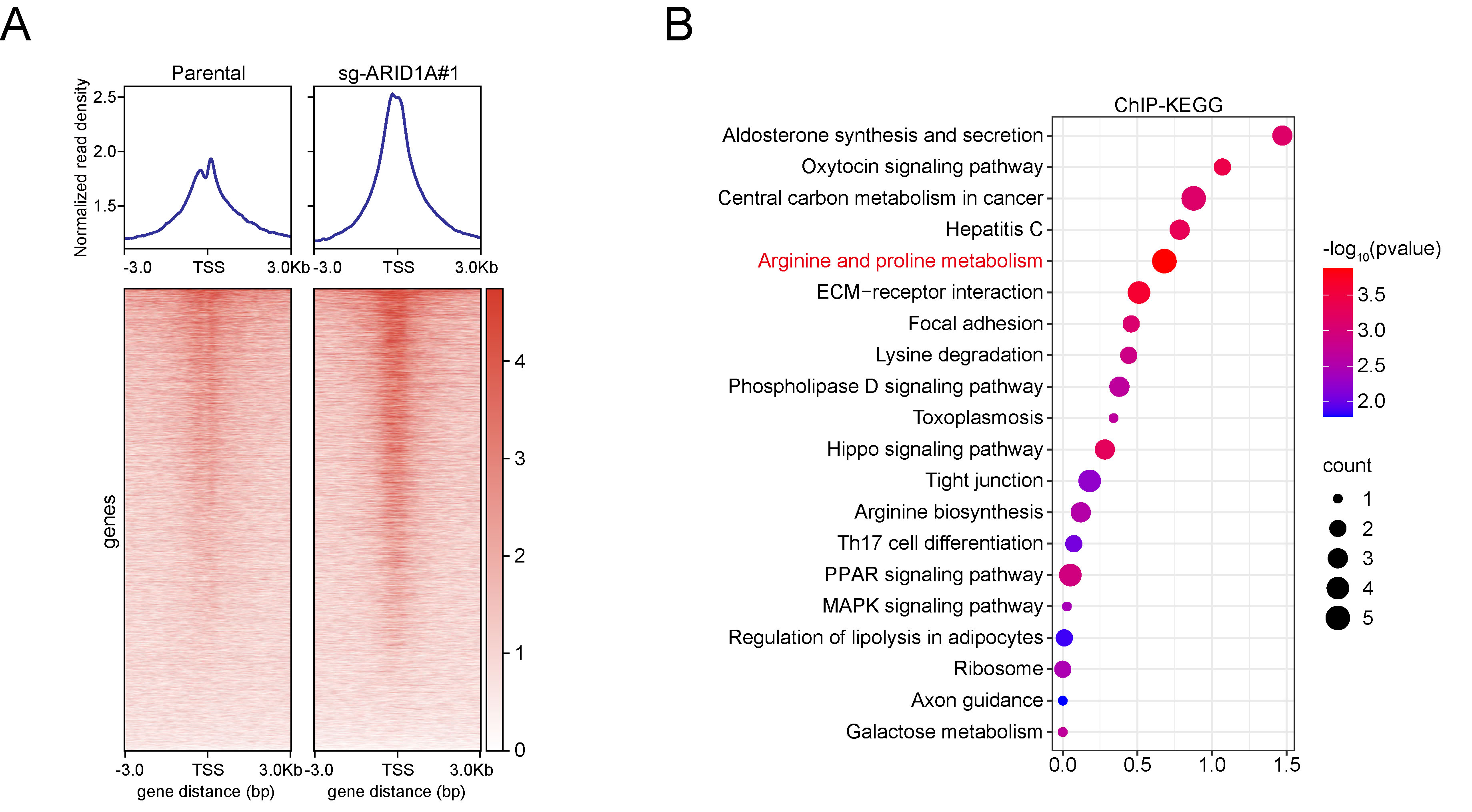


**Figure S7. YAP contributes to ARID1A KO-induced polyamine metabolism (related to Figure 4).**

**A** ChIP-seq analysis of YAP-binding intensities in Ishikawa cells with and without ARID1A knockout. Chromatin immunoprecipitation against YAP followed by next-generation sequencing was performed; bp, base pair.

**B** KEGG pathway enrichment analysis showing that the polyamine biosynthesis pathway is upregulated in ARID1A KO cells.


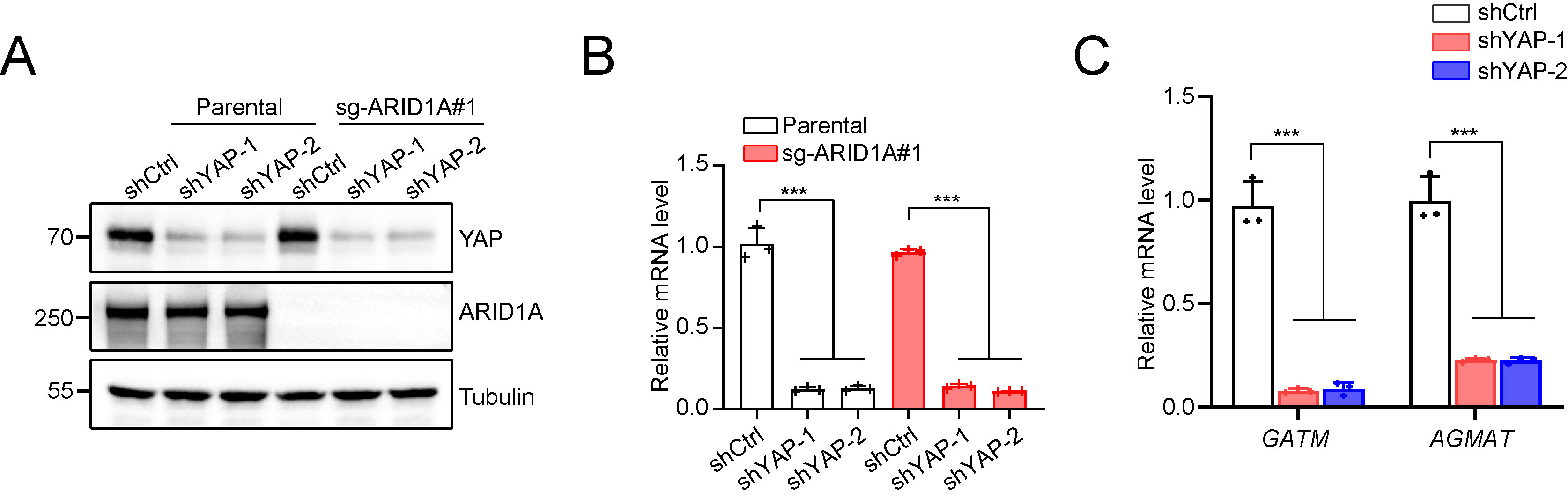


**Figure S8. YAP contributes to ARID1A KO-induced polyamine accumulation and malignant progression (related to Figure 4).**

**A** Western blot analysis was performed to examine the specified proteins in Ishikawa cells infected with lentivirus expressing shctrl, shYAP-1, or shYAP-2, including both parental and ARID1A KO cells.

**B** RT-qPCR was used to measure the levels of YAP mRNA. Data are presented as mean ± SD (n = 3 independent biological experiments).

**C** The mRNA expression of polyamine metabolic genes was measured using RT-qPCR in ARID1A KO Ishikawa cells infected with lentivirus expressing shctrl, shYAP-1, or shYAP-2. Data are presented as mean ± SD (n = 3 independent biological experiments). *P* values are calculated using One-way ANOVA test in (**B, C**). **p* < 0.05, ***p* < 0.01, ****p* < 0.001, n.s., not significant.


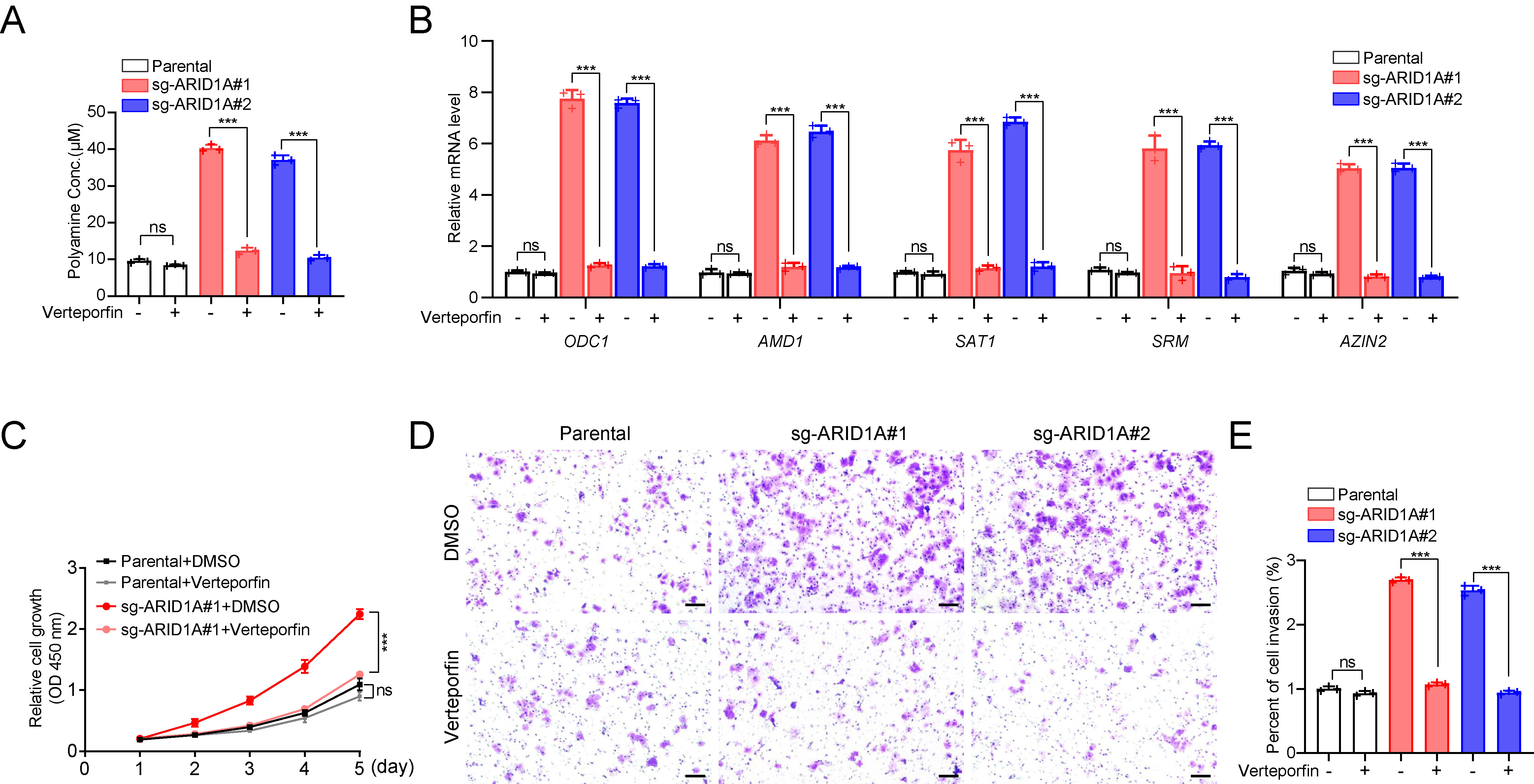


**Figure S9. Verteporfin inhibits ARID1A KO-induced polyamine accumulation and malignant progression (related to Figure 4).**

**A** Total polyamine levels were measured in parental and ARID1A KO Ishikawa cells treated with either DMSO or verteporfin (2 μM) for 48 hours. Data are presented as mean ± SD (n = 3 independent biological experiments).

**B** The mRNA expression of polyamine metabolic genes was measured using RT-qPCR in parental and ARID1A-tet-on KO Ishikawa cells treated with either DMSO or verteporfin (2 μM) for 48 hours. Data are presented as mean ± SD (n = 3 independent biological experiments).

**C** CCK-8 assays were performed in Ishikawa cells treated with either DMSO or verteporfin (2 μM) for the specified duration, including both parental and ARID1A KO cells. Data are presented as mean ± SD (n = 3 independent biological experiments).

**D, E** Transwell invasion assays were performed in Ishikawa cells treated with either DMSO or verteporfin (2 μM) for 24 hours, including both parental and ARID1A KO cells. The quantitative data are presented in **E**. Data are presented as mean ± SD (n = 3 independent biological experiments). Scale bar, 100 μm. *P* values are calculated using One-way ANOVA test in (**A, B, E**) and Two-way ANOVA test in (**C**). *p<0.05, **p<0.01, ***p<0.001, n.s., not significant.


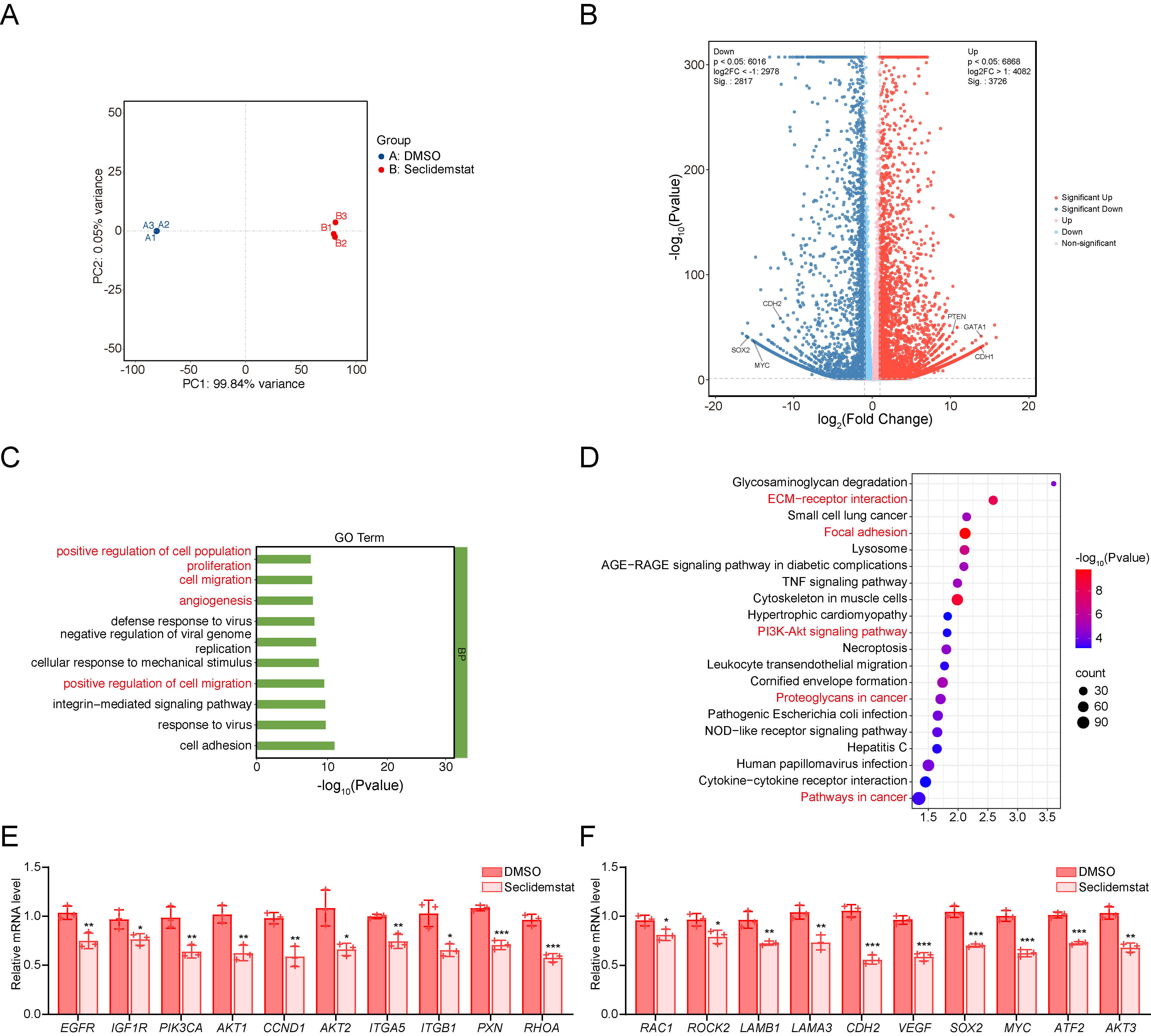


**Figure S10. LSD1 inhibition suppresses the expression of proliferation- and invasion-associated genes in ARID1A-deficient cells. (related to Figure 5).**

**A** Principal component analysis (PCA) of transcriptomic profiles of ARID1A KO Ishikawa cells treated with DMSO or seclidemstat (2 μM) for 24 hours.

**B** Volcano plot showing differentially expressed genes in ARID1A KO Ishikawa cells treated with DMSO or seclidemstat. Red indicates upregulated genes, blue indicates downregulated genes, and gray indicates non-significant genes.

**C** GO enrichment analysis highlighting downregulated biological processes in seclidemstat-treated ARID1A KO Ishikawa cells (top Biological Process terms).

**D** KEGG pathway enrichment analysis of differentially expressed genes showing that, compared with control cells, multiple pathways associated with cell proliferation and invasion are significantly downregulated in seclidemstat-treated ARID1A KO cells. Red indicates a low p value, whereas blue indicates a high p value.

**E-F** mRNA expression levels of selected differentially expressed genes were measured by RT–qPCR in ARID1A KO Ishikawa cells treated with DMSO or seclidemstat (2 μM) for 24 hours. Data are presented as mean ± SD (n = 3 independent biological experiments). P values are calculated using One-way ANOVA test. *p < 0.05, **p < 0.01, ***p < 0.001, n.s., not significant.


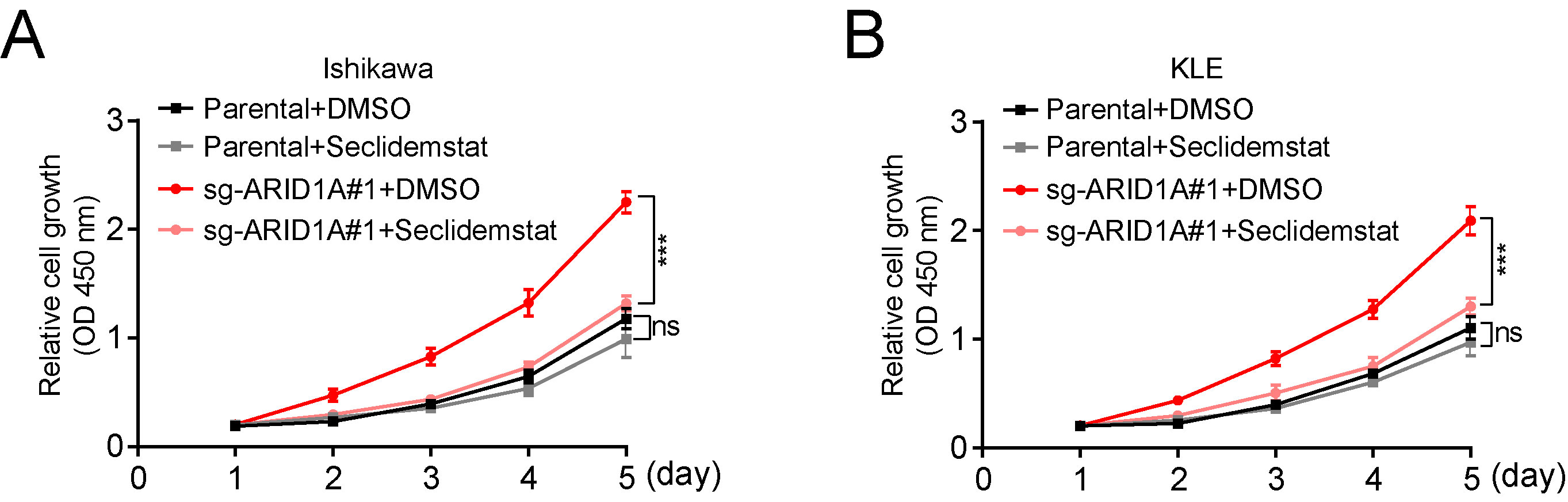


**Figure S11. Seclidemstat inhibits the growth of ARID1A KO endometrial cancer cells.**

**A** CCK-8 assays were performed in Ishikawa cells treated with DMSO or seclidemstat (2 μM) for the specified duration, including both parental and ARID1A KO cells. Data are presented as mean ± SD (n = 3 independent biological experiments).

**B** CCK-8 assays were performed in KLE cells treated with DMSO or seclidemstat (2 μM) for the specified duration, including both parental and ARID1A KO cells. Data are presented as mean ± SD (n = 3 independent biological experiments). *P* values are calculated using Two-way ANOVA test in (**A, B**). *p<0.05, **p<0.01, ***p<0.001, n.s., not significant.


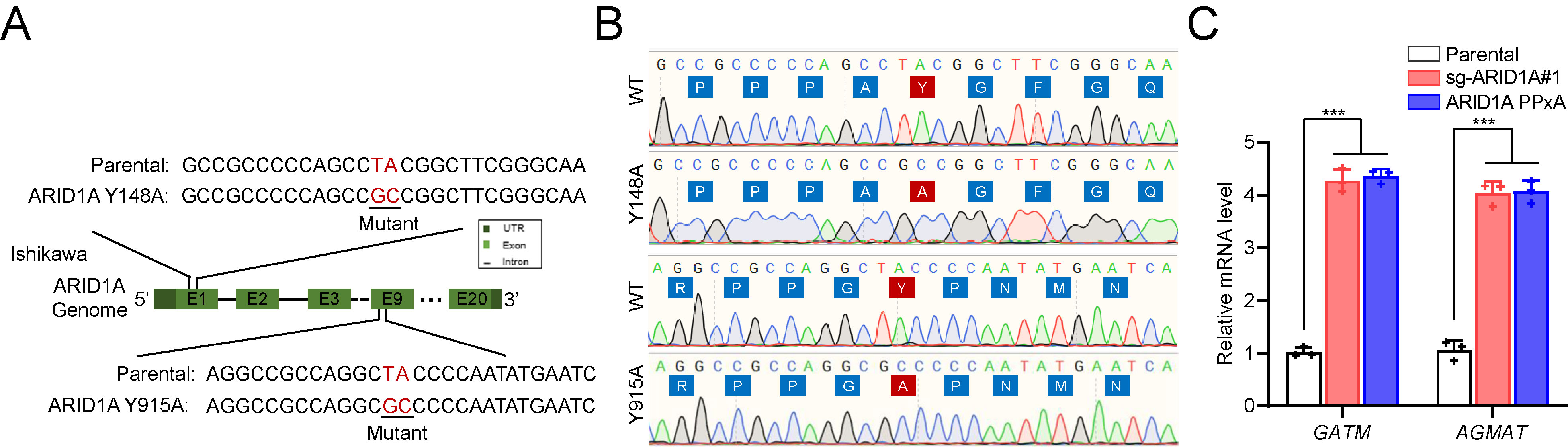


**Figure S12. ARID1A mutants showed enhanced polyamine metabolism.**

**A** Schematic representation of CRISPR/Cas9-mediated knock-in of ARID1A (Y148A, Y915A) in Ishikawa cells.

**B** Sanger sequencing confirming that the ARID1A gene was edited in Ishikawa knock-in cells.

**C** The mRNA expression of polyamine metabolic genes was measured using RT-qPCR in parental, ARID1A KO, and knock-in Ishikawa cells. Data are presented as mean ± SD (n = 3 independent biological experiments). *P* values are calculated using One-way ANOVA test in (**C**). **p* < 0.05, ***p* < 0.01, ****p* < 0.001, n.s., not significant.


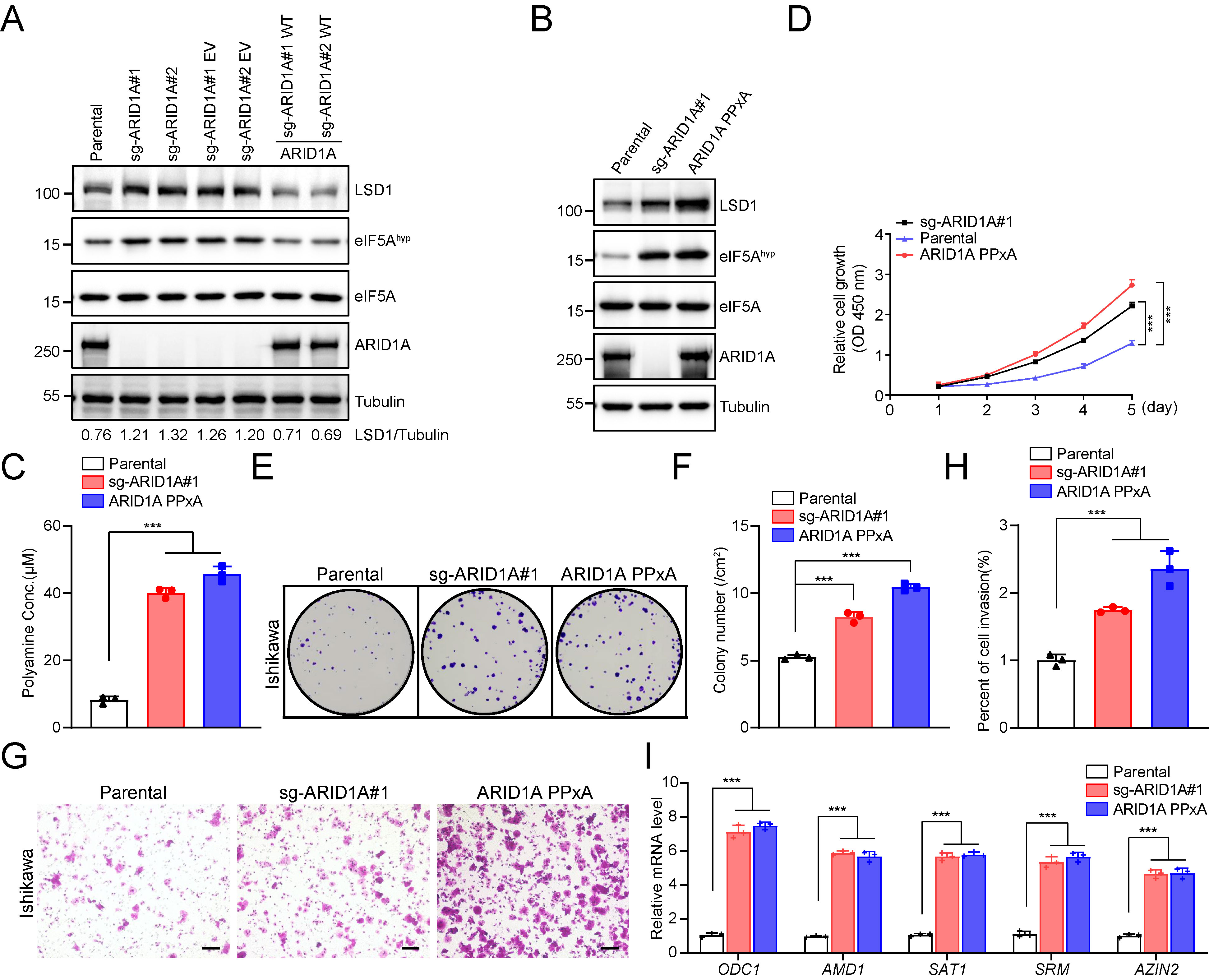


**Figure S13. ARID1A mutants showed enhanced polyamine biosynthesis and tumor malignancy.**

**A** Western blot analysis of the indicated proteins in parental and ARID1A KO Ishikawa cells stably expressing empty vector (EV) or ARID1A-WT.

**B** Western blot analysis of the indicated proteins was performed in WCLs from parental, ARID1A KO, and knock-in Ishikawa cells (Y148A, Y915A).

**C** Total polyamine levels were measured in parental, ARID1A knock-in, and KO Ishikawa cells. Data are presented as mean ± SD (n = 3 independent biological experiments).

**D** CCK-8 assays were performed in ARID1A knock-in and KO Ishikawa cells. Control cells were parental Ishikawa cells. Data are presented as mean ± SD (n = 3 independent biological experiments).

**E, F** Colony formation assays were performed in parental, ARID1A knock-in, and KO Ishikawa cells. The quantitative data are presented in **F**. Data are presented as mean ± SD (n = 3 independent biological experiments).

**G, H** Transwell invasion assays were performed in parental, ARID1A knock-in, and KO Ishikawa cells. Data are presented as mean ± SD (n = 3 independent biological experiments). Scale bar, 100 μm.

**I** The mRNA expression of polyamine metabolic genes was measured using RT-qPCR in parental, ARID1A knock-in, and KO Ishikawa cells. Data are presented as mean ± SD (n = 3 independent biological experiments). *P* values are calculated using One-way ANOVA test in (**C, F, H, I**) and Two-way ANOVA test in (**D**). **p* < 0.05, ***p* < 0.01, ****p* < 0.001, n.s., not significant.


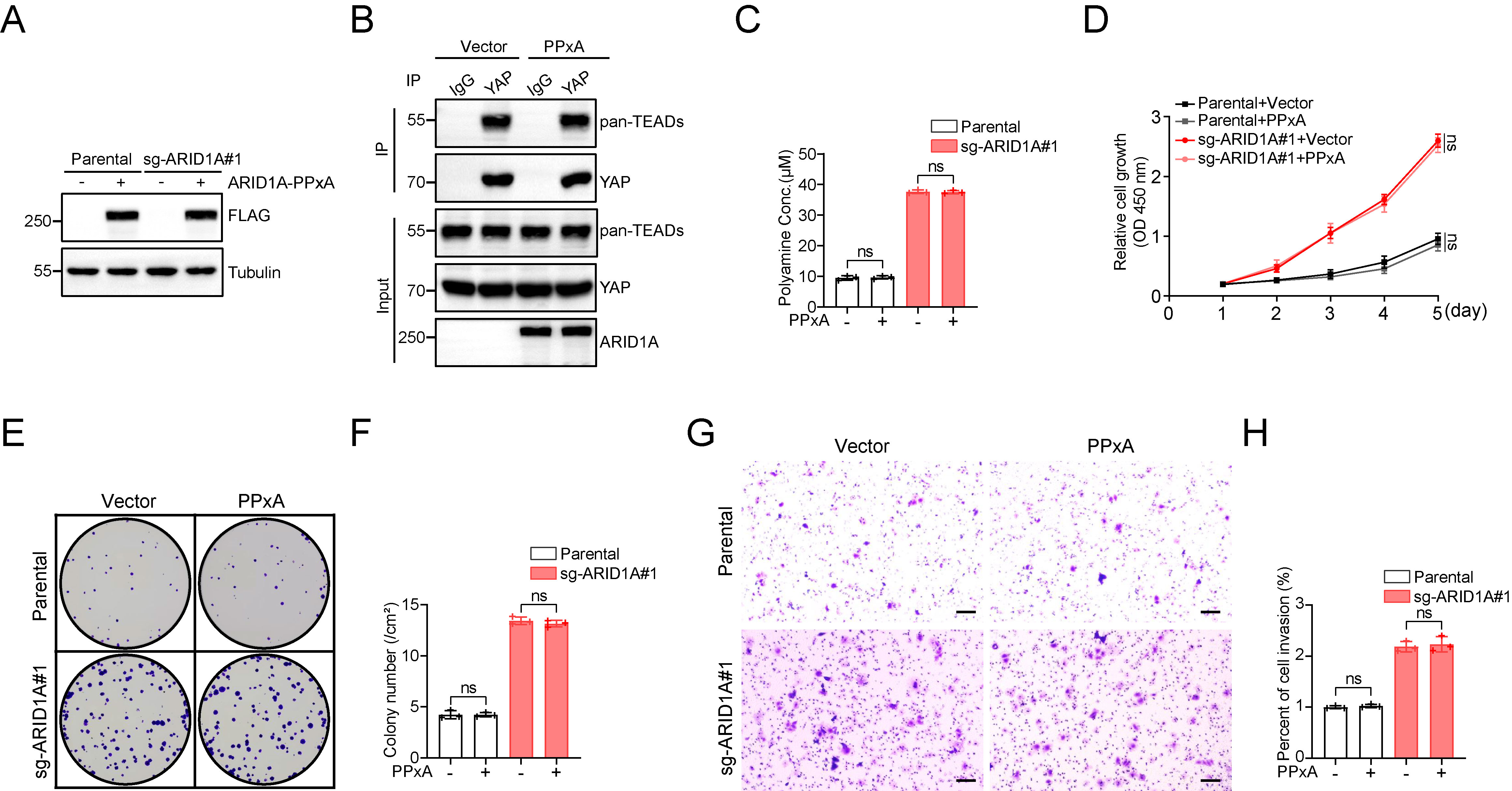


**Figure S14. Re-expression of the ARID1A-PPxA mutant in ARID1A KO cells fails to rescue polyamine accumulation, YAP–TEAD interaction, or malignant phenotypes.**

**A** Western blot analysis of ARID1A-PPxA protein expression in parental and ARID1A KO Ishikawa cells.

**B** Endogenous co-immunoprecipitation assays were performed to evaluate the interaction between YAP and TEAD following re-expression of the ARID1A-PPxA mutant in ARID1A KO Ishikawa cells, using anti-YAP antibodies, with IgG as a control.

**C** Total polyamine levels were measured in parental and ARID1A KO Ishikawa cells infected with lentivirus expressing empty vector or ARID1A-PPxA. Data are presented as mean ± SD (n = 3 independent biological experiments).

**D** Cell proliferation was assessed by CCK-8 assays in parental and ARID1A KO Ishikawa cells infected with lentivirus expressing empty vector or ARID1A-PPxA. Data are presented as mean ± SD (n = 3 independent biological experiments).

**E, F** Colony formation assays were performed in parental and ARID1A KO Ishikawa cells infected with lentivirus expressing empty vector or ARID1A-PPxA. Representative images are shown in (**E**), and quantitative analysis is shown in (**F**). Data are presented as mean ± SD (n = 3 independent biological experiments).

**G, H** Transwell invasion assays were performed in parental and ARID1A KO Ishikawa cells infected with lentivirus expressing empty vector or ARID1A-PPxA. Representative images are shown in (**G**), and quantitative analysis is shown in (**H**). Data are presented as mean ± SD (n = 3 independent biological experiments). Scale bar, 100 μm.

*P* values are calculated using One-way ANOVA test in (**C, F, H**) and Two-way ANOVA test in (**D**). **p* < 0.05, ***p* < 0.01, ****p* < 0.001, n.s., not significant.


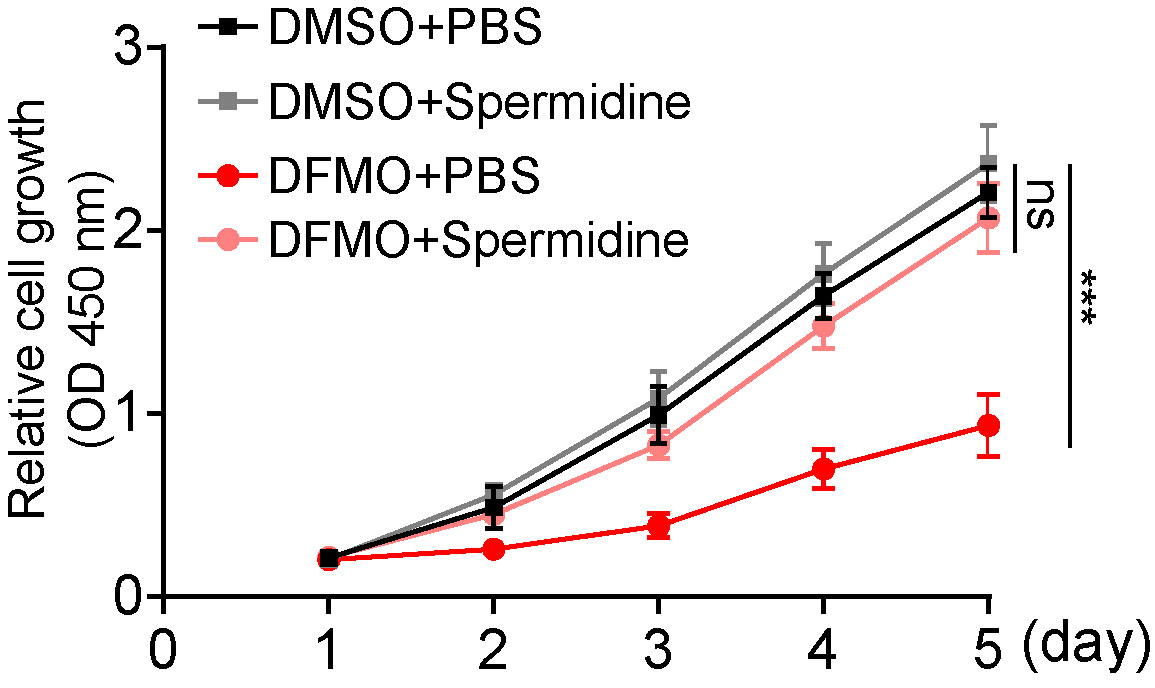


**Figure S15. Spermidine supplementation counteracted the growth-inhibitory effect of DFMO in ARID1A KO Ishikawa cells (related to Figure 6).**

CCK-8 assays were performed in ARID1A KO Ishikawa cells treated with DMSO, DFMO (2 mM), spermidine (1 mM), or DFMO (2 mM) plus spermidine (1 mM) for the specified duration. Data are presented as mean ± SD (n = 3 independent biological experiments). *P* values are calculated using Two-way ANOVA test. *p < 0.05, **p < 0.01, ***p < 0.001, n.s., not significant.


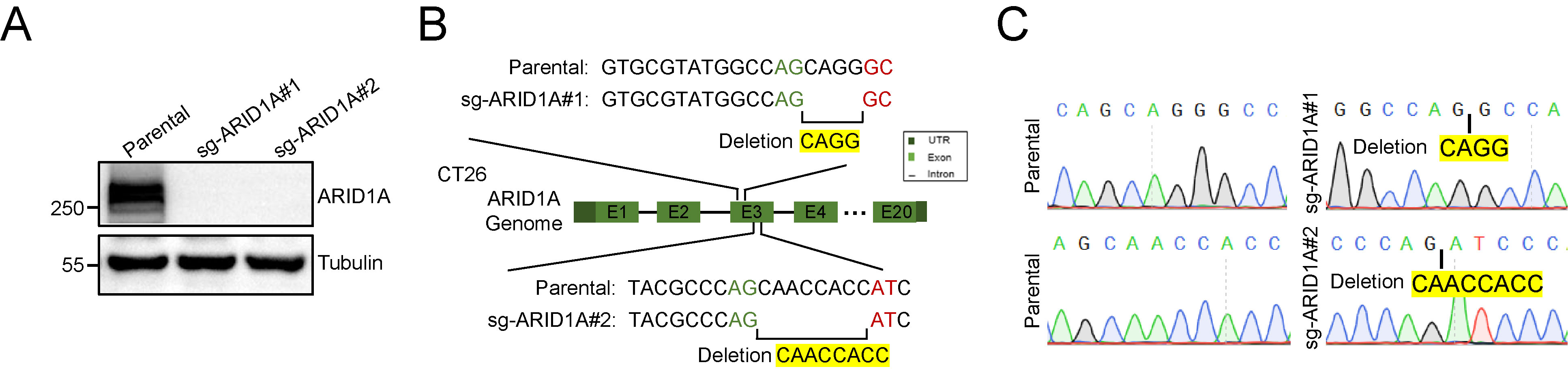


**Figure S16. Validation of ARID1A KO in CT26 cells (related to Figure 7).**

**A** Western blot analysis of the indicated proteins in WCLs from two independent ARID1A KO CT26 cell clones generated using CRISPR/Cas9 methods. Parental CT26 cells were used as a control.

**B** Schematic of CRISPR/Cas9-mediated KO of ARID1A by sgRNA#1 or sgRNA#2 in CT26 cells.

**C** Sanger sequencing confirming that the ARID1A gene was edited by sgRNA#1 or sgRNA#2 in CT26 KO cells.


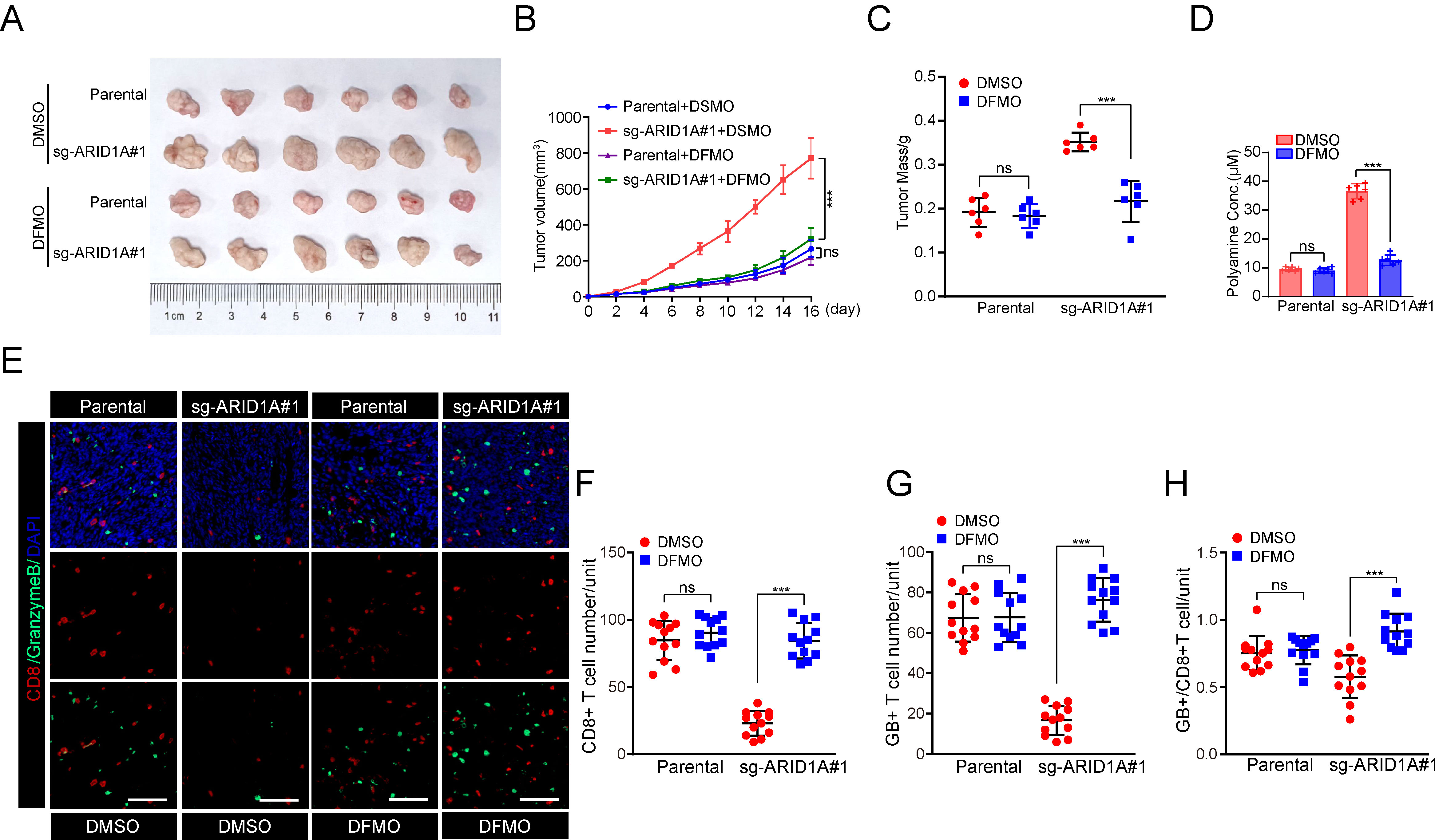


**Figure S17. Polyamines suppress the antitumor functions of CD8^+^ T cells (related to Figure 7).**

**A–C** Parental and ARID1A KO Ishikawa cells were subcutaneously injected into the right flanks of PBMC-humanized mice and treated with DFMO or DMSO every three days. Tumor growth was monitored every other day for 16 days. Each experimental group included six mice. Tumors were harvested and photographed on day 16 (**A**). Tumor volume (**B**) was recorded at the indicated time points, and tumor weight (**C**) was measured on day 16. Data are presented as mean ± SD (n = 6 mice per group). Tumor volumes were analyzed using two-way repeated-measures ANOVA with Geisser–Greenhouse correction, followed by Sidak’s multiple comparisons test.

**D** Total polyamine levels were measured in tumor tissues derived from parental and ARID1A KO Ishikawa cells. Data are presented as mean ± SD (n = 6 independent biological experiments).

**E–H** Immunostaining was performed to assess CD8 and granzyme B expression in tumor tissues. Representative images are shown in (**E**). Quantification of CD8⁺ T cells is shown in (**F**), granzyme B⁺ T cells in (**G**), and the ratio of granzyme B⁺/CD8⁺ T cells in (**H**). Data are presented as mean ± SD (n = 12 independent biological experiments). Scale bar, 100 μm. Quantification area: 412,549 μm². *P* values are calculated using One-way ANOVA test in (**C, D, F**–**H**). **p* < 0.05, ***p* < 0.01, ****p* < 0.001, n.s., not significant.


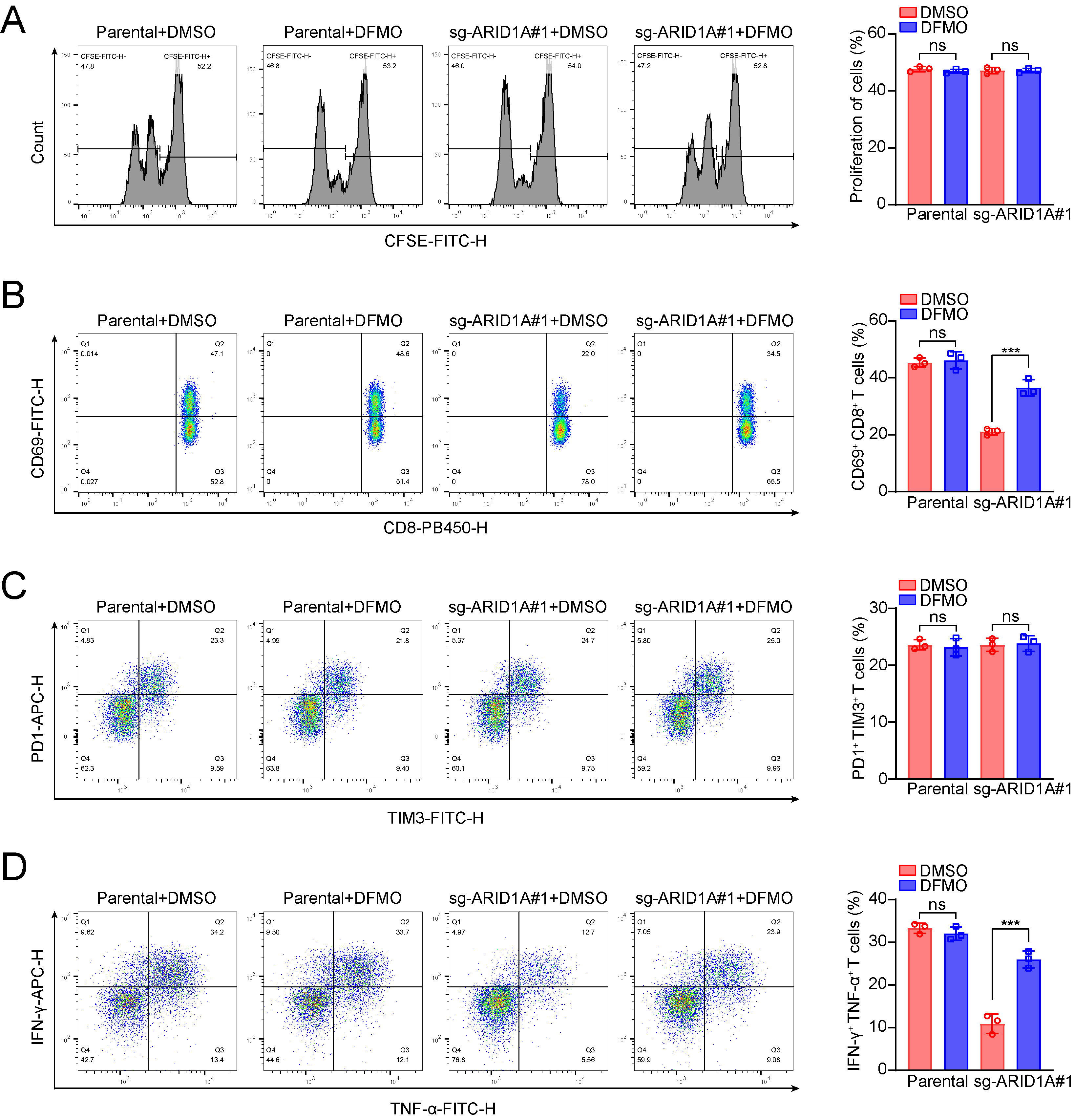


**Figure S18. DFMO enhances CD8^+^ T-cell activation and effector cytokine production in the context of ARID1A loss (related to Figure 7).**

**A** T-cell proliferation was assessed by CFSE dilution (CFSE-FITC). Representative flow cytometry plots (left) and summary quantification (right) of T-cell responses under the indicated conditions (parental or sg-ARID1A#1; vehicle control DMSO or DFMO) are shown.

**B** The frequency of activated CD69⁺CD8⁺ T cells was determined by CD69 (FITC) versus CD8 (PB450) staining. Representative flow cytometry plots (left) and summary quantification (right) under the indicated conditions are shown.

**C** The frequency of PD-1⁺TIM-3⁺ T cells was evaluated by PD-1 (APC) versus TIM-3 (FITC) staining. Representative flow cytometry plots (left) and summary quantification (right) under the indicated conditions are shown.

**D** Polyfunctional effector T cells were quantified as IFN-γ⁺TNF-α⁺ cells based on intracellular staining of IFN-γ (APC) versus TNF-α (FITC). Representative flow cytometry plots (left) and summary quantification (right) under the indicated conditions are shown.

Data are presented as mean ± SD (n = 3 independent biological experiments). *P* values are calculated using One-way ANOVA test in (**A–D**). **p* < 0.05, ***p* < 0.01, ****p* < 0.001, n.s., not significant.
