# Supplementary material for: ARID1A deficiency-driven reprogramming of polyamine metabolism promotes endometrial cancer malignancy and immune escape
Source: Cell Death Dis. 2026 Apr 8;17(1):484. doi: 10.1038/s41419-026-08722-0 (PMC13186965; doi:10.1038/s41419-026-08722-0)

Figure 1A

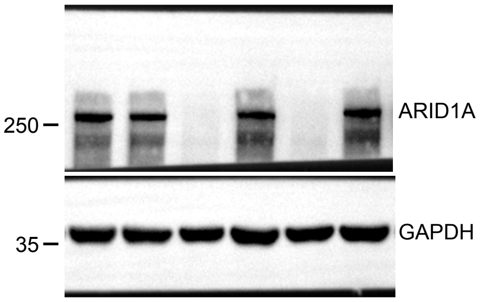

Figure 3A

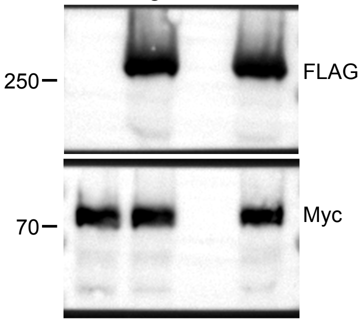

Figure 3B

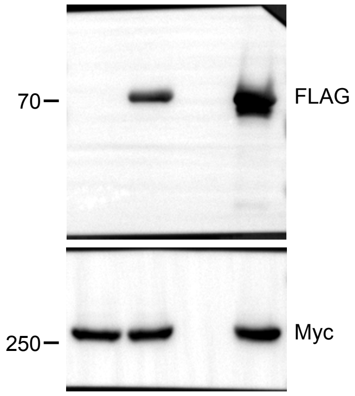

Figure 3C

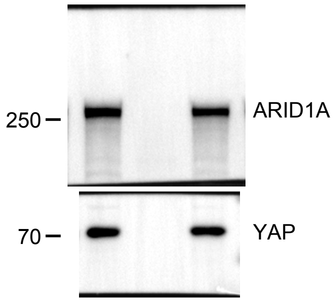

Figure 3D

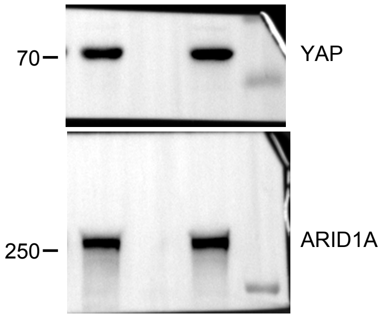

Figure 3I

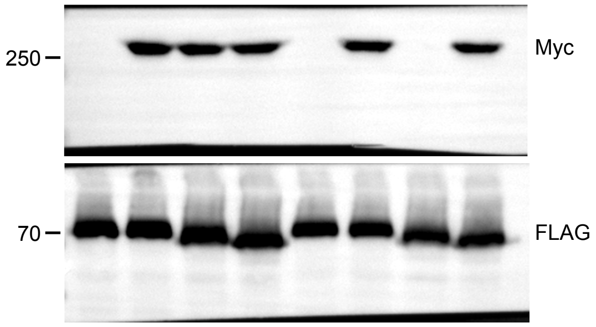

Figure 3J

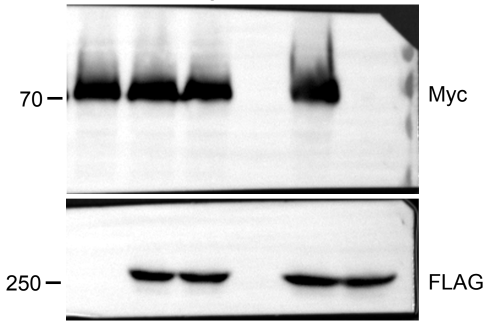

Figure 3K

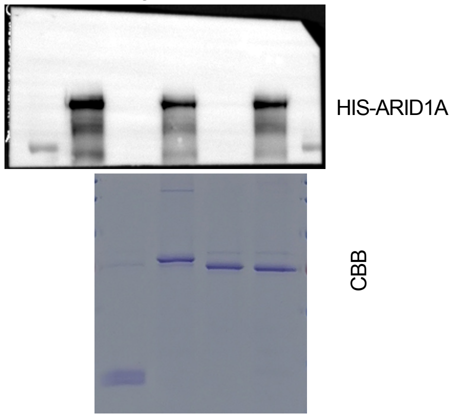

Figure 3L

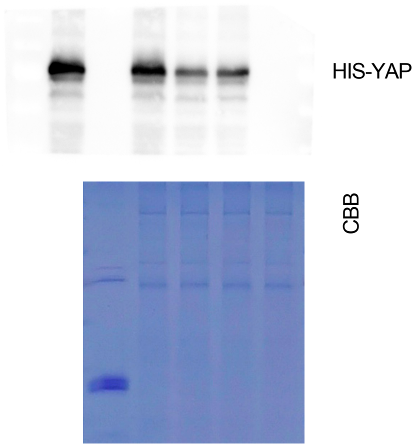

Figure 3M

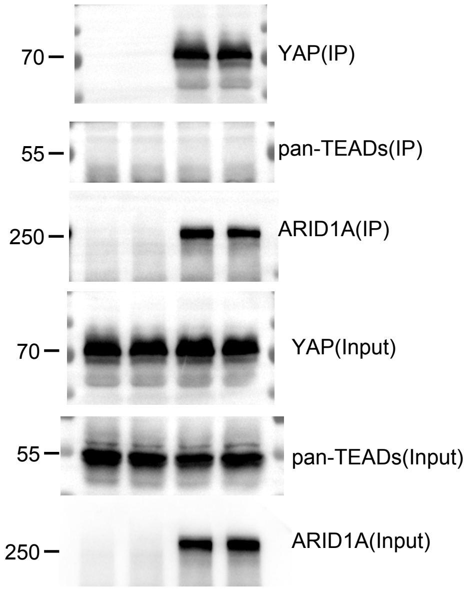

Figure 4A

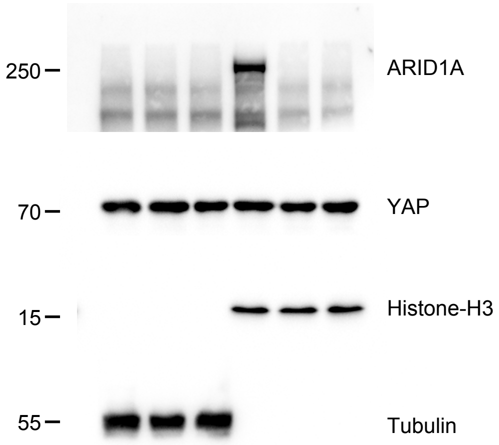

Figure 4B

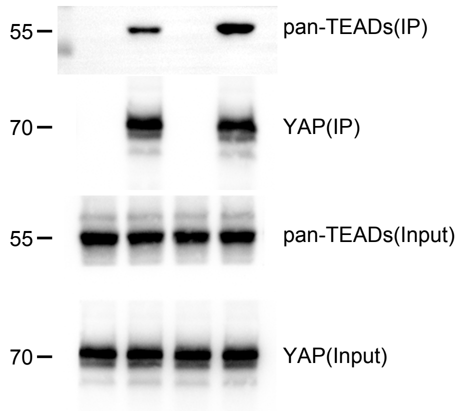

Figure 4C

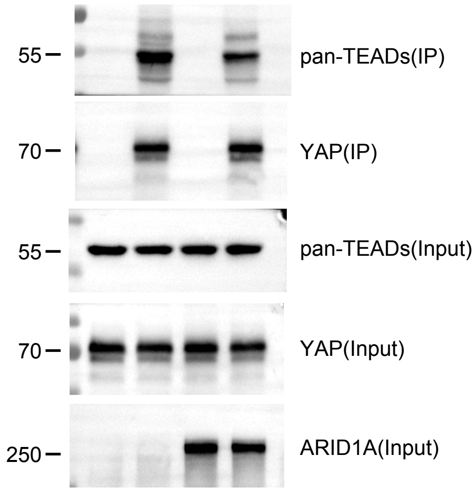

Figure 5B

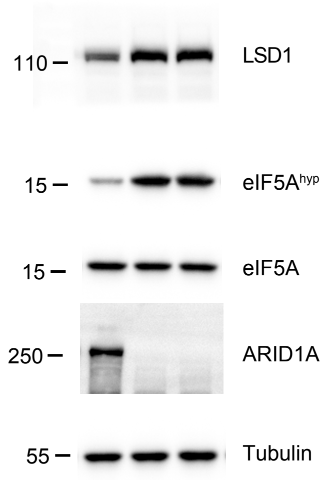

Figure 5C

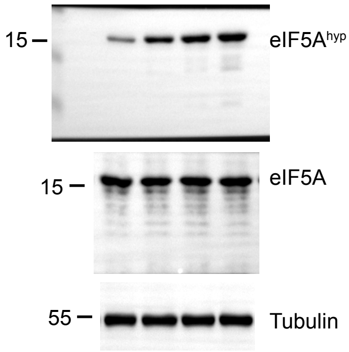

Figure 5D

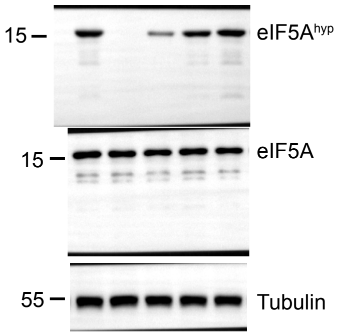

Figure 5G

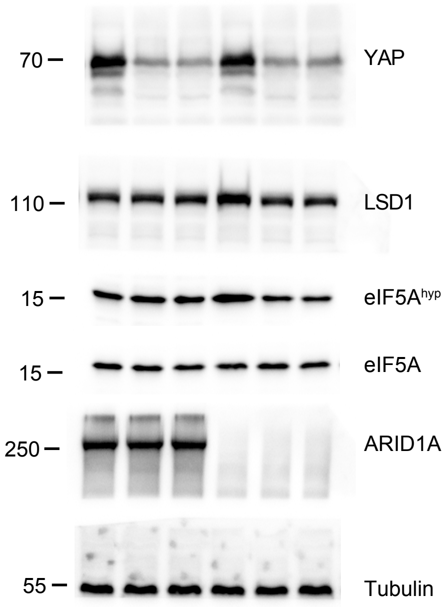

Figure 5E

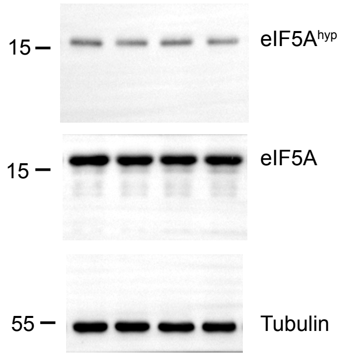

Figure 5F

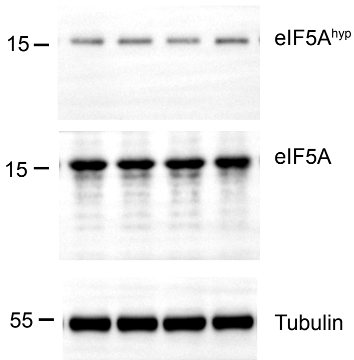

Figure 5H

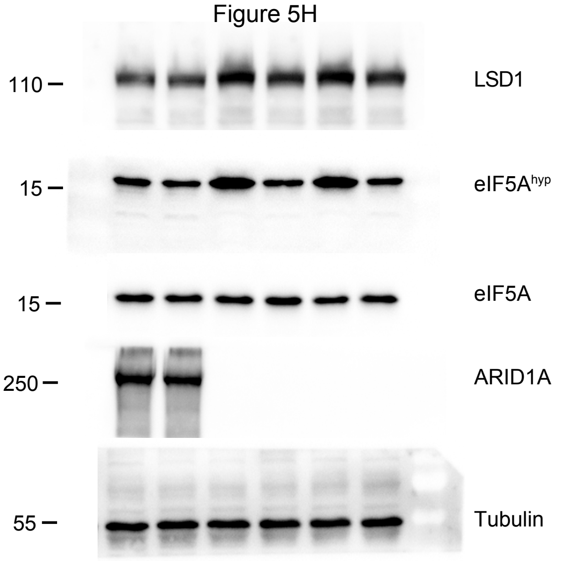

Figure 5I

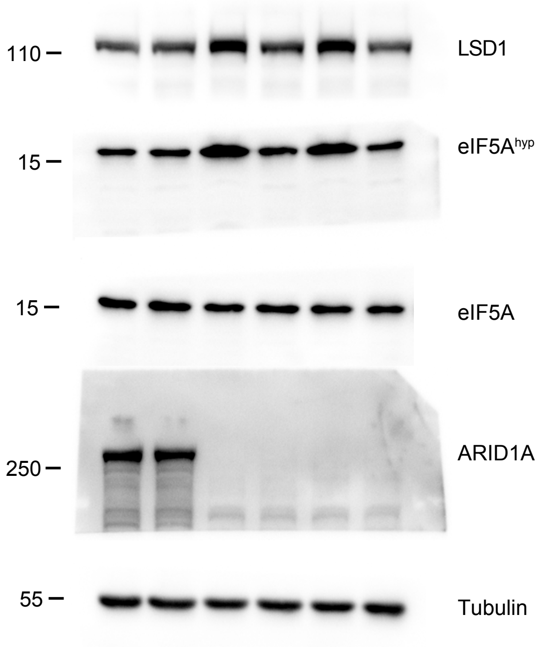

Figure 5M

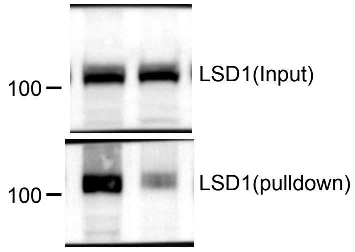

Figure 6A

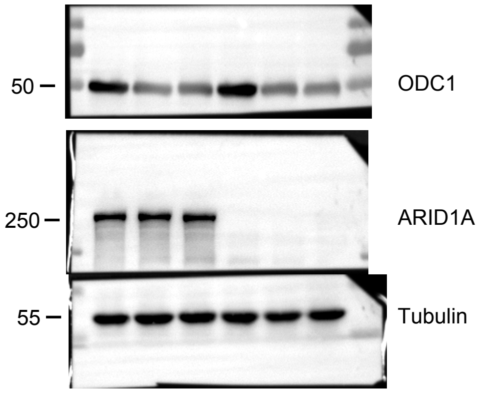

Figure S2A

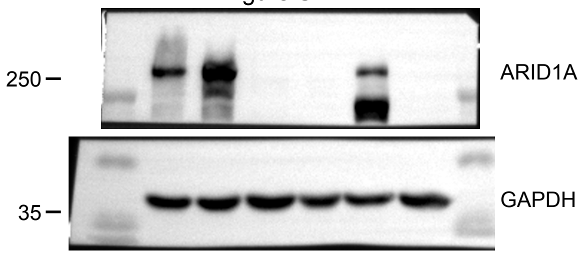

Figure S2B

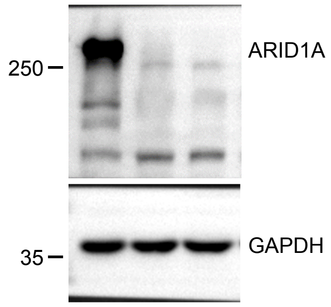

Figure S2F

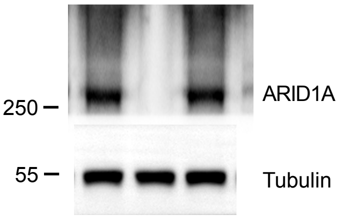

Figure S2G

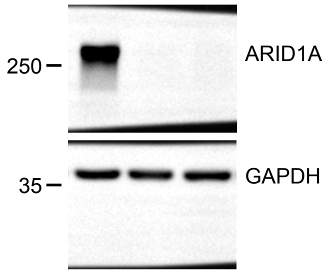

Figure S3M

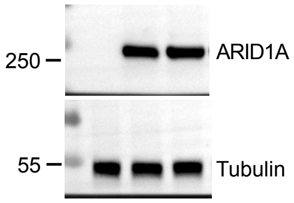

Figure S5A

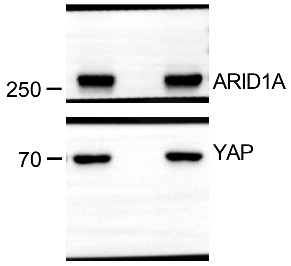

Figure S5B

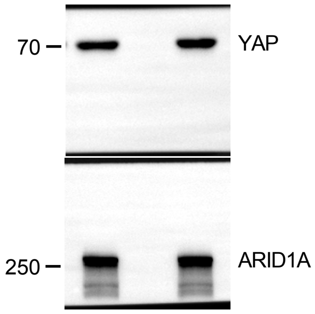

Figure S6A

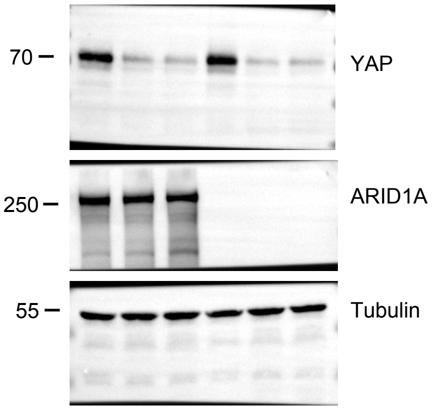

Figure S10A

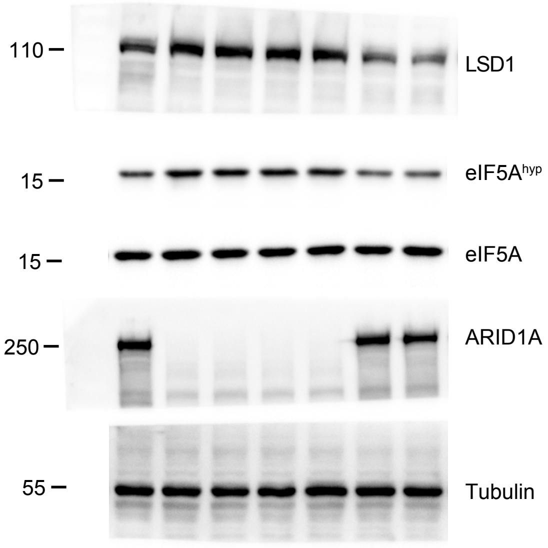

Figure S11A

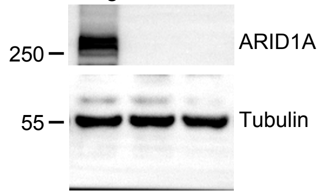

Figure S10B

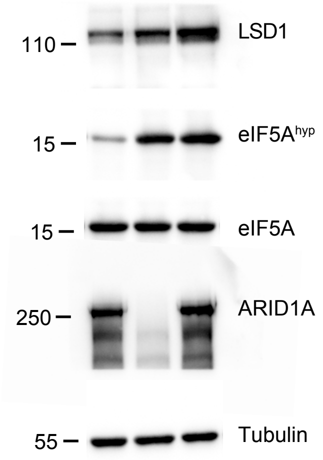

Figure S14A

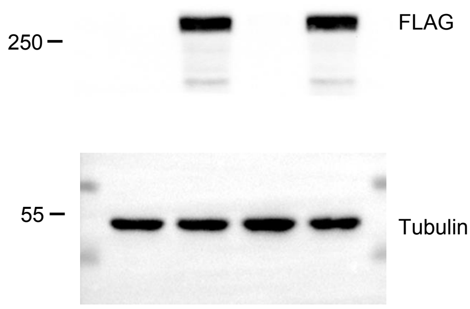

Figure S14B

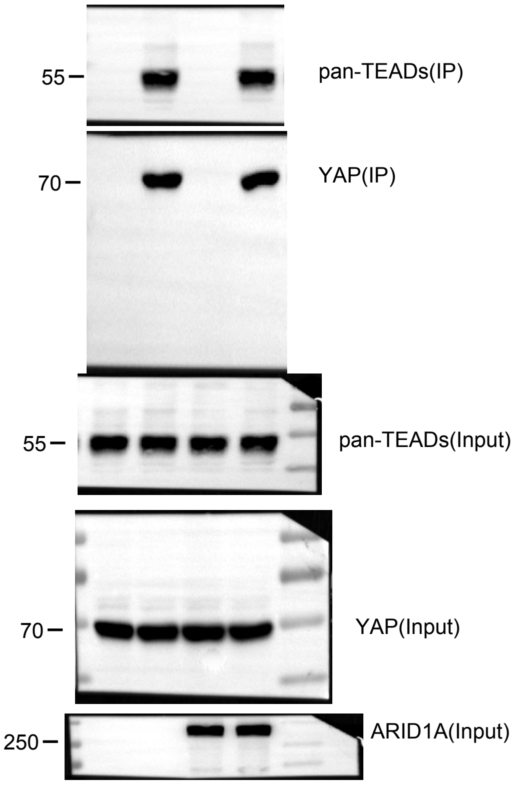

Supplement: Supplementary file 3 — Original WB data [file 41419_2026_8722_MOESM3_ESM.pdf]
